# Serum visfatin and vaspin levels in hepatocellular carcinoma (HCC)

**DOI:** 10.1371/journal.pone.0227459

**Published:** 2020-01-14

**Authors:** Monika Pazgan-Simon, Michał Kukla, Jolanta Zuwała-Jagiełło, Aleksandra Derra, Martyna Bator, Tomasz Menżyk, Andrzej Lekstan, Ewa Grzebyk, Krzysztof Simon

**Affiliations:** 1 Department of Infectious Diseases and Hepatology, Wrocław Medical University, Wroclaw, Poland; 2 Department of Gastroenterology and Hepatology, Medical University of Silesia in Katowice, Katowice, Poland; 3 Department of Pharmaceutical Biochemistry, Wroclaw Medical University, Wroclaw, Poland; 4 Medical University of Silesia in Katowice, Katowice, Poland; 5 Department of Digestive Tract Surgery, Medical University of Silesia in Katowice, Katowice, Poland; University of Navarra School of Medicine and Center for Applied Medical Research (CIMA), SPAIN

## Abstract

Hepatocellular carcinoma (HCC) is the most common liver cancer, accountable for 90% cases. Visfatin and vaspin are adipocytokines with various suggested functions and proven significant correlations between BMI and percentage of body fat. The aim was to assess visfatin and vaspin serum levels in HCC patients and controls, compare their levels in patients with different cancer etiology and grade assessed according to the Barcelona-Clinic Liver Cancer (BCLC) staging system. The additional aim was to analyze relationship between analyzed adipokines and metabolic abnormalities and liver disfunction severity. The study was performed on 69 cirrhotic patients (54 males/15 females) with HCC, aged 59.0 ± 12.1 years, and with BMI 29.0 ± 4.5 kg/m^2^ compared to 20 healthy volunteers. Serum visfatin and vaspin concentrations were significantly increased in HCC patients compared to controls (p = 0.01 and p = 0.02, respectively). Serum vaspin was significantly higher in HCC patients with viral compared to those with non-viral etiology (p = 0.02), with more evident increase in chronic hepatitis C patients (CHC). Serum visfatin levels were significantly higher in patients with higher insulin resistance (p = 0.04) and with platelets count > 100 000/mm^3^ (p<0.001). Patients with BMI >30 kg/m^2^ had markedly up-regulated vaspin levels (p = 0.04). There was no difference in vaspin and visfatin serum levels with respect to liver dysfunction and BCLC classification. In conclusion, our study revealed serum vaspin and visfatin to be significantly increased in HCC patients independently of cancer etiology compared to controls. Additionally, serum vaspin was elevated in viral disease, especially in CHC. Vaspin up-regulation can be a compensatory mechanism against IR in HCC patients. Serum visfatin and vaspin, although up-regulated, seem not to be associated with cancer grade and cirrhosis severity.

## Introduction

Hepatocellular carcinoma (HCC) is the growing problem worldwide. HCC constitutes the 6th most frequent worldwide malignancy and is 3rd cause of malignancy-related mortality. This histologic type is the most common liver cancer and is accountable for 90% cases [1].

Approximately 70–90% of HCCs are associated with chronic liver disease and cirrhosis. The risk factors for developing primary HCCs are chronic hepatitis B (CHB), chronic hepatitis C (CHC), excessive alcohol consumption, aflatoxin exposure, immune-related liver diseases and some genetic metabolic malfunctions (e.g. hemochromatosis) [2]. The risk of HCC is increased when the patients have more than one of HCC risk factors. Significant risk factors for the development of HCC are also metabolic syndrome, obesity and type 2 diabetes mellitus (T2DM). Many factors influence the development of nonalcoholic fatty liver disease (NAFLD), non-alcoholic steatohepatitis (NASH) and carcinogenesis in the course of obesity and related metabolic abnormalities. They comprise common pathway of intracellular insulin signaling and affecting insulin sensitivity. Increasing insulin resistance (IR) is related to actuation of inflammatory cascade and angiogenesis and acceleration of cell proliferation. Evident dysregulation of serum cytokines levels, altered gut microbiota and bile acids composition are also connected to metabolic syndrome. Dysregulation of adipose tissue derived hormones (adipocytokines/adipokines) might also be involved in obesity-related liver carcinogenesis [3–7]. Adipokines disbalance, such as dysregulation in level of adiponectin, leptin, resistin, chemerin, visfatin and some others, have been identified as a factor increasing fibrosis progression, inflammation and steatosis in chronic liver diseases including NAFLD/NASH, CHB and alcoholic liver disease (ALD) [4–9].

Visfatin/nicotinamide phosphoribosyltransferase (NAMPT)/pre-B-cell colony-enhancing factor (PBEF) is abundantly expressed in the visceral fat of humans and mice. Increased levels of visfatin are positively correlated with body mass index (BMI) and the size of visceral fat deposits [10]. Visfatin exerts an insulin mimetic activity, which is mediated by a noncompetitive, distinct binding site on the insulin receptor and can induce the phosphorylation of insulin receptor, insulin receptor substrate 1 (IRS1) and IRS2. It has also the ability to regulate many signaling pathways—phosphatidylinositol 3-kinase (PI3K)/protein kinase B (Akt), extracellular signal–regulated kinas 1/2 (ERK 1/2), mitogen-activated protein kinase (MAPK) and signal transducer and activator of transcription 3 (STAT3) [11]. Visfatin induces production of vascular endothelial growth factor (VEGF), matrix metalloproteinase-2 (MMP-2) and MMP-9 in vascular endothelial cells. Possibly through activation of VEGF–MMP pathways visfatin induces endothelial cells migration, tube formation, and angiogenesis [7,12]. Moreover, visfatin is a proinflammatory cytokine, which can stimulate the expression of tumor necrosis factor α (TNFα), interleukin 1B (IL-1B), IL-6 and promote the differentiation of B-cells [13]. Dysregulation in visfatin concentration has many pleiotropic and pathophysiological effects, and is associated with various clinical conditions including obesity, T2DM [13]. In addition, previous studies have shown positive correlation between increased circulating level of visfatin and the occurrence, and progression of different types of malignancies. The connection between visfatin and carcinogenesis has been observed in colorectal cancer (CRC), gastric, prostate, breast, ovarian, pancreatic and oral cancers [11,14]. Positive correlation has been found between serum visfatin levels and HCC tumor size as well as the presence of NAFLD [15, 16].

Another novel adipokine, vaspin (visceral adipose tissue-derived serine protease inhibitor), was firstly identified in obese OLETF rats [17]. It has been isolated from both visceral and subcutaneous white adipose tissue. Significant correlations between visceral vaspin expression and such parameters as BMI, percentage of body fat and the level of plasma glucose following 2-h oral glucose tolerance testing were proved [7]. Additionally, its subcutaneous expression significantly correlated with waist-to-hip ratio, fasting plasma insulin concentration and glucose infusion rate during the steady state of an euglycemic–hyperinsulinemic clamp [8, 18]. Vaspin expression is strongly dependent on insulin sensitivity and percentage of body fat. Recent studies also found that vaspin gene in human adipose tissue and circulating vaspin levels were up-regulated by obesity-associated diseases and T2DM, probably proving its compensatory role in that states [18]. Furthermore, vaspin has been shown to significantly improve glucose tolerance and insulin sensitivity in murine, suggesting that it may represent an insulin-sensitizing adipokine [19]. Vaspin has been found to down-regulate the expression of profibrogenic and proinflammatory agents such as leptin, TNFα and resistin. Progressive, significant liver fibrosis is associated with increase of the serum vaspin level in CHC [20].

Pointing to a wide spectrum of visfatin and vaspin activities the aim of the study was to measure the serum levels of visfatin and vaspin in HCC patients with subsequent differentiation with respect to disease etiology and the severity of liver dysfunction. The additional purpose was to assess relationship between their serum levels and HCC grade and progression. Finally, we tried to assess relationship between visfatin and vaspin levels and T2DM and IR in HCC patients.

## Materials and methods

The research was carried on adults. They all agreed formally to take part in the project. The patients who took part in the project were either hospitalized at our unit or had been entered into our database and thus signed into the project.

The study was performed on 69 cirrhotic patients (54 males/15 females) with contrast computed tomography (CT) and/or magnetic resonance imaging (MRI) confirmed HCC, aged 59.0 ± 12.1 years, and with BMI 29.0 ± 4.5 kg/m^2^. The number of patients was limited to those diagnosed, additionally we excluded patients with no permission for contribution in research, insulin dependent diabetes mellitus, other malignancies, chronic heart failure, chronic kidney disease, psychiatric disorders, alcohol consumption of more than 20 g/day, thyroid disorders. The exclusion criteria were also cirrhosis due to primary sclerosing cholangitis (PSC), primary biliary cholangitis (PBC), autoimmune hepatitis (AIH) and drug induced liver disease. The control group comprised 20 healthy volunteers (10 females/10 males), with normal alanine aminotransferase (ALT) activity, alcohol consumption of less than 20 g/day, without anti-hepatitis C virus (anti HCV) antibodies, without hepatitis B surface antigen (HBsAg) and HIV-negative, age 40.6 ± 5.5 years and BMI 25.4 ± 4.1 kg/m^2^. The assessed clinical and laboratory parameters in both groups are presented in Table 1.

**Table 1:** The baseline characteristics and laboratory data of patients and healthy volunteers.

Parameter	HCC patients (N = 69)	Healthy volunteers (N = 20)	p
Age [years]	59.0 ± 12.1	40.6 ± 5.5	0.65
BMI [kg/m2]	29.0 ± 4.5	25.4 ± 4.1	0.25
Waist grith [cm]	102.0 ± 12.5	78.2 ± 5.4	0.03
Vaspin [ng/mL]	0.18 ± 0.93	0.10 ± 0.08	0.05
Visfatin [ng/mL]	4.50 ± 1.79	2.62 ± 1.47	0.04
Fasting insulin [ng/mL]	0.89 ± 0.91	0.44 ± 0.15	0.002
Fasting insulin [mIU/mL]	20.2 ± 20.9	10.2 ± 3.45	0.002
WBC [106/μL]	4.30 ± 2.46	5.20 ± 0.85	0.35
HGB [mg/dL]	12.2 ± 2.45	14.5 ± 1.41	0.14
PLT [106/μL]	88.5 ± 85.0	220.5 ± 50.7	0.02
ALT [IU/L]	45.0 ± 48.5	22.6 ± 13.6	0.01
AST [IU/L]	63.0 ± 65.1	24.0 ± 9.8	0.008
FA [IU/L]	110.0 ± 89.4	65.5 ± 25.6	0.01
GGTP [IU/L]	91.0 ± 194.6	24.6 ± 15.7	<0.001
Fasting glucose [mg/dL]	106.8 ± 43.9	89.9 ± 9.5	0.02
Urea [mg/dL]	35.3 ± 30.5	31.6 ± 9.4	0.45
Creatinine [mg/dL]	0.79 ± 0.27	0.78 ± 0.13	0.09
Bilirubin [mg/dL]	1.58 ± 18.1	1.10 ± 0.45	0.01
Cholesterol [mg/dL]	147.0 ± 50.1	169.7 ± 50.5	0.65
Tryglicerides [mg/dL]	105.0 ± 78.64	128.6 ± 21.4	0.31
HDL [mg/dL]	37.4 ± 31.0	44.9 ± 8.1	0.42
Prothrombin index [%]	75.0 ± 14.1	95.7 ± 5.3	0.03
Total protein [g/dL]	7.10 ± 0.94	7.30 ± 0.31	0.12
Albumine [g/dL]	3.10 ± 0.59	4.85 ± 0.34	0.04
AFP [ng/mL]	16.0 ± 1145.0	12.7 ± 1.80	<0.001
CEA [ng/mL]	2.86 ± 2.91	2.45 ± 2.56	0.54
CA 19.9 [U/mL]	15.1 ± 89.3	19.7 ± 4.6	0.28
HOMA-IR	5.47 ± 8.10	2.53 ± 0.09	<0.001

Vaspin and visfatin serum concentrations were assessed in duplicate by an immunoenzymatic method with commercially available enzyme immunoassay (EIA) or enzyme-linked immunosorbent assay (ELISA) kits: Human Vaspin ELISA Kit (sensitivity 0.01 ng/mL; Intra-assay CV 6.5–8.7%; Inter-assay CV 5.8–9.5%; catalogue No. RD191097200R; BioVendor–Laboratorni medicina a.s.); and Visfatin (NAMPT) Human ELISA Kit, (sensitivity 30 pg/mL; Intra-assay CV 2.3–9.1%; Inter-assay CV 4.6–7.2%; catalogue No. RAG004R, BioVendor–Laboratorni medicina a.s.). Insulin concentration was measured using a Diametria Insulin EIA Kit (catalogue No. DKO076; Diametra S.r.l).

The remaining biochemical parameters were measured using routine methods. The upper limit of normal (ULN) of ALT activity was set at 38 IU/L and the ULN of aspartate aminotransferase (AST) activity at 40 IU/L. Insulin resistance was calculated according to the homeostasis model assessment for IR (HOMA-IR) by the formula: fasting insulin level (mUI/L) × fasting glucose level (mg/dL)/405. Because HOMA-IR was up-regulated in most of analyzed patients, for further analysis patients were divided into two subgroups with HOMA-IR value below 4 and equal to or above 4.

For further analysis, we defined two subgroups with respect to BMI value: BMI < 30 and BMI ⩾ 30 kg/m^2^. Patients with a BMI of ⩾ 30 kg/m^2^ were classified as being obese based on the guidelines of the World Health Organization [21]. The diagnosis of T2DM was made according to the World Health Organization criteria [22], with a value of fasting blood glucose level of ⩾126 mg/dL on at least two occasions, or ongoing treatment with hypoglycemic agents.

All of the outcomes were assessed according to Child-Turcotte-Pugh (CTP) score and Barcelona Clinic Liver Cancer (BCLC) classification. Child-Turcotte-Pugh score in actual modification is a predictor of prognosis of liver cirrhosis with some limitation. It consist of 3 continuous variables (bilirubin, albumin, prothrombin time), and 2 discrete variables (ascites, encephalopathy). Obtained CTP scores correspond to the total points of each item. The sum of these point characterizes patient to one out of three classes: A (5–6 points), B (7–9 points), C (10–15).

The study was approved by the Ethical Committee of the Medical University in Wroclaw and conformed to the ethical guidelines of the Declaration of Helsinki. Informed written consent was obtained for the whole study series.

The statistical analysis was performed with STATISTICA 10.0 (StatSoft Polska Sp. z o.o., Cracow, Poland). The data were expressed as median ± standard deviation (SD). The Shapiro–Wilk test was used to evaluate the distribution. The statistical significance of the difference in studied variables were tested using the Mann–Whitney U-test and ANOVA rang Kruskal–Wallis tests for independent groups. Correlations were analyzed with the Spearman rank correlation coefficient. Statistical significance was defined as values of p<0.05.

## Results and discussion

### Baseline characteristics and laboratory data of HCC patients and control group

The baseline characteristics and laboratory data of 69 cirrhotic patients with HCC (54 men and 15 women, mean age 59.0±12.1 years) and controls have been summarized in Table 1. Mean BMI was 29.0±4.5 kg/m^2^. Viral cirrhosis was presented among 43 patients (62.3%). Chronic hepatitis C confirmed by the presence of HCV genotype 1b RNA was diagnosed in 35 patients. The remaining 8 of those patients were diagnosed with positive serologic markers for hepatitis B. NAFLD/NASH derived cirrhosis was found in 26 patients. Our study included 27 patients with T2DM and 20 patients with hypertension (HT).

### Characteristics of the study group according to child-turcotte-pugh score and barcelona clinic liver cancer

All 69 HCC patients had liver cirrhosis. Patients were divided according to CTP score into 3 groups: class A including 36 patients, class B– 27 patients and class C– 6 patients. All HCC cases were divided into 4 four groups according to BCLC classification according to disease stage. Stage A was found in 12, stage B in 20, stage C in 16 patients. None of analyzed patients were in stage D.

### Comparison of serum visfatin and vaspin between HCC patients and control group

**Fig 1:**
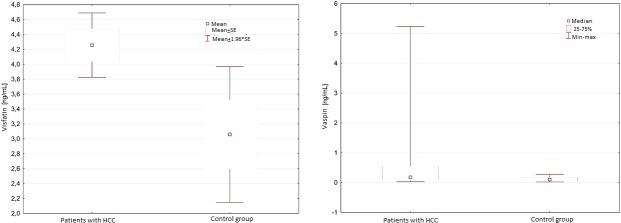
**A.** Serum visfatin concentration in HCC patients compared to healthy volunteers. **B.** Serum vaspin concentration in HCC patients compared to healthy volunteers.

Serum visfatin concentration was significantly higher in HCC patients compared to healthy volunteers (4.50+1.79 vs 2.62+1.47 ng/mL; p = 0.04) (Fig 1A). The serum vaspin level was also significantly higher in HCC patients than in control group (0.18+0.93 vs 0.10+0.08 ng/mL; p = 0.05) (Fig 1B). In addition, the serum vaspin concentration was significantly lower among men compared to women (0.13±1.02vs 0.42±0.53 ng/mL; p = 0.01).

### Comparison of HCC patients with respect to etiology, metabolic abnormalities and T2DM presence, BCLC and CTP scores

Pointing to disease etiology, serum concentration of vaspin was significantly higher in HCC patients with viral compared to those with non-viral etiology (0.23±0.88 vs 0.10±1.02 ng/mL; p = 0.02) (Table 2). There were no similar findings observed in visfatin serum level in those both groups (4.62±1.84 vs 4.48 ± 1.73 ng/mL; p = 0.59). Furthermore, the elevation of serum vaspin level became more evident when only CHC patients were compared to those with non-viral etiology of liver disease (0.41±0.94 vs 0.45±1.02 ng/mL; p = 0.003). In addition, viral etiology of HCC and presence of HCV RNA were associated with higher serum concentration of alpha-fetoprotein (AFP) (37.7±1414.2 vs. 4.82±13.1 ng/mL; p<0.001) and carcinoembryonic antigen (CEA) (3.11±3.36 vs. 2.30±1.61 ng/mL; p = 0.03).

**Table 2:** Serum concentration of vaspin, visfatin, AFP, CEA, fasting insulin, fasting glucose and HOMA-IR in HCC patients with viral etiology compared to those with non-viral etiology of HCC, and HCV positive patients compared to those with non-viral liver disease.

**Parameter**	**Viral etiology (N = 43)**	**HCV and HBV negative (N = 26)**	**P**
Vaspin [ng/mL]	0.23 ± 0.88	0.10 ± 1.02	0.02
Visfatin [ng/mL]	4.62 ± 1.84	4.48 ± 1.73	0.59
AFP [ng/mL]	37.7 ± 1414.2	4.82 ± 13.1	< 0.001
CEA [ng/mL]	3.11 ± 3.36	2.30 ± 1.61	0.03
Fasting insulin [mIU/mL]	19.7 ± 22.0	21.3 ± 19.5	0.81
Fasting glucose [mg/dL]	106.8 ± 40.9	111.9 ± 48.5	0.002
HOMA-IR	5.30 ± 6.72	5.49 ± 10.07	0.67
**Parameter**	**HCV positive (N = 35)**	**HCV and HBV negative (N = 26)**	**P**
Vaspin [ng/mL]	0.41 ± 0.94	0.10 ± 1.02	0.003
Visfatin [ng/mL]	4.43 ± 1.93	4.48 ± 1.73	0.66
AFP [ng/mL]	37.9 ± 1262.9	4.82 ± 13.1	< 0.001
CEA [ng/mL]	3.32 ± 3.60	2.30 ± 1.61	0.01
Fasting insulin [mIU/mL]	21.0 ± 19.6	21.3 ± 19.5	0.96
Fasting glucose [mg/dL]	112.3 ± 42.1	111.9 ± 48.5	0.8
HOMA-IR	6.11 ± 6.68	5.50 ± 10.10	0.91

No difference was observed in visfatin and vaspin serum concentrations among patients with and without T2DM (4.81±1.50 vs 4.13±1.93 ng/mL; p = 0.23; and 0.15±1.0 vs 0.19±0.87 ng/mL; p = 0.65, respectively). However, fasting insulin level was significantly higher in HCC patients compared to control group (20.2+20.9 vs 10.2+3.45 mIU/mL, p = 0.002).

Patients were divided according to HOMA-IR value into two groups: first group including 25 patients with HOMA-IR < 4 and second one encompassing 44 subjects with HOMA-IR ≥ 4. Serum visfatin levels were significantly higher in patients with higher IR (p = 0.04), whereas there was no difference with respect to serum vaspin concentration (p = 0.76) (Table 3).

**Table 3:** Serum glucose, insulin, vaspin and visfatin levels according to HOMA-IR value.

Parameter	HOMA-IR < 4	HOMA-IR ≥ 4	P
Fasting glucose [mg/dL]	96.6 ± 17.6	134.2 ± 48.5	< 0.001
Insulin [mIU/L]	11.0 ± 3.60	35.8 ± 21.4	< 0.001
Vaspin [ng/mL]	0.14 ± 1.00	0.18 ± 0.88	0.76
Visfatin [ng/mL]	4.81 ± 1.60	4.01± 1.83	0.04

We also compared groups of patients with HCC according to BCLC classification. There was no difference in visfatin and vaspin serum levels (p = 0.90 and p = 0.53). Serum vaspin and visfatin concentration also did not differ between HCC patients with various CTP score (p = 0.59 and p = 0.11). No significant difference was also observed in fasting insulin level, fasting glucose level and HOMA-IR in any of those BCLC groups of HCC patients. (Table 4)

**Table 4:** Serum concentration of vaspin, visfatin, fasting insulin, fasting glucose and HOMA-IR in groups of patients according to BCLC classification and child-pugh score.

**Parameter**	**BCLC A**	**BCLC B**	**BCLC C**	**P**
Vaspin [ng/mL]	0.53 ± 1.41	0.22 ± 0.58	0.19 ± 1.22	0.53
Visfatin [ng/mL]	4.67 ± 1.89	4.43 ± 1.59	4.77 ± 2.20	0.9
Fasting insulin [mIU/mL]	25.7 ± 21.0	18.8 ± 20.7	20.5 ± 22.0	0.16
Fasting glucose [mg/dL]	119.4 ± 28.3	106.5 ± 50.3	95.4 ± 42.1	0.25
HOMA-IR	7.15 ± 6.80	4.98 ± 7.22	5.37 ± 5.40	0.36
**Parameter**	**CTP A**	**CTP B**	**CTP C**	**p**
Vaspin [ng/mL]	0.17 ± 0.90	0.26 ± 0.60	0.17 ± 1.92	0.59
Visfatin [ng/mL]	4.63 ± 1.60	3.26 ± 2.00	5.34 ± 1.37	0.11
Fasting insulin [mIU/mL]	16.6 ± 20.9	23.9 ± 20.0	25.0 ± 26.0	0.29
Fasting glucose [mg/dL]	106.8 ± 40.2	111.1 ± 46.7	111.0 ± 58.4	0.98
HOMA-IR	4.91 ± 6.90	6.17 ± 9.60	10.7 ± 8.00	0.35

According to platelets count patients were divided into two groups: first group including 28 subjects with platelets count > 100 000/mm^3^ and second one incorporating 41 patients with platelets ≤100 000/mm^3^. Serum visfatin levels were significantly higher among patients with platelets count >100 000/mm^3^ compared to those with platelets count ≤ 100 000/mm^3^ (5.27±1.40 vs 3.31±1.70 ng/mL; p<0.001) (Table 5). No statistically significant difference was observed in vaspin serum concentration among those groups of patients (0.12±1.00 vs 0.20±0.90 ng/mL; p = 0.23). Among group of subjects with platelets count > 100 000/mm^3^ compared to those with platelets count ≤100 000/mm^3^ significantly higher serum AFP (37.8±1294.2 vs 7.91±1036.2 ng/mL; p<0.001), albumin (3.40±0.70 vs 3.00±0.50 ng/mL; p = 0.02), total cholesterol (166.8±55.0 vs 143.6±40.5ng/mL; p = 0.05) and triglycerides concentrations (123.6±42.0 vs 94.6±96.2 ng/mL; p = 0.01) were found. Inverse results were observed with respect to serum insulin concentrations (12.5±15.9 vs 25.5±22.7 mIU/L; p = 0.003), and HOMA-IR value (2.82±6.40 vs 7.06±8.80; p = 0.001) when compared those groups.

**Table 5:** Serum concentration of vaspin, visfatin, fasting insulin, fasting glucose, cholesterol, triglycerides, albumin, AFP and HOMA-IR in group of patients with platelets count > 100 000 compared to those with platelets count ≤ 100 000/mm^3^.

Parameter	Plt > 100 000/mm3	Plt <100 000/mm3	P
Vaspin [ng/mL]	0.12 ± 1.00	0.20 ± 0.90	0.23
Visfatin [ng/mL]	5.27 ± 1.40	3.31 ± 1.70	< 0.001
Fasting insulin [mIU/L]	12.5 ± 15.9	25.5 ± 22.7	0.003
Fasting glucose [mg/mL]	101.5 ± 40.5	119.4 ± 45.7	0.18
Cholesterol [mg/dL]	166.8 ± 55.0	143.6 ± 40.5	0.01
Triglycerides [mg/dL]	123.6 ± 42.0	94.6 ± 96.2	0.01
Albumin [g/dL]	3.40 ± 0.70	3.00 ± 0.50	0.02
AFP [ng/mL]	37.8 ± 1294.2	7.91 ± 1036.2	0.05
HOMA-IR	2.82 ± 6.40	7.06 ± 8.80	< 0.001

Serum vaspin level was significantly higher among patients with BMI > 30 kg/m^2^ compared to subjects with BMI ≤ 30 kg/m^2^ (0.23±0.60 vs 0.12±1.16, p = 0.04), whereas serum visfatin concentration did not differ between these groups (p = 0.32).

Obesity and IR are core components of the metabolic syndrome and also significant risk factors for the development of HCC. The detailed mechanism linking related metabolic abnormalities to their complications has been trying to be revealed by some studies. It has been reported that abnormalities in the circulating levels of visfatin and its gene expression are related to BMI and markers of insulin sensitivity in metabolic syndrome patients [10,23]. In this study, we evaluated the potential value of serum visfatin and vaspin in HCC patients. Obtained results indicated that serum visfatin and vaspin levels were significantly elevated in patients with HCC when compared to healthy volunteers. In addition, serum visfatin levels were significantly higher in patients with higher IR.

Potential role of visfatin in the development of HCCs as a complication of metabolic abnormalities has been shown in some studies. First study by Ninomiya et al. suggested that visfatin is one of the key adipokines that links obesity and the progression of HCC [15]. It revealed potential role of visfatin, which actually might act as a growth factor in HCC cells, which could be implicated in to increase of HCC cells proliferation.

Several studies suggest that the role of visfatin in the development of the previously mentioned cancer has been connected to several possible mechanisms. First, visfatin might be a proinflammatory molecule produced by adipose tissue macrophages that could block macrophage apoptosis induced by a number of endoplasmic reticulum (ER) stressors (the ER stress is a process associated with obesity and obesity related diseases). Visfatin rapidly upregulates IL-6 protein secretion, which activates prosurvival signal transducer and activator of transcription 3 (STAT3) and thus might play a role in obesity-associated diseases, such as inflammation or tumorigenesis [24]. Additionally, up-regulation of visfatin is directly associated with increase of Sirt6, which acts post-transcriptionally in the upregulation of TNF-α [25]. Furthermore, in an apoptotic state visfatin induces the reduction of reactive oxygen metabolites through increased activity of antioxidative enzymes superoxide dismutase (SOD), catalase (CAT), and glutathione peroxidase (GSHPx). This cell protection from cytotoxic damage of reactive oxygen species was demonstrated in cultured human melanoma cells (Me45), where visfatin treatment increased activity of the antioxidative enzymes [26]. Besides, Adya, et al revealed Nampt/PBEF/visfatin induced dose- and time-dependent proliferation and capillary-like tube formation of human endothelial cells. This angiogenic effects is promoted by up-regulation of VEGF and MMP expression. Main role in visfatin-induced VEGF/MMP up-regulation and endothelial angiogenesis plays VEGF/VEGF receptor 2 (VEGFR2) signaling, phosphorylation of ERK 1/2, and PI3K/Akt pathways [12, 27].

Tsai et al. showed that a higher risk of HCC in the ethnically Chinese HBV and/or HCV carriers was related to an elevated plasma visfatin level, and that there was possibly a close connection between visfatin and chronic inflammation and the development of HCC [28]. In our study we have not shown the same findings. Serum concentration of visfatin was not significantly increased in HCC patients with viral etiology compared to those with non-viral etiology. The study performed by Tsai et al., included only subjects with HBV and HCV infection, while our study included subjects with diversified etiology of HCC, which might produce potential bias for our results.

Sun et al’s [29] analysis has shown that HCC patients with higher levels of serum visfatin had significantly higher-grade malignancy, and poor overall survival. They proved that serum visfatin concentration in HCC patients was positively correlated with AFP and IL-6 and was also associated with tumor size and tumor node metastasis stage. In our study there was no difference in visfatin concentrations when compared groups of patients with HCC according to BCLC classification. Visfatin concentration also did not differ between patients with various CTP. However, the study performed by Sun et al. involved only participants with BMI <25 kg/m^2^, which may be the main reason for the inconsistent results between the studies, as visfatin is upregulated during adipocyte differentiation. Finally, their study focused only on the patients with HBV infection, while we included subjects with diversified etiology of HCC.

Study performed by Fukuhara, et al indicated that visfatin lowers glucose serum levels and decreases IR [30]. However, Nourbakhsh et al [31] and most of studies proved [32,33] that visfatin had a strong positive correlation with fasting plasma glucose, insulin, and HOMA-IR. Our study seems to confirm those findings as serum visfatin level was found to be higher in patients with higher HOMA-IR value.

In the study performed by Jung et al., vaspin has been found to play antiatherogenic role. Vaspin activated dimethylarginine dimethylaminohydrolase II (DDAH) and decreased the levels of asymmetric dimethylarginine (ADMA), an endogenous inhibitor of all types of nitric oxide synthases (NOSs). High levels of ADMA attenuated endothelium-dependent vasodilation in humans. DDAH is an enzyme that is involved in metabolism of ADMA to citrulline and dimethylamine, plays a crucial role as a modulator of intracellular and extracellular ADMA concentrations and has a potential to regulate NO bioavailability through that modulation. Some recent evidences have shown that DDAH activity is decreased in oxidative stress, permitting ADMA to accumulate. Emphasizing the beneficial effect of vaspin on oxidative stress and IR, it may exert protective effect against platelets hyperactivity [34,35]. Unfortunately, in our study, no significant difference was observed in vaspin serum concentration between groups of patients with different platelets count.

Several human research studies focused on the potential correlation between serum vaspin levels and obesity or T2DM [7]. However, to the best of our knowledge, there are only two recent prospective studies showed the link between vaspin and specific cancers [36,37]. There are reports of higher levels of vaspin in colorectal cancer [36], independent of measures of obesity, but the exact mechanism underlying vaspin in relation to cancer growth remained to be elucidated. The study by Cymbaluk-Płoska et al. showed that lower concentrations of vaspin may indicate the presence of endometrial cancer. It was underlying the protective role of vaspin, which may improve tissue insulin understanding and inhibit inflammatory processes in vascular endothelium [34,37]. Neither study has successfully explained the mechanism of vaspin to the respective cancer. Furthermore, the results from one study contradict the other. Our study showed for the first time the positive association between elevated vaspin level and HCC with significantly up-regulated vaspin levels in patients with liver cancer compared to healthy controls.

Some studies showed that vaspin level may be upregulated in a compensatory mechanism associated with obesity and IR [8]. Our study partially confirmed that findings, since elevated vaspin concentration was associated with higher BMI, but was not significantly correlated with HOMA-IR. Results of our study showed that higher vaspin level was independent of IR. However, it has to be mentioned that all HCC patients were more or less insulin resistant. Insulin resistance was probably provoked by cancer, and observed up-regulation of vaspin serum levels could be a compensatory mechanism. Nevertheless, there was also no difference in vaspin serum concentration when compared HCC patients with high and lower values of HOMA-IR.

Our study also confirmed the presence of sexual dimorphism in the concentration of serum vaspin level, with higher concentration in females. Our study is in agreement with the study by Seeger et al., which revealed sex to be a predictor of circulating vaspin [38].

Vaspin has been found to suppress the expression proinflammatory and profibrogenic agents such as leptin, resistin and TNF-α [39]. Linking between vaspin and fibrosis was searched in study performed by Kukla et al in CHC patients. Intriguing results showed suppression of the serum vaspin level in non-obese CHC patients without significant fibrosis with subsequent evident increase in those with significant or advanced fibrosis and serum vaspin levels were found to be positively associated with fibrosis stage [20]. Another recent study confirmed that the serum vaspin level raised with fibrosis progression, probably as a compensatory mechanism against increasing IR, which was positively related to fibrosis stage [39]. Our study showed that serum concentration of vaspin was significantly higher in HCC patients with viral etiology compared to those with non-viral etiology of HCC. Moreover, the elevation of serum vaspin level was even more evident in group of HCV positive patients. There is well known that HCV promote IR on one side by influence on insulin signaling pathway, inhibiting signal transduction and on the other side by liver disease progression [40]. These observations explain obtained results and confirm protective and compensatory role of vaspin against IR.

The findings presented in this study should be interpreted considering limitations of using cross-sectional design of study. There were some other, unavoidable limitations. First, our study included relatively small groups of patients with cirrhosis of different origin, and did not include end stage of HCC. Pointing to the influence of metabolic abnormalities on adipokines levels, the comparison of analyzed adipokines between patients with chronic liver diseases of metabolic origin with those with other causes of chronic liver disease such as alcohol abuse or viral infection would shed some light on better understanding of exact role of analyzed adipokines in cirrhosis and HCC pathogenesis. However, it requires larger group of patients. Second, our study did not assess the amount of adipose tissue and mass of muscles which can influence some serum adipokines levels. A number of authors have pointed out that reliance on BMI as a sole marker of obesity is the serious limitation of studies on relationship between several disorders and obesity. They indicate a poor linear relationship between BMI and total body fat and also suggested that body fat distribution would be more clinically significant than overall obesity. Third, our study, showed considerable variability in serum concentrations of visfatin and vaspin, but did not assess expression of analyzed adipokines in tumor and surrounding liver tissue and adipose tissue.

## Conclusions

In conclusion, our study revealed, for the first time, serum vaspin to be significantly increased in HCC patients independently of cancer ethiology compared to healthy controls. Additionally, our study showed serum visfatin to be elevated in the presence of HCC. Vaspin up-regulation can be a compensatory mechanism against IR in HCC patients, especially in those with HCV infection. Serum visfatin and vaspin, although up-regulated, seem not to be associated with cancer grade and cirrhosis severity. However, a role of these factors, especially vaspin, in carcinogenesis, particularly of HCC requires additional research.

The research was not subsidized.

## Supporting information

S1 File Statystyka. (DOCX)

## Abbreviations

AFP: Alpha-Fetoprotein
ALT: Alanine Transaminase
AST: Aspartate Transaminase
BMI: Body Mass Index
CA 19.9: Carbohydrate Antigen 19.9
CEA: Carcinoembryionic Antigen
FA: Fatty Acids
GGTP: Gamma-Glutamyl-Transferase
HDL: High-Density Lipoprotein
HGB: Hemoglobin
WBC: White Blood Cells
PLT: Platelets Count

## References

[1] Altekruse SF, Devesa SS, Dickie LA, McGlynn KA, Kleiner DE. Histological classification of liver and intrahepatic bile duct cancers in SEER registries. J. Regist. Manag. 2011, 38: 201–205. PMC4148005 23270094

[2] Gomaa AI, Khan SA, Toledano MB, Waked I, Taylor-Robinson SD. Hepatocellular carcinoma: epidemiology, risk factors and pathogenesis. World. J. Gastroenterol. 2008, 14: 4300–4308. 10.3748/wjg.14.4300 18666317 PMC2731180

[3] Said A, Ghufran A. Epidemic of non-alcoholic fatty liver disease and hepatocellular carcinoma. World J. Clin. Oncol. 2017, 8: 429–436. 10.5306/wjco.v8.i6.429 29291167 PMC5740098

[4] Derra A, Bator M, Menżyk T, Kukla M. Underrated enemy–from nonalcoholic fatty liver disease to cancers of the gastrointestinal tract. Clin. Exp. Hepatol. 2018, 4: 55–71. 10.5114/ceh.2018.75955 29904722 PMC6000748

[5] Cholankeril G, Patel R, Khurana S, Satapathy SK. Hepatocellular carcinoma in non-alcoholic steatohepatitis: Current knowledge and implications for management. World J Hepatol. 2017, 9: 533–543. 10.4254/wjh.v9.i11.533 28469809 PMC5395802

[6] Kukla M. Angiogenesis: a phenomenon which aggravates chronic liver disease progression. Hepatol Int. 2013, 7: 4 10.1007/s12072-012-9391-2 26201617

[7] Kukla M, Zwirska-Korczala K, Gabriel A. Chemerin, vaspin and insulin resistance in chronic hepatitis C. J. Viral. Hepat. 2010, 17: 661–7. 10.1111/j.1365-2893.2009.01224.x 20002564

[8] Kukla M, Mazur W, Bułdak RJ, Zwirska-Korczala K. Potential role of leptin, adiponectin and three novel adipokines-visfatin, chemerin and vaspin-in chronic hepatitis. Mol. Med. 2011, 17:1397–410. 10.2119/molmed.2010.00105 21738955 PMC3321801

[9] Kukla M, Zwirska-Korczala K, Hartleb M, Waluga M, Chwist A, Kajor M, et al Serum chemerin and vaspin in non-alcoholic fatty liver disease. Scand. J. Gastroenterol. 2010, 45: 235–242. 10.3109/00365520903443852 20095887

[10] Stastny J, Bienertova-Vasku J, Vasku A. Visfatin and its role in obesity development. Diabetes Metab. Syndr. 2012, 6: 120–4. 10.1016/j.dsx.2012.08.011 23153983

[11] Bi TQ, Che XM. Nampt/PBEF/visfatin and cancer. Cancer Biol. Ther. 2010, 10: 119–25. 10.4161/cbt.10.2.12581 20647743

[12] Raghu A, Bee KT, Anu P, Jing Ch, Harpal SR. Visfatin induces human endothelial VEGF and MMP-2/9 production via MAPK and PI3K/Akt signalling pathways: novel insights into visfatin-induced angiogenesis. Cardiovascular Research. 2008, 78: 356–365. 10.1093/cvr/cvm111 18093986

[13] Moschen AR, Kaser A, Enrich B, Mosheimer B, Theurl M, Niederegger H, et al, Visfatin an adipocytokine with proinflammatory and immunomodulating properties. J. Immunol. 2007, 178: 1748–58. 10.4049/jimmunol.178.3.1748 17237424

[14] Booth A, Magnuson A, Fouts J, Foster M. Adipose tissue, obesity and adipokines: role in cancer promotion. Horm. Mol. Biol. Clin. Investig. 2015, 21: 57–74. 10.1515/hmbci-2014-0037 25781552

[15] Ninomiya S, Shimizu M, Imai K. Possible role of visfatin in hepatoma progression and the effects of branched-chain amino acids on visfatin-induced proliferation in human hepatoma cells. Cancer Prev. Res. (Phila). 2011, 4: 2092–2100. 10.1158/1940-6207.CAPR-11-0340 21952585

[16] Akbal E, Koçak E, Taş A, Yüksel E, Köklü S. Visfatin levels in nonalcoholic fatty liver disease. J. Clin. Lab. Anal. 2012, 26: 115–9. 10.1002/jcla.21491 22467327 PMC6807497

[17] Hida K, Wada J, Eguchi J, et al Visceral adipose tissue-derived serine protease inhibitor: a unique insulin-sensitizing adipocytokine in obesity. USA Proc. Natl. Acad. Sci. 2005, 102: 10610–5. 10.1073/pnas.0504703102 PMC1180799 16030142

[18] Klöting N, Berndt J, Kralisch S. et al Vaspin gene expression in human adipose tissue: association with obesity and type 2 diabetes. Biochem. Biophys. Res. Commun. 2006, 339: 430–6. 10.1016/j.bbrc.2005.11.039 16298335

[19] Rabe K, Lehrke M, Parhofer KG. et al Adipokines and insulin resistance. Mol. Med. 2008, 14: 741–751. 10.2119/2008-00058.Rabe 19009016 PMC2582855

[20] Kukla M, Waluga M, Sawczyn T et al Serum vaspin may be a good indicator of fibrosis in chronic hepatitis C and is not altered by antiviral therapy. Pol. J. Pathol. 2012, 63: 213–220. 10.5114/pjp.2012.32767. 23359189

[21] WHO. Physical Status: The Use and Interpretation of Anthropometry: Report of a World Health Organization (WHO) Expert Committee. 1995, Geneva, World Health Organization. 8594834

[22] World Health Organization. Definition and Diagnosis of Diabetes Mellitus and Intermediate Hyperglycemia: Report of a WHO/IDF Consultation. 2006, Geneva, World Health Organization.

[23] Filippatos TD, Derdemezis CS, Kiortsis DN, Tselepis AD, Elisaf MS. Increased plasma levels of visfatin/pre-B cell colony-enhancing factor in obese and overweight patients with metabolic syndrome. J. Endocrinol. Invest. 2007, 30: 323–6. 10.1007/bf03346300 17556870

[24] Li Y, Zhang Y, Dorweiler B, Cui D, Wang T, Woo CW et al Extracellular Nampt promotes macrophage survival via a nonenzymatic interleukin-6/STAT3 signaling mechanism. J. Biol. Chem. 2008, 283: 34833–43. 10.1074/jbc.M805866200 18945671 PMC2596403

[25] Van Gool F, Galli M, Gueydan C, Kruys V, Prevot PP, Bedalov A, et al Intracellular NAD levels regulate tumor necrosis factor protein synthesis in a sirtuin-dependent manner. Nat. Med. 2009, 15: 206–10. 10.1038/nm.1906 19151729 PMC2845476

[26] Buldak RJ, Buldak L, Polaniak R, Kukla M, Birkner E, Kubina R, et al Visfatin affects redox adaptative responses and proliferation in Me45 human malignant melanoma cells: an in vitro study. Oncol. Rep. 2013, 29: 771–8. 10.3892/or.2012.2175 23232726

[27] Adya R, Tan BK, Punn A, Chen J, Randeva HS. Visfatin induces human endothelial VEGF and MMP-2/9 production via MAPK and PI3K/Akt signalling pathways: novel insights into visfatin-induced angiogenesis. Cardiovasc. Res. 2008, 78: 356–365. 10.1093/cvr/cvm111 18093986

[28] Tsai IT, Wang CP, Yu TH, Lu YC, Lin CW, Lu LF et al Circulating visfatin level is associated with hepatocellular carcinoma in chronic hepatitis B or C virus infection. Cytokine. 2017, 90: 54–59. 10.1016/j.cyto.2016.10.007 27770715

[29] Sun Y, Zhu S, Wu Z, Huang Y, Liu C, Tang S, et al Elevated serum visfatin levels are associated with poor prognosis of hepatocellular carcinoma. Oncotarget. 2017, 8: 23427–23435. 10.18632/oncotarget.15080 28178643 PMC5410315

[30] Fukuhara A, Matsuda M, Nishizawa M, et al Visfatin: a protein secreted by visceral fat that mimics the effects of insulin. Science. 2005, 307: 426–430. 10.1126/science.1097243 15604363

[31] Nourbakhsh M, Nourbakhsh M, Gholinejad Z, Razzaghy-Azar M. Visfatin in obese children and adolescents and its association with insulin resistance and metabolic syndrome. Scand. J. Clin. Lab. Invest. 2015, 75: 183–8. 10.3109/00365513.2014.1003594 25723377

[32] Brown JE, Onyango DJ, Ramanjaneya M, Conner AC, Patel ST, Dunmore SJ, et al Visfatin regulates insulin secretion, insulin receptor signalling and mRNA expression of diabetes-related genes in mouse pancreatic β-cells. J. Mol. Endocrinol. 2010, 44: 171–178. 10.1677/JME-09-0071 19906834

[33] Kabir F, Jahan FA, Khan I, Faruque MO, Hassan Z, Ali L. Increased concentration of circulating visfatin associates with post‐challenged hyperglycaemia and insulin resistance in IGT subjects. J. Taibah. Univ. Med. Sci. 2015, 10: 481–487. 10.1016/j.jtumed.2014.12.007.

[34] Jung CH, Lee WJ, Hwang JY, Lee MJ, Seol SMs et al Vaspin Increases Nitric Oxide Bioavailability through the Reduction of Asymmetric Dimethylarginine in Vascular Endothelial Cells. PLoS ONE. 2012, 7, e52346 10.1371/journal.pone.0052346 23284999 PMC3532208

[35] Kukla M, Waluga M, Żorniak M, Berdowska A, Wosiewicz P, Sawczyn T, et al Serum omentin and vaspin levels in cirrhotic patients with and without portal vein thrombosis. World. J. Gastroenterol. 2017, 23: 2613–2624. 10.3748/wjg.v23.i14.2613 28465646 PMC5394525

[36] Fazeli MS, Dashti H, Akbarzadeh S, Assadi M, Aminian A, Keramati MR, et al Circulating levels of novel adipocytokines in patients with colorectal cancer. Cytokine. 2013, 62: 81–5. 10.1016/j.cyto.2013.02.012 23474107

[37] Cymbaluk-Płoska A, Chudecka-Głaz A, Jagodzińska A, Pius-Sadowska E, Sompolska-Rzechuła A, Machaliński B, et al Evaluation of biologically active substances promoting the development of or protecting against endometrial cancer. Onco. Targets Ther. 2018, 11: 1363–1372. 10.2147/OTT.S155942 29559794 PMC5856062

[38] Seeger J, Ziegelmeier M, Bachmann A, Lössner U, Kratzsch J, Blüher M. Serum levels of the adipokine vaspin in relation to metabolic and renal parameters. J. Clin. Endocrinol. Metab. 2008, 93:247–51. 10.1210/jc.2007-1853 17956947

[39] Rabe K, Lehrke M, Parhofer KG, Broedl UC. Adipokines and insulin resistance. Mol. Med. 2008, 14: 741–751. 10.2119/2008-00058.Rabe 19009016 PMC2582855

[40] Kukla M, Piotrowski D, Waluga M, Hartleb M. Insulin resistance and its consequences in chronic hepatitis C. Clin. Exp. Hepatol. 2015, 1: 17–29. 10.5114/ceh.2015.51375 28856251 PMC5421163

