# Supplementary material for: Serum visfatin and vaspin levels in hepatocellular carcinoma (HCC)
Source: PLoS One. 2020 Jan 14;15(1):e0227459. doi: 10.1371/journal.pone.0227459 (PMC6959555; doi:10.1371/journal.pone.0227459)
Supplement: S1 File — (DOCX) [file pone.0227459.s001.docx]

Statystyka

Ogólna: ogólna statystyka badanej grup z marskością i HCC

|  | \| N \| \| --- \| | \| Średnia \| \| --- \| | \| Mediana \| \| --- \| | \| Minimum \| \| --- \| | \| Maksimum \| \| --- \| | \| Kwartyl. Rozstęp \| \| --- \| | \| Odch.std \| \| --- \| | Norm |
| --- | --- | --- | --- | --- | --- | --- | --- | --- | --- | --- | --- | --- | --- | --- | --- |
| \| Waspina [ng/mL] \| \| --- \| | 66 | 0,52 | 0,18 | 0,029 | 5,23 | 0,47 | 0,93 | N |
| \| Wisfatyna [ng/mL] \| \| --- \| | 66 | 4,26 | 4,50 | 0,517 | 8,78 | 2,74 | 1,79 | T |
| \| Iryzyna [µg/mL] \| \| --- \| | 69 | 2,73 | 2,52 | 0,411 | 4,75 | 1,81 | 1,14 | N |
| \| ESM-1 [ng/mL] \| \| --- \| | 67 | 23,09 | 20,55 | 16,120 | 60,04 | 4,62 | 8,40 | N |
| \| Betatrofina [ng/mL] \| \| --- \| | 65 | 33,03 | 33,72 | 10,895 | 49,96 | 24,69 | 13,38 | N |
| \| Insulina [ng/mL] \| \| --- \| | 68 | 1,17 | 0,89 | 0,231 | 3,94 | 0,85 | 0,91 | N |
| \| Insulina [mIU/mL] \| \| --- \| | 69 | 26,75 | 20,19 | 5,223 | 90,92 | 19,60 | 20,92 | N |
| \| Wiek( lata) \| \| --- \| | 69 | 60,20 | 59,00 | 20,000 | 88,00 | 12,00 | 12,11 | N |
| \| WBC 106/ul \| \| --- \| | 68 | 5,00 | 4,30 | 1,780 | 10,35 | 3,85 | 2,46 | N |
| \| HGB mg/dl \| \| --- \| | 69 | 11,80 | 12,20 | 3,160 | 16,50 | 3,40 | 2,45 | N |
| \| PLT 106/ul \| \| --- \| | 68 | 114,69 | 88,50 | 18,000 | 392,00 | 75,50 | 85,03 | N |
| \| ALT IU/L \| \| --- \| | 69 | 60,68 | 45,00 | 14,000 | 303,00 | 52,00 | 48,52 | N |
| \| AST IU/L \| \| --- \| | 69 | 82,94 | 63,00 | 16,000 | 409,00 | 65,00 | 65,08 | N |
| \| FA IU/L \| \| --- \| | 69 | 143,04 | 110,00 | 38,000 | 476,00 | 72,00 | 89,41 | N |
| \| GGTP Iu/L \| \| --- \| | 69 | 149,87 | 91,00 | 21,000 | 1243,00 | 105,00 | 194,60 | N |
| \| Glukoza mg/dl \| \| --- \| | 69 | 120,62 | 106,80 | 56,700 | 281,00 | 43,80 | 43,92 | N |
| \| Mocznik mg/dl \| \| --- \| | 69 | 41,72 | 35,30 | 14,700 | 229,30 | 19,00 | 30,46 | N |
| \| Kreatynina mg/dL \| \| --- \| | 69 | 1,38 | 0,79 | 0,490 | 37,40 | 0,22 | 4,41 | N |
| \| Bilirubina mg/dl \| \| --- \| | 69 | 4,36 | 1,58 | 0,430 | 152,10 | 1,67 | 18,13 | N |
| \| Cholesterol mg/dl \| \| --- \| | 69 | 153,14 | 147,00 | 2,020 | 374,00 | 45,10 | 50,11 | N |
| \| Triglicerydy mg/dl \| \| --- \| | 69 | 116,69 | 105,00 | 40,000 | 676,10 | 56,70 | 78,64 | N |
| \| HDL mg/dl \| \| --- \| | 69 | 42,16 | 37,40 | 7,800 | 235,80 | 17,80 | 31,04 | N |
| \| Pt % \| \| --- \| | 69 | 74,35 | 75,00 | 40,000 | 102,80 | 18,00 | 14,11 | T |
| \| Białko całkowite g/dl \| \| --- \| | 69 | 7,14 | 7,10 | 4,300 | 9,80 | 1,00 | 0,94 | T |
| \| Albumina g/dl \| \| --- \| | 69 | 3,22 | 3,10 | 1,900 | 4,60 | 0,70 | 0,59 | T |
| \| APF ng/ml \| \| --- \| | 69 | 362,26 | 15,96 | 0,740 | 5845,00 | 39,14 | 1145,00 | N |
| \| CEA ng/ml \| \| --- \| | 69 | 3,52 | 2,86 | 0,165 | 18,92 | 2,46 | 2,91 | N |
| \| CA 19,9 U/ml \| \| --- \| | 69 | 33,65 | 15,06 | 2,000 | 728,70 | 20,49 | 89,29 | N |
| \| BMI \| \| --- \| | 69 | 29,59 | 29,00 | 22,000 | 45,00 | 6,00 | 4,49 | N |
| \| Obwód pasa cm \| \| --- \| | 69 | 102,74 | 102,00 | 70,000 | 139,00 | 15,00 | 12,56 | T |
| \| HOMA-IR \| \| --- \| | 69 | 8,37 | 5,47 | 1,377 | 48,21 | 7,33 | 8,10 | N |

(NORM – ROZKŁAD T – ROZKŁAD NORMALNY, N-ROZKŁAD ODBIEGAJĄCY OD NORMALNEGO)

| Klasa | Tabela liczności: Płeć (STAT) | |
| --- | --- | --- |
|  | \| Liczba \| \| --- \| | \| Procent \| \| --- \| |
| \| m \| \| --- \| | 54 | 78,26087 |
| \| k \| \| --- \| | 15 | 21,73913 |

| Klasa | Tabela liczności: HCV (STAT) | |
| --- | --- | --- |
|  | \| Liczba \| \| --- \| | \| Procent \| \| --- \| |
| \| Tak \| \| --- \| | 35 | 50,72464 |
| \| Nie \| \| --- \| | 34 | 49,27536 |
| Klasa | Tabela liczności: Hbc (STAT) | |
|  | \| Liczba \| \| --- \| | \| Procent \| \| --- \| |
| \| Tak \| \| --- \| | 29 | 42,02899 |
| \| Nie \| \| --- \| | 40 | 57,97101 |

| Klasa | Tabela liczności: HbsAg (STAT) | |
| --- | --- | --- |
|  | \| Liczba \| \| --- \| | \| Procent \| \| --- \| |
| \| Nie \| \| --- \| | 61 | 88,40580 |
| \| Tak \| \| --- \| | 8 | 11,59420 |

| Klasa | Tabela liczności: Wirusy (STAT) | |
| --- | --- | --- |
|  | \| Liczba \| \| --- \| | \| Procent \| \| --- \| |
| \| Tak \| \| --- \| | 43 | 62,31884 |
| \| Nie \| \| --- \| | 26 | 37,68116 |

| Klasa | Tabela liczności: Cukrzyca t,II (STAT) | |
| --- | --- | --- |
|  | \| Liczba \| \| --- \| | \| Procent \| \| --- \| |
| \| Tak \| \| --- \| | 27 | 39,13043 |
| \| Nie \| \| --- \| | 42 | 60,86957 |

| Klasa | Tabela liczności: Nadciśnienie tętnicze (STAT) | |
| --- | --- | --- |
|  | \| Liczba \| \| --- \| | \| Procent \| \| --- \| |
| \| Tak \| \| --- \| | 20 | 28,98551 |
| \| Nie \| \| --- \| | 49 | 71,01449 |

| Klasa | Tabela liczności: HCC (STAT) | |
| --- | --- | --- |
|  | \| Liczba \| \| --- \| | \| Procent \| \| --- \| |
| \| Tak \| \| --- \| | 45 | 65,21739 |
| \| Nie \| \| --- \| | 24 | 34,78261 |

| Klasa | Tabela liczności: BCLC (STAT) | |
| --- | --- | --- |
|  | \| Liczba \| \| --- \| | \| Procent \| \| --- \| |
| \| A \| \| --- \| | 12 | 17,39130 |
| \| B \| \| --- \| | 20 | 28,98551 |
| \| C \| \| --- \| | 16 | 23,18841 |
| \| Braki \| \| --- \| | 21 | 30,43478 |

| Klasa | Tabela liczności: Marskość wątroby (STAT) | |
| --- | --- | --- |
|  | \| Liczba \| \| --- \| | \| Procent \| \| --- \| |
| \| Tak \| \| --- \| | 68 | 98,55072 |
| \| Nie \| \| --- \| | 1 | 1,44928 |

| Klasa | Tabela liczności: CHP (STAT) skala Child-Pugh stopnie A, B, C | |
| --- | --- | --- |
|  | \| Liczba \| \| --- \| | \| Procent \| \| --- \| |
| \| A \| \| --- \| | 36 | 52,17391 |
| \| B \| \| --- \| | 27 | 39,13043 |
| \| C \| \| --- \| | 6 | 8,69565 |

| Klasa | Tabela liczności: CHP pkt (STAT) skala Child-Pugh punkty | |
| --- | --- | --- |
|  | \| Liczba \| \| --- \| | \| Procent \| \| --- \| |
| \| 5 \| \| --- \| | 15 | 21,73913 |
| \| 6 \| \| --- \| | 21 | 30,43478 |
| \| 7 \| \| --- \| | 9 | 13,04348 |
| \| 8 \| \| --- \| | 12 | 17,39130 |
| \| 9 \| \| --- \| | 6 | 8,69565 |
| \| 10 \| \| --- \| | 4 | 5,79710 |
| \| 11 \| \| --- \| | 2 | 2,89855 |

| Klasa | Tabela liczności: HOMA-IR Grupy (STAT) | |
| --- | --- | --- |
|  | \| Liczba \| \| --- \| | \| Procent \| \| --- \| |
| \| <4 \| \| --- \| | 25 | 36,23188 |
| \| >4 \| \| --- \| | 44 | 63,76812 |

| Klasa | Tabela liczności: PLT Grupy (STAT) płytki >100 000 i poniżej | |
| --- | --- | --- |
|  | \| Liczba \| \| --- \| | \| Procent \| \| --- \| |
| \| >100 \| \| --- \| | 28 | 40,57971 |
| \| <100 \| \| --- \| | 41 | 59,42029 |

| Klasa | Tabela liczności: Glukoza grupy (STAT) | |
| --- | --- | --- |
|  | \| Liczba \| \| --- \| | \| Procent \| \| --- \| |
| \| <100 \| \| --- \| | 29 | 42,02899 |
| \| >100 \| \| --- \| | 40 | 57,97101 |

| Klasa | Tabela liczności: BMI grupy (STAT) | |
| --- | --- | --- |
|  | \| Liczba \| \| --- \| | \| Procent \| \| --- \| |
| \| >30 \| \| --- \| | 30 | 43,47826 |
| \| <30 \| \| --- \| | 39 | 56,52174 |

Podział Badana vs Kontrolna (ludzie zupełnie zdrowi w podobnym wieku)

| Zmienna | Grupy=Badana | | | | | | |
| --- | --- | --- | --- | --- | --- | --- | --- |
|  | \| N \| \| --- \| | \| Średnia \| \| --- \| | \| Mediana \| \| --- \| | \| Minimum \| \| --- \| | \| Maksimum \| \| --- \| | \| Kwartyl. Rozstęp \| \| --- \| | \| Odch.std \| \| --- \| |
| \| Waspina [ng/mL] \| \| --- \| | 66 | 0,51561 | 0,17912 | 0,02895 | 5,23057 | 0,47066 | 0,93102 |
| \| Wisfatyna [ng/mL] \| \| --- \| | 66 | 4,25647 | 4,49745 | 0,51718 | 8,78366 | 2,74327 | 1,79379 |
| \| Iryzyna [µg/mL] \| \| --- \| | 69 | 2,72697 | 2,52268 | 0,41129 | 4,75315 | 1,81286 | 1,13605 |
| \| ESM-1 [ng/mL] \| \| --- \| | 67 | 23,09391 | 20,55313 | 16,11979 | 60,03646 | 4,61667 | 8,40454 |
| \| Betatrofina [ng/mL] \| \| --- \| | 65 | 33,02738 | 33,71756 | 10,89534 | 49,96320 | 24,68975 | 13,37853 |
| \| Insulina [ng/mL] \| \| --- \| | 68 | 1,17200 | 0,89104 | 0,23126 | 3,93696 | 0,85047 | 0,90555 |
| \| Insulina [mIU/mL] \| \| --- \| | 68 | 27,06688 | 20,57825 | 5,34092 | 90,92294 | 19,64129 | 20,91344 |

| Zmienna | Grupy=Kontrolna | | | | | | |
| --- | --- | --- | --- | --- | --- | --- | --- |
|  | \| Nważnych \| \| --- \| | \| Średnia \| \| --- \| | \| Mediana \| \| --- \| | \| Minimum \| \| --- \| | \| Maksimum \| \| --- \| | \| Kwartyl. Rozstęp \| \| --- \| | \| Odch.std \| \| --- \| |
| \| Waspina [ng/mL] \| \| --- \| | 10 | 0,11390 | 0,10180 | 0,02220 | 0,27141 | 0,108842 | 0,078588 |
| \| Wisfatyna [ng/mL] \| \| --- \| | 10 | 3,06007 | 2,62210 | 1,32173 | 5,82823 | 2,025534 | 1,471186 |
| \| Iryzyna [µg/mL] \| \| --- \| | 10 | 3,72894 | 4,46514 | 1,65481 | 4,81977 | 2,599386 | 1,339922 |
| \| ESM-1 [ng/mL] \| \| --- \| | 10 | 25,07333 | 24,54167 | 21,51667 | 30,83333 | 4,033333 | 2,763398 |
| \| Betatrofina [ng/mL] \| \| --- \| | 10 | 12,35843 | 12,33189 | 10,24323 | 17,27517 | 1,852448 | 1,999511 |
| \| Insulina [ng/mL] \| \| --- \| | 10 | 0,49621 | 0,44335 | 0,32862 | 0,73182 | 0,256401 | 0,149471 |
| \| Insulina [mIU/mL] \| \| --- \| | 10 | 11,45988 | 10,23914 | 7,58947 | 16,90119 | 5,921512 | 3,451986 |

|  | Badana | Kontrolna | Test | p |
| --- | --- | --- | --- | --- |
| \| Waspina [ng/mL] \| \| --- \| | N | T | U Manna Whitneya | 0,0509923585 |
| \| Wisfatyna [ng/mL] \| \| --- \| | T | T | T Studenta | 0,0485317115 |
| \| Iryzyna [µg/mL] \| \| --- \| | N | N | U Manna Whitneya | 0,0198258241 |
| \| ESM-1 [ng/mL] \| \| --- \| | N | T | U Manna Whitneya | 0,00336621869 |
| \| Betatrofina [ng/mL] \| \| --- \| | N | N | U Manna Whitneya | 0,00000743555 |
| \| Insulina [ng/mL] \| \| --- \| | N | T | U Manna Whitneya | 0,00247367061 |
| \| Insulina [mIU/mL] \| \| --- \| | N | T | U Manna Whitneya | 0,00247367061 |

(T- rozkład normalny, N- odbiega od normalnego – Shapiro-Wilk)


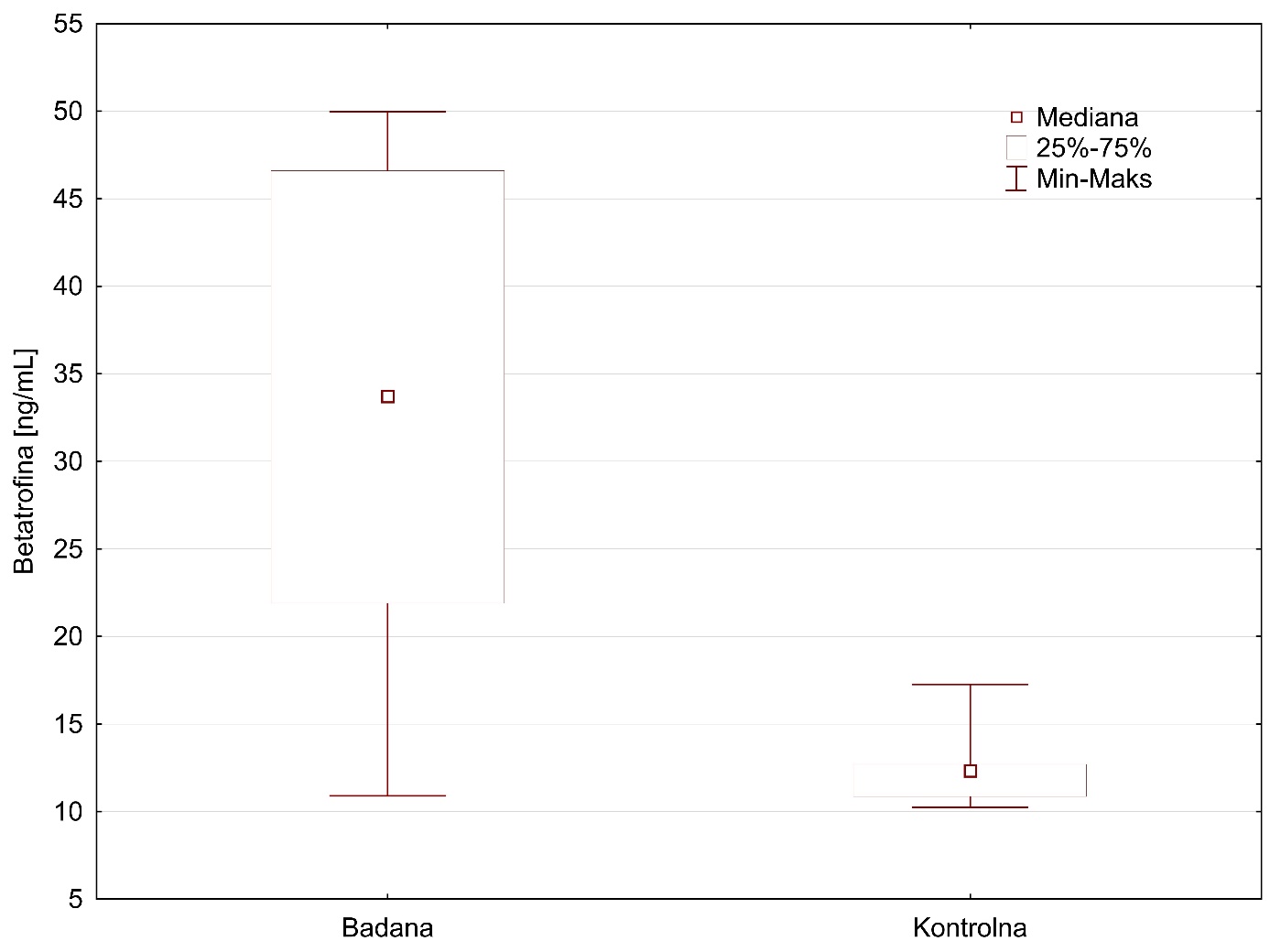

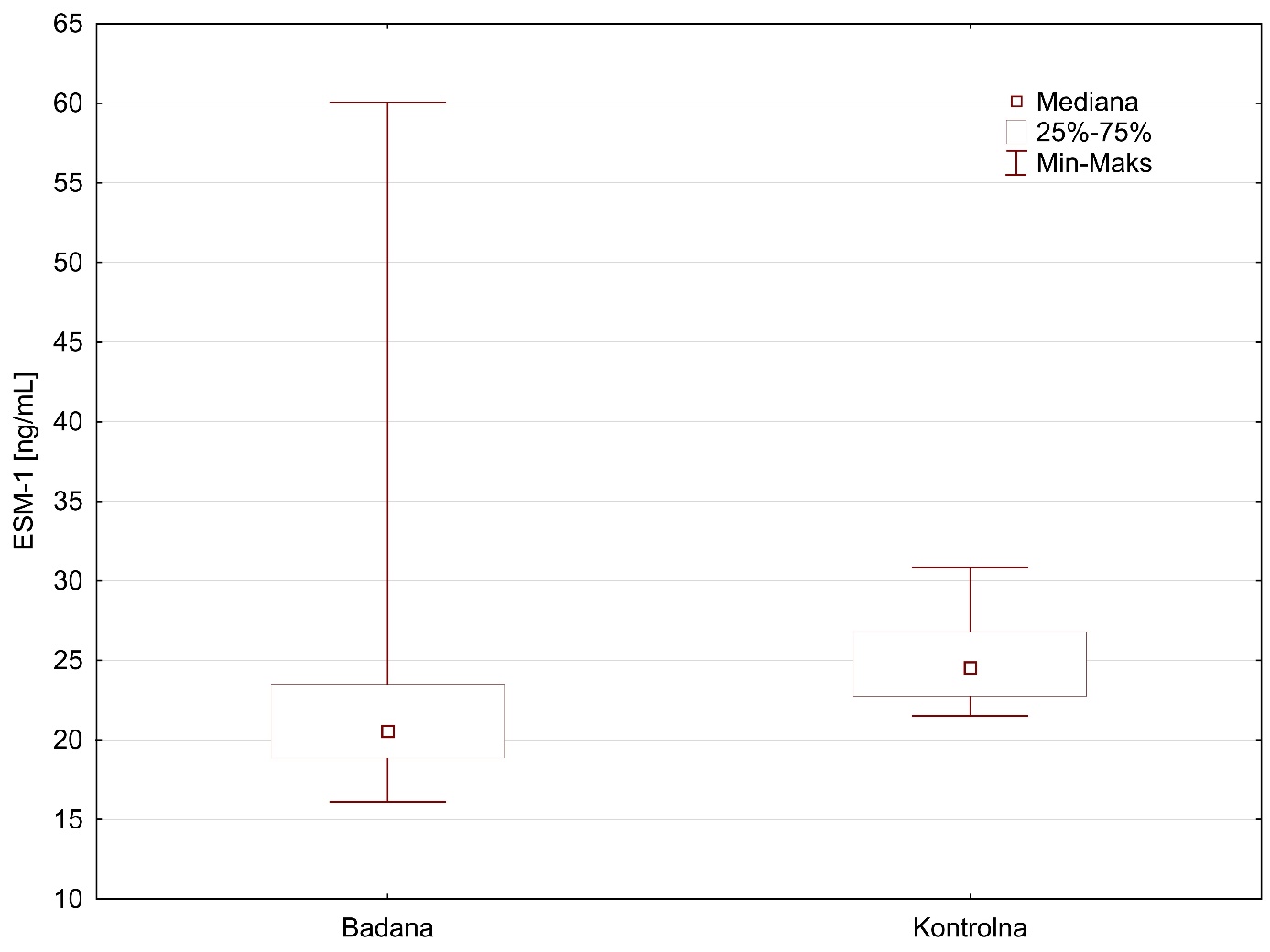

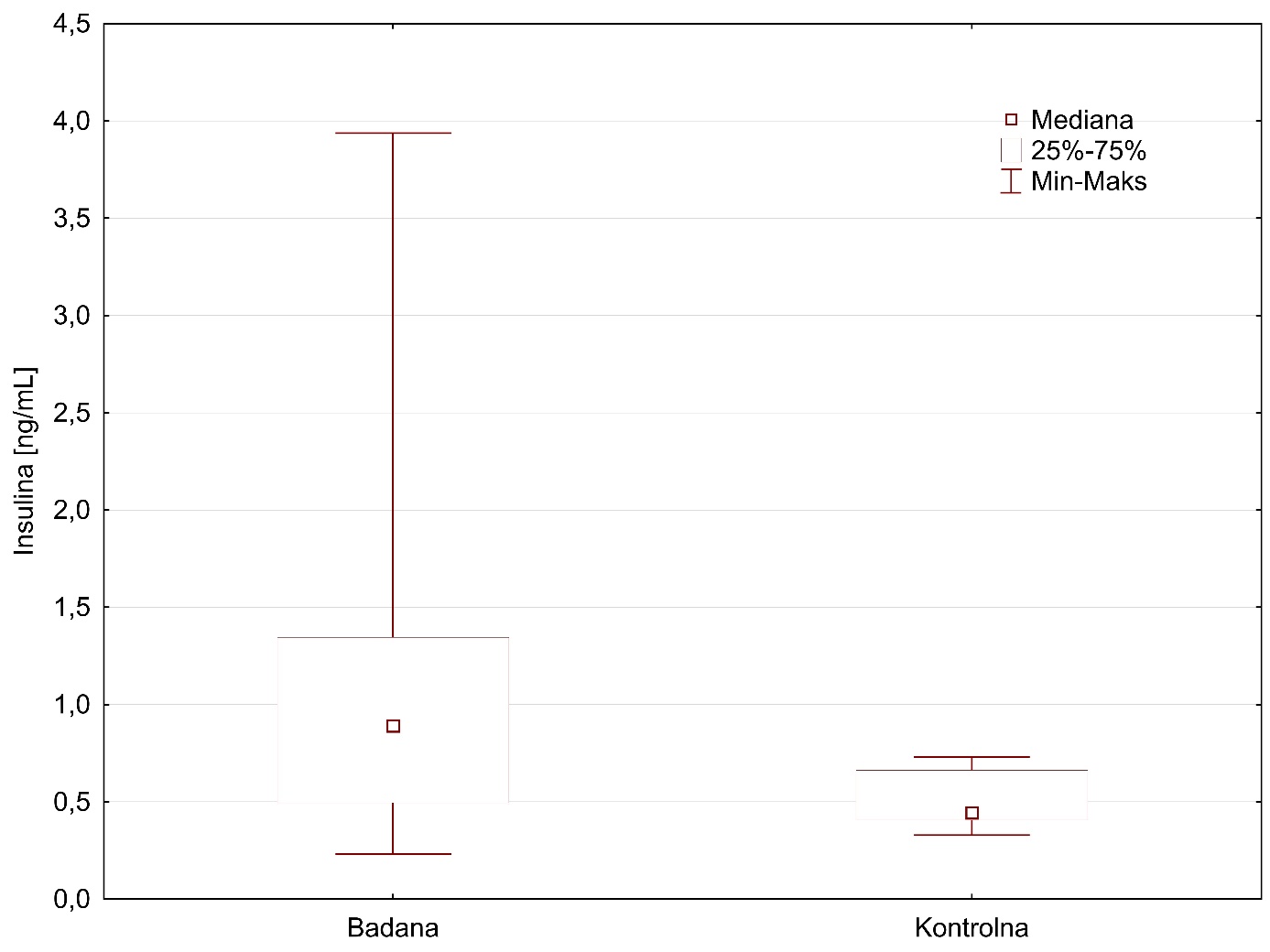

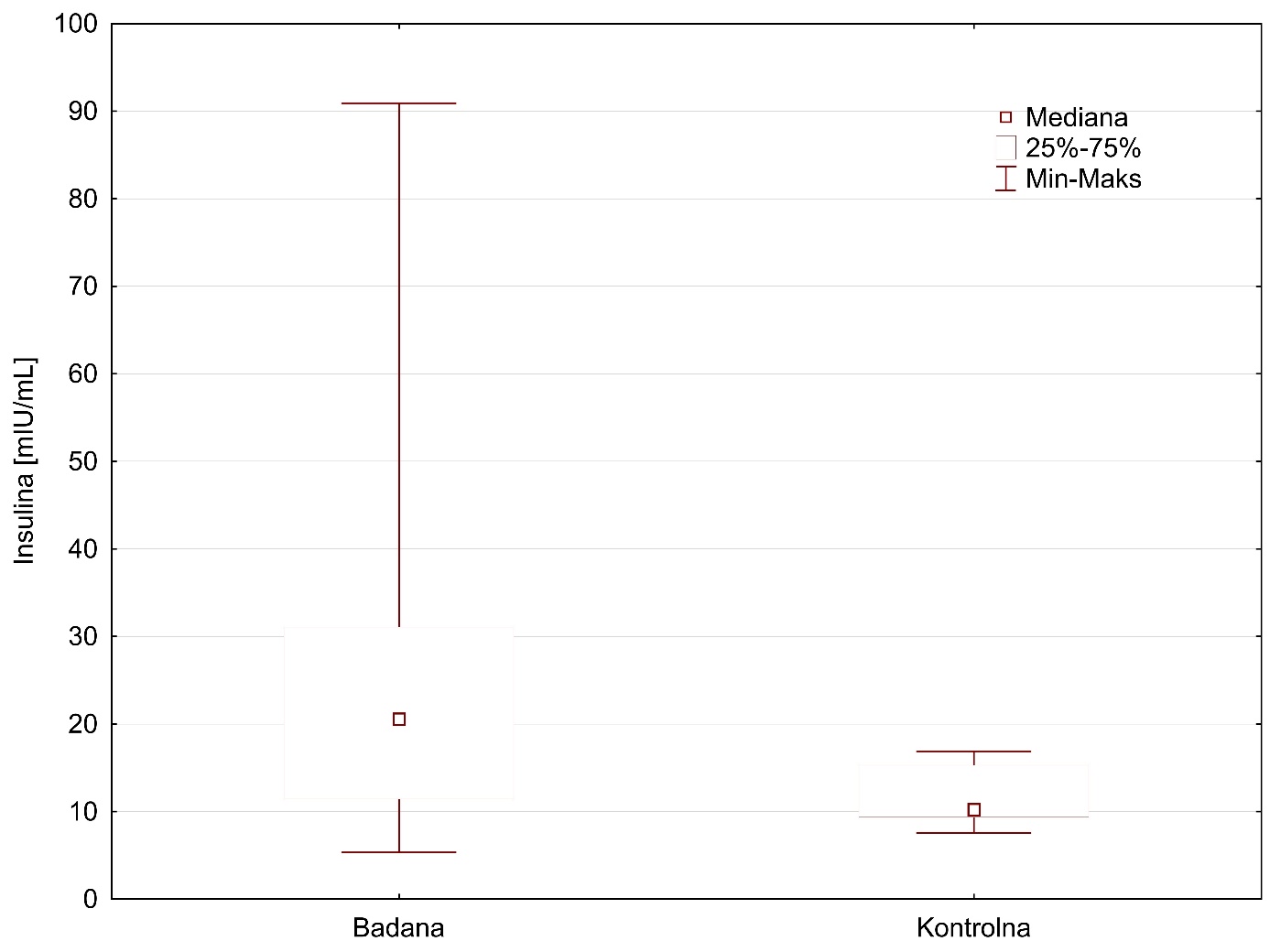

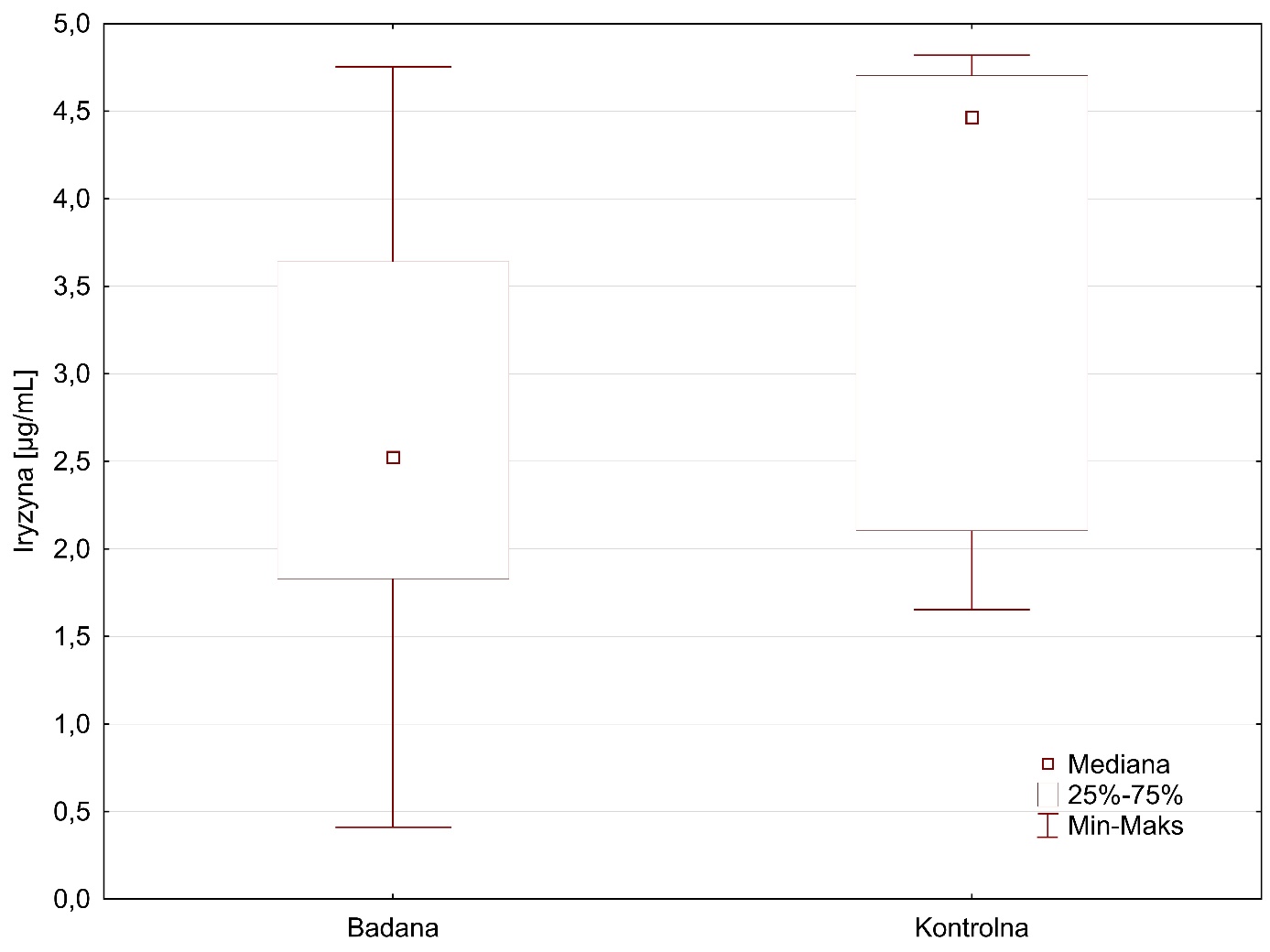

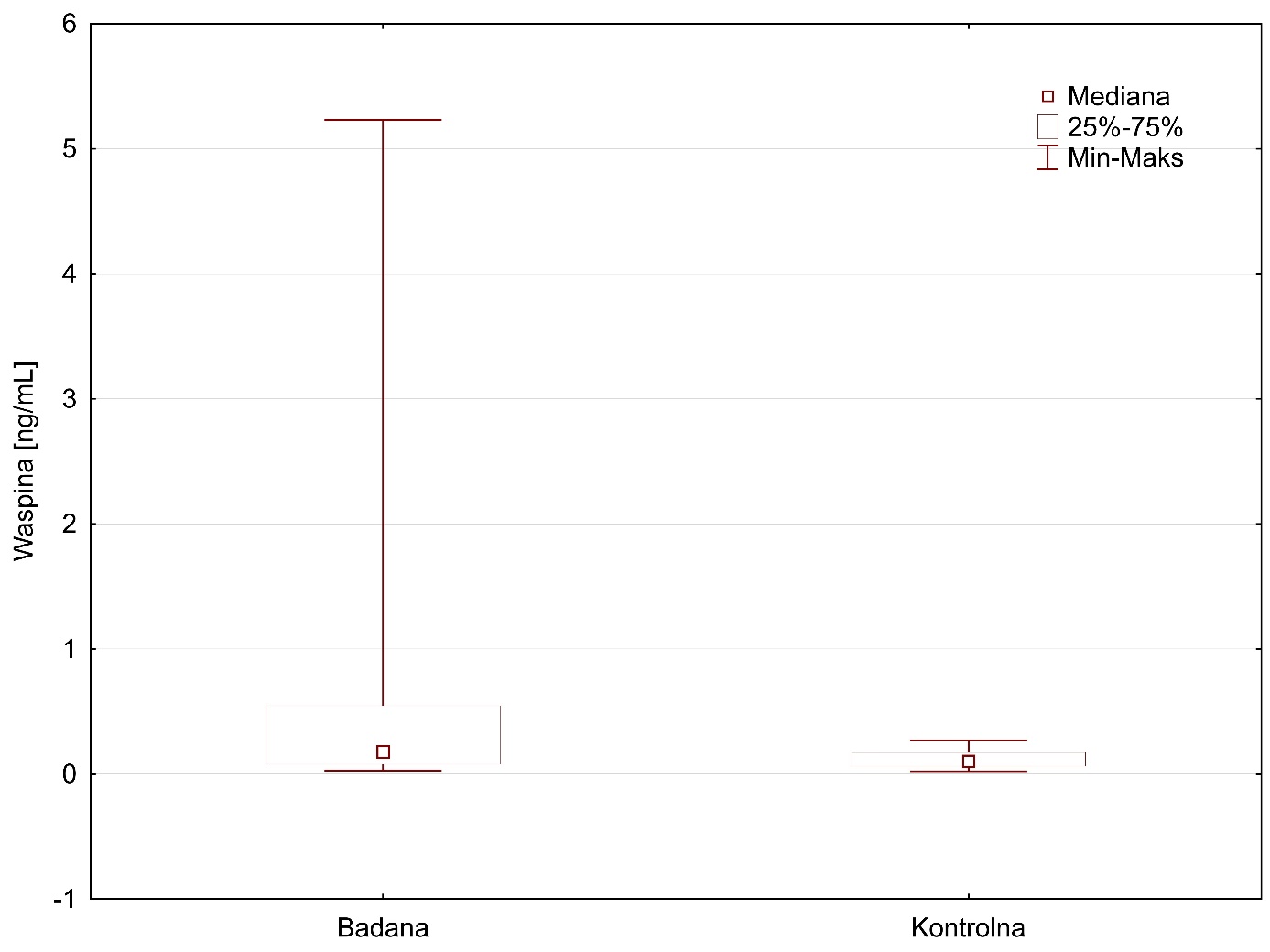

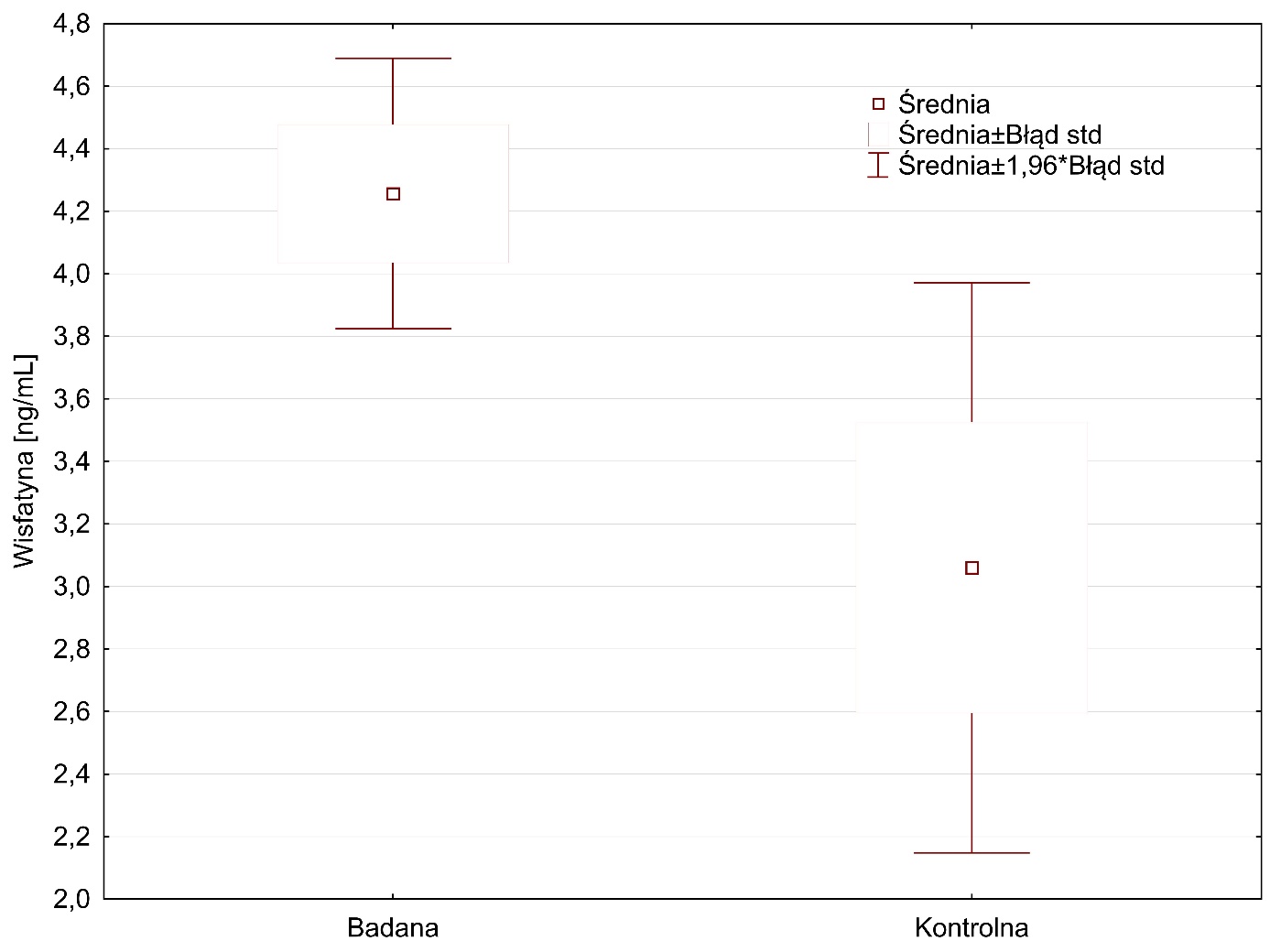


1-8 21-43

1. Kobiety i mężczyźni (porównanie kobiet i mężczyzn)

| KOBIETY | \| Nważnych \| \| --- \| | \| Średnia \| \| --- \| | \| Mediana \| \| --- \| | \| Minimum \| \| --- \| | \| Maksimum \| \| --- \| | \| Kwartyl. Rozstęp \| \| --- \| | \| Odch.std \| \| --- \| |
| --- | --- | --- | --- | --- | --- | --- | --- | --- | --- | --- | --- | --- | --- | --- |
| \| Waspina [ng/mL] \| \| --- \| | 14 | 0,5553 | 0,4169 | 0,12649 | 2,123 | 0,31622 | 0,532 |
| \| Wisfatyna [ng/mL] \| \| --- \| | 15 | 4,0376 | 4,2433 | 1,06407 | 7,484 | 3,57846 | 1,930 |
| \| Iryzyna [µg/mL] \| \| --- \| | 15 | 2,6889 | 2,4424 | 1,59332 | 4,753 | 1,85888 | 1,044 |
| \| ESM-1 [ng/mL] \| \| --- \| | 15 | 21,8362 | 20,7000 | 18,51667 | 29,683 | 3,40000 | 3,260 |
| \| Betatrofina [ng/mL] \| \| --- \| | 15 | 41,6223 | 46,1716 | 23,45596 | 49,904 | 15,06363 | 9,113 |
| \| Insulina [ng/mL] \| \| --- \| | 14 | 1,5564 | 1,3177 | 0,48066 | 3,642 | 0,60476 | 0,943 |
| \| Insulina [mIU/mL] \| \| --- \| | 15 | 33,8969 | 29,8538 | 5,22320 | 84,102 | 17,91232 | 22,444 |
| \| Wiek( lata) \| \| --- \| | 15 | 59,4000 | 59,0000 | 41,00000 | 77,000 | 9,00000 | 7,735 |
| \| WBC 106/ul \| \| --- \| | 15 | 3,8607 | 3,3800 | 1,78000 | 9,950 | 2,68000 | 2,133 |
| \| HGB mg/dl \| \| --- \| | 15 | 10,6800 | 10,1000 | 5,70000 | 14,700 | 2,60000 | 2,099 |
| \| PLT 106/ul \| \| --- \| | 15 | 81,3333 | 82,0000 | 18,00000 | 183,000 | 43,00000 | 38,505 |
| \| ALT IU/L \| \| --- \| | 15 | 73,4667 | 49,0000 | 22,00000 | 303,000 | 54,00000 | 72,800 |
| \| AST IU/L \| \| --- \| | 15 | 100,2000 | 68,0000 | 16,00000 | 409,000 | 71,00000 | 100,998 |
| \| FA IU/L \| \| --- \| | 15 | 125,7333 | 124,0000 | 38,00000 | 257,000 | 63,00000 | 56,907 |
| \| GGTP Iu/L \| \| --- \| | 15 | 70,1333 | 53,0000 | 21,00000 | 198,000 | 50,00000 | 53,243 |
| \| Glukoza mg/dl \| \| --- \| | 15 | 133,0400 | 119,4000 | 80,60000 | 267,900 | 63,60000 | 56,166 |
| \| Mocznik mg/dl \| \| --- \| | 15 | 33,8267 | 34,5000 | 16,60000 | 74,100 | 13,90000 | 14,220 |
| \| Kreatynina mg/dL \| \| --- \| | 15 | 0,6927 | 0,6400 | 0,54000 | 1,270 | 0,15000 | 0,176 |
| \| Bilirubina mg/dl \| \| --- \| | 15 | 2,8107 | 1,5800 | 0,58000 | 7,420 | 3,45000 | 2,123 |
| \| Cholesterol mg/dl \| \| --- \| | 15 | 137,3067 | 128,1000 | 98,30000 | 210,000 | 40,50000 | 31,020 |
| \| Triglicerydy mg/dl \| \| --- \| | 15 | 106,3800 | 101,0000 | 49,10000 | 180,000 | 54,70000 | 40,288 |
| \| HDL mg/dl \| \| --- \| | 15 | 33,8733 | 38,3000 | 7,80000 | 54,800 | 22,10000 | 13,707 |
| \| Pt % \| \| --- \| | 15 | 66,4467 | 67,0000 | 40,00000 | 102,800 | 30,00000 | 17,687 |
| \| Białko całkowite g/dl \| \| --- \| | 15 | 6,5313 | 6,8000 | 4,30000 | 7,800 | 0,87000 | 0,882 |
| \| Albumina g/dl \| \| --- \| | 15 | 2,9267 | 3,1000 | 1,90000 | 3,500 | 0,60000 | 0,486 |
| \| APF ng/ml \| \| --- \| | 15 | 610,2580 | 22,3800 | 2,09000 | 5845,000 | 43,08000 | 1619,242 |
| \| CEA ng/ml \| \| --- \| | 15 | 4,7040 | 4,5900 | 1,10000 | 12,230 | 2,91000 | 2,696 |
| \| CA 19,9 U/ml \| \| --- \| | 15 | 17,4167 | 12,0000 | 2,00000 | 51,310 | 16,38000 | 16,841 |
| \| BMI \| \| --- \| | 15 | 31,0667 | 30,0000 | 23,00000 | 45,000 | 6,00000 | 5,365 |
| \| Obwód pasa cm \| \| --- \| | 15 | 103,2000 | 99,0000 | 86,00000 | 139,000 | 15,00000 | 13,634 |
| \| HOMA-IR \| \| --- \| | 15 | 11,2485 | 8,6276 | 1,37738 | 25,714 | 11,73339 | 7,935 |

| MĘŻCZYŹNI | \| Nważnych \| \| --- \| | \| Średnia \| \| --- \| | \| Mediana \| \| --- \| | \| Minimum \| \| --- \| | \| Maksimum \| \| --- \| | \| Kwartyl. Rozstęp \| \| --- \| | \| Odch.std \| \| --- \|   T |
| --- | --- | --- | --- | --- | --- | --- | --- | --- | --- | --- | --- | --- | --- | --- |
| \| Waspina [ng/mL] \| \| --- \| | 52 | 0,5049 | 0,1290 | 0,02895 | 5,231 | 0,4862 | 1,0159 |
| \| Wisfatyna [ng/mL] \| \| --- \| | 51 | 4,3208 | 4,5149 | 0,51718 | 8,784 | 2,6226 | 1,7668 |
| \| Iryzyna [µg/mL] \| \| --- \| | 54 | 2,7376 | 2,5484 | 0,41129 | 4,729 | 1,7890 | 1,1693 |
| \| ESM-1 [ng/mL] \| \| --- \| | 52 | 23,4567 | 20,4083 | 16,11979 | 60,036 | 4,7750 | 9,3752 |
| \| Betatrofina [ng/mL] \| \| --- \| | 50 | 30,4489 | 26,2935 | 10,89534 | 49,963 | 23,1625 | 13,4407 |
| \| Insulina [ng/mL] \| \| --- \| | 54 | 1,0723 | 0,7880 | 0,23126 | 3,937 | 0,7701 | 0,8770 |
| \| Insulina [mIU/mL] \| \| --- \| | 54 | 24,7651 | 18,1987 | 5,34092 | 90,923 | 17,7846 | 20,2545 |
| \| Wiek( lata) \| \| --- \| | 54 | 60,4259 | 59,5000 | 20,00000 | 88,000 | 15,0000 | 13,1215 |
| \| WBC 106/ul \| \| --- \| | 53 | 5,3274 | 4,7500 | 1,86000 | 10,350 | 4,3000 | 2,4648 |
| \| HGB mg/dl \| \| --- \| | 54 | 12,1070 | 12,3000 | 3,16000 | 16,500 | 3,8000 | 2,4676 |
| \| PLT 106/ul \| \| --- \| | 53 | 124,1321 | 99,0000 | 31,00000 | 392,000 | 101,0000 | 92,2161 |
| \| ALT IU/L \| \| --- \| | 54 | 57,1296 | 44,5000 | 14,00000 | 222,000 | 47,0000 | 39,5174 |
| \| AST IU/L \| \| --- \| | 54 | 78,1481 | 62,0000 | 19,00000 | 258,000 | 68,0000 | 51,3057 |
| \| FA IU/L \| \| --- \| | 54 | 147,8519 | 109,0000 | 64,00000 | 476,000 | 79,0000 | 96,3980 |
| \| GGTP Iu/L \| \| --- \| | 54 | 172,0185 | 112,5000 | 23,00000 | 1243,000 | 101,0000 | 213,3931 |
| \| Glukoza mg/dl \| \| --- \| | 54 | 117,1685 | 103,8500 | 56,70000 | 281,000 | 44,0000 | 39,8172 |
| \| Mocznik mg/dl \| \| --- \| | 54 | 43,9185 | 36,2000 | 14,70000 | 229,300 | 18,2000 | 33,3843 |
| \| Kreatynina mg/dL \| \| --- \| | 54 | 1,5733 | 0,8050 | 0,49000 | 37,400 | 0,2600 | 4,9772 |
| \| Bilirubina mg/dl \| \| --- \| | 54 | 4,7943 | 1,5500 | 0,43000 | 152,100 | 1,3000 | 20,4870 |
| \| Cholesterol mg/dl \| \| --- \| | 54 | 157,5337 | 150,5000 | 2,02000 | 374,000 | 39,0000 | 53,6383 |
| \| Triglicerydy mg/dl \| \| --- \| | 54 | 119,5559 | 105,0000 | 40,00000 | 676,100 | 57,7000 | 86,4125 |
| \| HDL mg/dl \| \| --- \| | 54 | 44,4593 | 37,1500 | 12,20000 | 235,800 | 16,0000 | 34,0856 |
| \| Pt % \| \| --- \| | 54 | 76,5500 | 77,5000 | 48,00000 | 101,000 | 17,9000 | 12,2508 |
| \| Białko całkowite g/dl \| \| --- \| | 54 | 7,3085 | 7,3000 | 5,70000 | 9,800 | 0,9000 | 0,8847 |
| \| Albumina g/dl \| \| --- \| | 54 | 3,3030 | 3,1500 | 2,00000 | 4,600 | 0,8000 | 0,5895 |
| \| APF ng/ml \| \| --- \| | 54 | 293,3733 | 11,6400 | 0,74000 | 5845,000 | 39,2000 | 983,4851 |
| \| CEA ng/ml \| \| --- \| | 54 | 3,1910 | 2,4650 | 0,16500 | 18,920 | 2,2000 | 2,9030 |
| \| CA 19,9 U/ml \| \| --- \| | 54 | 38,1609 | 17,4300 | 2,49000 | 728,700 | 22,2700 | 100,2952 |
| \| BMI \| \| --- \| | 54 | 29,1833 | 29,0000 | 22,00000 | 38,000 | 7,0000 | 4,1786 |
| \| Obwód pasa cm \| \| --- \| | 54 | 102,6111 | 103,5000 | 70,00000 | 130,000 | 18,0000 | 12,3844 |
| \| HOMA-IR \| \| --- \| | 54 | 7,5696 | 4,8773 | 1,62206 | 48,205 | 5,0651 | 8,0375 |

| ZMIENNA | K | M | TEST | P |
| --- | --- | --- | --- | --- |
| \| Waspina [ng/mL] \| \| --- \| | N | N | U Manna Whitneya | 0,00901315756 |
| \| Wisfatyna [ng/mL] \| \| --- \| | T | T | T Studenta | 0,594766493 |
| \| Iryzyna [µg/mL] \| \| --- \| | N | T | U Manna Whitneya | 0,782233872 |
| \| ESM-1 [ng/mL] \| \| --- \| | N | N | U Manna Whitneya | 0,624950936 |
| \| Betatrofina [ng/mL] \| \| --- \| | N | N | U Manna Whitneya | 0,0075798303 |
| \| Insulina [ng/mL] \| \| --- \| | N | N | U Manna Whitneya | 0,0140074768 |
| \| Insulina [mIU/mL] \| \| --- \| | N | N | U Manna Whitneya | 0,0495354714 |
| \| Wiek( lata) \| \| --- \| | T | T | T Studenta | 0,77403953 |
| \| WBC 106/ul \| \| --- \| | N | N | U Manna Whitneya | 0,033801982 |
| \| HGB mg/dl \| \| --- \| | T | N | U Manna Whitneya | 0,0374947912 |
| \| PLT 106/ul \| \| --- \| | T | N | U Manna Whitneya | 0,193066761 |
| \| ALT IU/L \| \| --- \| | N | N | U Manna Whitneya | 0,555735378 |
| \| AST IU/L \| \| --- \| | N | N | U Manna Whitneya | 0,710659814 |
| \| FA IU/L \| \| --- \| | T | N | U Manna Whitneya | 0,861414245 |
| \| GGTP Iu/L \| \| --- \| | N | N | U Manna Whitneya | 0,00388034009 |
| \| Glukoza mg/dl \| \| --- \| | N | N | U Manna Whitneya | 0,436386577 |
| \| Mocznik mg/dl \| \| --- \| | N | N | U Manna Whitneya | 0,178407663 |
| \| Kreatynina mg/dL \| \| --- \| | N | N | U Manna Whitneya | 0,000275912851 |
| \| Bilirubina mg/dl \| \| --- \| | N | N | U Manna Whitneya | 0,235761216 |
| \| Cholesterol mg/dl \| \| --- \| | T | N | U Manna Whitneya | 0,0585956429 |
| \| Triglicerydy mg/dl \| \| --- \| | T | N | U Manna Whitneya | 0,67310711 |
| \| HDL mg/dl \| \| --- \| | T | N | U Manna Whitneya | 0,250441035 |
| \| Pt % \| \| --- \| | T | T | T Studenta | 0,0130192602 |
| \| Białko całkowite g/dl \| \| --- \| | T | T | T Studenta | 0,00366447516 |
| \| Albumina g/dl \| \| --- \| | T | T | T Studenta | 0,0268114886 |
| \| APF ng/ml \| \| --- \| | N | N | U Manna Whitneya | 0,394742342 |
| \| CEA ng/ml \| \| --- \| | N | N | U Manna Whitneya | 0,00810428791 |
| \| CA 19,9 U/ml \| \| --- \| | N | N | U Manna Whitneya | 0,141743258 |
| \| BMI \| \| --- \| | T | T | T Studenta | 0,15194491 |
| \| Obwód pasa cm \| \| --- \| | T | T | T Studenta | 0,873811514 |
| \| HOMA-IR \| \| --- \| | T | N | U Manna Whitneya | 0,0368331082 |

1. Wirusy i nie wirusy HCV i HBSag (porównanie pacjentów z HCC w przebiegu wzw B lub C a grupą pacjentów z HCC w przebiegu innych chorób)

| Zmienna | Wirusy=Tak | | | | | | |
| --- | --- | --- | --- | --- | --- | --- | --- |
|  | \| Nważnych \| \| --- \| | \| Średnia \| \| --- \| | \| Mediana \| \| --- \| | \| Minimum \| \| --- \| | \| Maksimum \| \| --- \| | \| Kwartyl. Rozstęp \| \| --- \| | \| Odch.std \| \| --- \| |
| \| Waspina [ng/mL] \| \| --- \| | 41,00 | 0,5542 | 0,2320 | 0,02895 | 5,231 | 0,4611 | 0,881 |
| \| Wisfatyna [ng/mL] \| \| --- \| | 43,00 | 4,3437 | 4,6235 | 0,51718 | 8,784 | 2,9708 | 1,839 |
| \| Iryzyna [µg/mL] \| \| --- \| | 43,00 | 2,5303 | 2,1193 | 0,41129 | 4,753 | 1,6735 | 1,127 |
| \| ESM-1 [ng/mL] \| \| --- \| | 43,00 | 22,6877 | 20,7000 | 16,11979 | 60,036 | 4,8167 | 7,468 |
| \| Betatrofina [ng/mL] \| \| --- \| | 40,00 | 34,8948 | 34,8488 | 10,96007 | 49,963 | 25,2633 | 12,932 |
| \| Insulina [ng/mL] \| \| --- \| | 42,00 | 1,1776 | 0,8810 | 0,30818 | 3,937 | 0,7776 | 0,952 |
| \| Insulina [mIU/mL] \| \| --- \| | 43,00 | 26,6853 | 19,7334 | 5,22320 | 90,923 | 18,4221 | 21,988 |
| \| Wiek( lata) \| \| --- \| | 43,00 | 60,4186 | 59,0000 | 20,00000 | 88,000 | 11,0000 | 12,807 |
| \| WBC 106/ul \| \| --- \| | 42,00 | 4,8357 | 4,1400 | 1,78000 | 10,350 | 3,0700 | 2,557 |
| \| HGB mg/dl \| \| --- \| | 43,00 | 11,4037 | 11,9000 | 3,16000 | 15,100 | 3,6000 | 2,434 |
| \| PLT 106/ul \| \| --- \| | 42,00 | 106,2857 | 82,0000 | 18,00000 | 392,000 | 76,0000 | 77,973 |
| \| ALT IU/L \| \| --- \| | 43,00 | 73,5581 | 60,0000 | 14,00000 | 303,000 | 56,0000 | 55,520 |
| \| AST IU/L \| \| --- \| | 43,00 | 101,9302 | 92,0000 | 16,00000 | 409,000 | 88,0000 | 73,096 |
| \| FA IU/L \| \| --- \| | 43,00 | 141,5349 | 110,0000 | 38,00000 | 427,000 | 75,0000 | 82,918 |
| \| GGTP Iu/L \| \| --- \| | 43,00 | 128,3721 | 86,0000 | 26,00000 | 482,000 | 108,0000 | 106,612 |
| \| Glukoza mg/dl \| \| --- \| | 43,00 | 116,3186 | 106,8000 | 56,70000 | 267,900 | 45,7000 | 40,877 |
| \| Mocznik mg/dl \| \| --- \| | 43,00 | 42,5047 | 35,3000 | 16,60000 | 229,300 | 17,8000 | 33,695 |
| \| Kreatynina mg/dL \| \| --- \| | 43,00 | 1,6651 | 0,7500 | 0,49000 | 37,400 | 0,2400 | 5,586 |
| \| Bilirubina mg/dl \| \| --- \| | 43,00 | 5,8170 | 1,5200 | 0,43000 | 152,100 | 2,2400 | 22,928 |
| \| Cholesterol mg/dl \| \| --- \| | 43,00 | 154,5888 | 154,0000 | 2,02000 | 374,000 | 50,0000 | 59,614 |
| \| Triglicerydy mg/dl \| \| --- \| | 43,00 | 104,9186 | 107,0000 | 40,00000 | 180,000 | 64,9000 | 39,568 |
| \| HDL mg/dl \| \| --- \| | 43,00 | 40,9814 | 38,3000 | 12,20000 | 177,600 | 14,0000 | 23,593 |
| \| Pt % \| \| --- \| | 43,00 | 73,9674 | 71,0000 | 40,00000 | 102,800 | 18,9000 | 13,981 |
| \| Białko całkowite g/dl \| \| --- \| | 43,00 | 7,0984 | 7,1000 | 4,30000 | 9,800 | 1,3000 | 1,038 |
| \| Albumina g/dl \| \| --- \| | 43,00 | 3,0419 | 3,0000 | 1,90000 | 4,200 | 0,6000 | 0,501 |
| \| APF ng/ml \| \| --- \| | 43,00 | 574,7258 | 37,6900 | 2,09000 | 5845,000 | 168,4000 | 1414,166 |
| \| CEA ng/ml \| \| --- \| | 43,00 | 4,0971 | 3,1100 | 0,16500 | 18,920 | 2,7000 | 3,355 |
| \| CA 19,9 U/ml \| \| --- \| | 43,00 | 24,1330 | 14,2700 | 2,00000 | 200,000 | 21,2100 | 32,389 |
| \| BMI \| \| --- \| | 43,00 | 29,8814 | 29,0000 | 22,00000 | 45,000 | 6,0000 | 4,735 |
| \| Obwód pasa cm \| \| --- \| | 43,00 | 103,2093 | 102,0000 | 70,00000 | 139,000 | 18,0000 | 13,328 |
| \| HOMA-IR \| \| --- \| | 43,00 | 7,8314 | 5,3036 | 1,37738 | 25,714 | 7,5174 | 6,720 |

| Zmienna | Wirusy=Nie | | | | | | |
| --- | --- | --- | --- | --- | --- | --- | --- |
|  | \| Nważnych \| \| --- \| | \| Średnia \| \| --- \| | \| Mediana \| \| --- \| | \| Minimum \| \| --- \| | \| Maksimum \| \| --- \| | \| Kwartyl. Rozstęp \| \| --- \| | \| Odch.std \| \| --- \| |
| \| Waspina [ng/mL] \| \| --- \| | 25 | 0,45 | 0,10 | 0,03 | 4,9 | 0,17 | 1,02 |
| \| Wisfatyna [ng/mL] \| \| --- \| | 23 | 4,09 | 4,48 | 1,06 | 7,2 | 2,78 | 1,73 |
| \| Iryzyna [µg/mL] \| \| --- \| | 26 | 3,05 | 3,02 | 0,89 | 4,7 | 1,92 | 1,10 |
| \| ESM-1 [ng/mL] \| \| --- \| | 24 | 23,82 | 20,52 | 17,78 | 55,8 | 4,04 | 10,00 |
| \| Betatrofina [ng/mL] \| \| --- \| | 25 | 30,04 | 26,09 | 10,90 | 49,6 | 23,40 | 13,80 |
| \| Insulina [ng/mL] \| \| --- \| | 26 | 1,16 | 0,92 | 0,23 | 3,0 | 0,88 | 0,84 |
| \| Insulina [mIU/mL] \| \| --- \| | 26 | 26,86 | 21,30 | 5,34 | 69,5 | 20,32 | 19,46 |
| \| Wiek( lata) \| \| --- \| | 26 | 59,85 | 59,50 | 24,00 | 84,0 | 15,00 | 11,10 |
| \| WBC 106/ul \| \| --- \| | 26 | 5,28 | 4,82 | 1,97 | 9,5 | 4,09 | 2,31 |
| \| HGB mg/dl \| \| --- \| | 26 | 12,45 | 12,45 | 8,30 | 16,5 | 4,10 | 2,38 |
| \| PLT 106/ul \| \| --- \| | 26 | 128,27 | 101,00 | 31,00 | 389,0 | 98,00 | 95,36 |
| \| ALT IU/L \| \| --- \| | 26 | 39,38 | 31,50 | 15,00 | 100,0 | 23,00 | 21,64 |
| \| AST IU/L \| \| --- \| | 26 | 51,54 | 40,50 | 20,00 | 146,0 | 36,00 | 29,98 |
| \| FA IU/L \| \| --- \| | 26 | 145,54 | 108,00 | 64,00 | 476,0 | 70,00 | 100,91 |
| \| GGTP Iu/L \| \| --- \| | 26 | 185,42 | 97,50 | 21,00 | 1243,0 | 112,00 | 286,00 |
| \| Glukoza mg/dl \| \| --- \| | 26 | 127,73 | 111,90 | 80,60 | 281,0 | 46,80 | 48,52 |
| \| Mocznik mg/dl \| \| --- \| | 26 | 40,43 | 35,75 | 14,70 | 139,1 | 23,90 | 24,77 |
| \| Kreatynina mg/dL \| \| --- \| | 26 | 0,91 | 0,83 | 0,59 | 2,2 | 0,28 | 0,33 |
| \| Bilirubina mg/dl \| \| --- \| | 26 | 1,96 | 1,64 | 0,48 | 4,8 | 1,23 | 1,16 |
| \| Cholesterol mg/dl \| \| --- \| | 26 | 150,73 | 143,25 | 100,70 | 220,0 | 34,90 | 29,16 |
| \| Triglicerydy mg/dl \| \| --- \| | 26 | 136,16 | 104,75 | 70,60 | 676,1 | 56,50 | 116,44 |
| \| HDL mg/dl \| \| --- \| | 26 | 44,10 | 35,15 | 7,80 | 235,8 | 20,30 | 40,98 |
| \| Pt % \| \| --- \| | 26 | 74,99 | 78,00 | 40,00 | 101,0 | 19,00 | 14,57 |
| \| Białko całkowite g/dl \| \| --- \| | 26 | 7,21 | 7,20 | 5,40 | 8,5 | 0,90 | 0,75 |
| \| Albumina g/dl \| \| --- \| | 26 | 3,52 | 3,55 | 2,30 | 4,6 | 0,90 | 0,61 |
| \| APF ng/ml \| \| --- \| | 26 | 10,88 | 4,82 | 0,74 | 41,0 | 9,07 | 13,06 |
| \| CEA ng/ml \| \| --- \| | 26 | 2,57 | 2,30 | 0,50 | 7,0 | 2,53 | 1,61 |
| \| CA 19,9 U/ml \| \| --- \| | 26 | 49,39 | 15,93 | 3,31 | 728,7 | 21,72 | 139,68 |
| \| BMI \| \| --- \| | 26 | 29,12 | 28,50 | 22,00 | 38,0 | 7,00 | 4,09 |
| \| Obwód pasa cm \| \| --- \| | 26 | 101,96 | 99,50 | 80,00 | 130,0 | 15,00 | 11,40 |
| \| HOMA-IR \| \| --- \| | 26 | 9,26 | 5,49 | 1,62 | 48,2 | 9,02 | 10,07 |

| ZMIENNA | Wirus=TAK | Wirus=Nie | TEST | P |
| --- | --- | --- | --- | --- |
| \| Waspina [ng/mL] \| \| --- \| | N | N | U Manna Whitneya | 0,0229889171 |
| \| Wisfatyna [ng/mL] \| \| --- \| | T | T | T Studenta | 0,593133204 |
| \| Iryzyna [µg/mL] \| \| --- \| | N | T | U Manna Whitneya | 0,318780094 |
| \| ESM-1 [ng/mL] \| \| --- \| | N | N | U Manna Whitneya | 0,0015907348 |
| \| Betatrofina [ng/mL] \| \| --- \| | N | N | U Manna Whitneya | 0,000426877113 |
| \| Insulina [ng/mL] \| \| --- \| | N | N | U Manna Whitneya | 0,896550723 |
| \| Insulina [mIU/mL] \| \| --- \| | N | N | U Manna Whitneya | 0,81399607 |
| \| Wiek( lata) \| \| --- \| | T | N | U Manna Whitneya | 0,301151817 |
| \| WBC 106/ul \| \| --- \| | N | T | U Manna Whitneya | 0,955562642 |
| \| HGB mg/dl \| \| --- \| | N | T | U Manna Whitneya | 0,129293064 |
| \| PLT 106/ul \| \| --- \| | N | N | U Manna Whitneya | 0,85264659 |
| \| ALT IU/L \| \| --- \| | N | N | U Manna Whitneya | 0,867237567 |
| \| AST IU/L \| \| --- \| | N | N | U Manna Whitneya | 0,309918863 |
| \| FA IU/L \| \| --- \| | N | N | U Manna Whitneya | 0,77579326 |
| \| GGTP Iu/L \| \| --- \| | N | N | U Manna Whitneya | 0,318780094 |
| \| Glukoza mg/dl \| \| --- \| | N | N | U Manna Whitneya | 0,0015907348 |
| \| Mocznik mg/dl \| \| --- \| | N | N | U Manna Whitneya | 0,000426877113 |
| \| Kreatynina mg/dL \| \| --- \| | N | N | U Manna Whitneya | 0,896550723 |
| \| Bilirubina mg/dl \| \| --- \| | N | N | U Manna Whitneya | 0,81399607 |
| \| Cholesterol mg/dl \| \| --- \| | N | T | U Manna Whitneya | 0,301151817 |
| \| Triglicerydy mg/dl \| \| --- \| | T | N | U Manna Whitneya | 0,955562642 |
| \| HDL mg/dl \| \| --- \| | N | N | U Manna Whitneya | 0,129293064 |
| \| Pt % \| \| --- \| | T | T | T Studenta | 0,772358834 |
| \| Białko całkowite g/dl \| \| --- \| | T | T | T Studenta | 0,641432844 |
| \| Albumina g/dl \| \| --- \| | T | T | T Studenta | 0,000755544707 |
| \| APF ng/ml \| \| --- \| | N | N | U Manna Whitneya | 0,0000193710301 |
| \| CEA ng/ml \| \| --- \| | N | T | U Manna Whitneya | 0,0266558816 |
| \| CA 19,9 U/ml \| \| --- \| | N | N | U Manna Whitneya | 0,346655969 |
| \| BMI \| \| --- \| | T | T | T Studenta | 0,496166388 |
| \| Obwód pasa cm \| \| --- \| | T | T | T Studenta | 0,692441831 |
| \| HOMA-IR \| \| --- \| | N | N | U Manna Whitneya | 0,678269885 |

1. HCV i I niewirusy – wykluczyć HBSag (porównanie pomiędzy pacjentami z HCC w przebiegu wzw C, wykluczone wzw B)

| Zmienna | HCV=Tak Wykluczenie: HBSAg='Tak' | | | | | | |
| --- | --- | --- | --- | --- | --- | --- | --- |
|  | \| Nważnych \| \| --- \| | \| Średnia \| \| --- \| | \| Mediana \| \| --- \| | \| Minimum \| \| --- \| | \| Maksimum \| \| --- \| | \| Kwartyl. Rozstęp \| \| --- \| | \| Odch.std \| \| --- \| |
| \| Waspina [ng/mL] \| \| --- \| | 33 | 0,621 | 0,408 | 0,0381 | 5,23 | 0,457 | 0,94 |
| \| Wisfatyna [ng/mL] \| \| --- \| | 35 | 4,312 | 4,430 | 0,5172 | 8,78 | 2,971 | 1,93 |
| \| Iryzyna [µg/mL] \| \| --- \| | 35 | 2,589 | 2,119 | 0,4113 | 4,75 | 1,670 | 1,10 |
| \| ESM-1 [ng/mL] \| \| --- \| | 35 | 23,191 | 20,883 | 16,1198 | 60,04 | 4,997 | 8,16 |
| \| Betatrofina [ng/mL] \| \| --- \| | 32 | 37,508 | 39,220 | 10,9601 | 49,96 | 23,634 | 11,95 |
| \| Insulina [ng/mL] \| \| --- \| | 34 | 1,137 | 0,972 | 0,3437 | 3,77 | 0,778 | 0,84 |
| \| Insulina [mIU/mL] \| \| --- \| | 35 | 25,663 | 20,962 | 5,2232 | 87,03 | 18,422 | 19,55 |
| \| Wiek( lata) \| \| --- \| | 35 | 60,000 | 59,000 | 20,0000 | 88,00 | 9,000 | 12,88 |
| \| WBC 106/ul \| \| --- \| | 34 | 4,282 | 3,610 | 1,7800 | 10,35 | 2,530 | 2,30 |
| \| HGB mg/dl \| \| --- \| | 35 | 11,459 | 12,000 | 3,1600 | 15,10 | 3,400 | 2,56 |
| \| PLT 106/ul \| \| --- \| | 34 | 86,941 | 75,000 | 18,0000 | 198,00 | 62,000 | 47,63 |
| \| ALT IU/L \| \| --- \| | 35 | 79,114 | 63,000 | 21,0000 | 303,00 | 50,000 | 57,56 |
| \| AST IU/L \| \| --- \| | 35 | 104,514 | 101,000 | 16,0000 | 409,00 | 84,000 | 70,35 |
| \| FA IU/L \| \| --- \| | 35 | 129,600 | 106,000 | 38,0000 | 427,00 | 55,000 | 78,15 |
| \| GGTP Iu/L \| \| --- \| | 35 | 110,229 | 80,000 | 26,0000 | 354,00 | 76,000 | 83,99 |
| \| Glukoza mg/dl \| \| --- \| | 35 | 122,234 | 112,300 | 76,4000 | 267,90 | 40,100 | 42,09 |
| \| Mocznik mg/dl \| \| --- \| | 35 | 45,089 | 35,500 | 16,6000 | 229,30 | 16,400 | 36,84 |
| \| Kreatynina mg/dL \| \| --- \| | 35 | 1,864 | 0,750 | 0,4900 | 37,40 | 0,270 | 6,19 |
| \| Bilirubina mg/dl \| \| --- \| | 35 | 6,440 | 1,520 | 0,5800 | 152,10 | 1,350 | 25,40 |
| \| Cholesterol mg/dl \| \| --- \| | 35 | 146,815 | 146,000 | 2,0200 | 374,00 | 51,300 | 59,78 |
| \| Triglicerydy mg/dl \| \| --- \| | 35 | 104,286 | 107,000 | 46,0000 | 180,00 | 64,900 | 37,62 |
| \| HDL mg/dl \| \| --- \| | 35 | 42,823 | 39,700 | 12,2000 | 177,60 | 15,200 | 25,44 |
| \| Pt % \| \| --- \| | 35 | 72,963 | 71,000 | 44,0000 | 102,80 | 15,000 | 12,67 |
| \| Białko całkowite g/dl \| \| --- \| | 35 | 7,014 | 7,000 | 4,3000 | 9,80 | 1,400 | 1,04 |
| \| Albumina g/dl \| \| --- \| | 35 | 2,989 | 3,000 | 1,9000 | 4,20 | 0,700 | 0,49 |
| \| APF ng/ml \| \| --- \| | 35 | 528,134 | 37,870 | 2,0900 | 5845,00 | 168,640 | 1262,91 |
| \| CEA ng/ml \| \| --- \| | 35 | 4,415 | 3,320 | 0,1650 | 18,92 | 3,220 | 3,60 |
| \| CA 19,9 U/ml \| \| --- \| | 35 | 18,012 | 13,870 | 2,0000 | 65,60 | 20,760 | 17,06 |
| \| BMI \| \| --- \| | 35 | 29,254 | 29,000 | 22,0000 | 38,00 | 5,000 | 3,90 |
| \| Obwód pasa cm \| \| --- \| | 35 | 100,686 | 99,000 | 70,0000 | 120,00 | 20,000 | 12,08 |
| \| HOMA-IR \| \| --- \| | 35 | 8,055 | 6,113 | 1,3774 | 25,71 | 7,022 | 6,68 |

| Zmienna | HCV=Nie Wykluczenie : HBSAg='Tak' | | | | | | |
| --- | --- | --- | --- | --- | --- | --- | --- |
|  | \| Nważnych \| \| --- \| | \| Średnia \| \| --- \| | \| Mediana \| \| --- \| | \| Minimum \| \| --- \| | \| Maksimum \| \| --- \| | \| Kwartyl. Rozstęp \| \| --- \| | \| Odch.std \| \| --- \| |
| \| Waspina [ng/mL] \| \| --- \| | 25 | 0,452 | 0,099 | 0,030 | 4,88 | 0,171 | 1,023 |
| \| Wisfatyna [ng/mL] \| \| --- \| | 23 | 4,093 | 4,480 | 1,064 | 7,17 | 2,781 | 1,734 |
| \| Iryzyna [µg/mL] \| \| --- \| | 26 | 3,052 | 3,020 | 0,889 | 4,70 | 1,917 | 1,095 |
| \| ESM-1 [ng/mL] \| \| --- \| | 24 | 23,822 | 20,518 | 17,783 | 55,80 | 4,042 | 10,000 |
| \| Betatrofina [ng/mL] \| \| --- \| | 25 | 30,039 | 26,093 | 10,895 | 49,62 | 23,402 | 13,800 |
| \| Insulina [ng/mL] \| \| --- \| | 26 | 1,163 | 0,922 | 0,231 | 3,01 | 0,880 | 0,843 |
| \| Insulina [mIU/mL] \| \| --- \| | 26 | 26,858 | 21,299 | 5,341 | 69,48 | 20,321 | 19,460 |
| \| Wiek( lata) \| \| --- \| | 26 | 59,846 | 59,500 | 24,000 | 84,00 | 15,000 | 11,102 |
| \| WBC 106/ul \| \| --- \| | 26 | 5,275 | 4,820 | 1,970 | 9,47 | 4,090 | 2,312 |
| \| HGB mg/dl \| \| --- \| | 26 | 12,447 | 12,450 | 8,300 | 16,50 | 4,100 | 2,382 |
| \| PLT 106/ul \| \| --- \| | 26 | 128,269 | 101,000 | 31,000 | 389,00 | 98,000 | 95,361 |
| \| ALT IU/L \| \| --- \| | 26 | 39,385 | 31,500 | 15,000 | 100,00 | 23,000 | 21,643 |
| \| AST IU/L \| \| --- \| | 26 | 51,538 | 40,500 | 20,000 | 146,00 | 36,000 | 29,983 |
| \| FA IU/L \| \| --- \| | 26 | 145,538 | 108,000 | 64,000 | 476,00 | 70,000 | 100,908 |
| \| GGTP Iu/L \| \| --- \| | 26 | 185,423 | 97,500 | 21,000 | 1243,00 | 112,000 | 285,998 |
| \| Glukoza mg/dl \| \| --- \| | 26 | 127,731 | 111,900 | 80,600 | 281,00 | 46,800 | 48,522 |
| \| Mocznik mg/dl \| \| --- \| | 26 | 40,435 | 35,750 | 14,700 | 139,10 | 23,900 | 24,772 |
| \| Kreatynina mg/dL \| \| --- \| | 26 | 0,913 | 0,830 | 0,590 | 2,24 | 0,280 | 0,333 |
| \| Bilirubina mg/dl \| \| --- \| | 26 | 1,958 | 1,640 | 0,480 | 4,77 | 1,230 | 1,160 |
| \| Cholesterol mg/dl \| \| --- \| | 26 | 150,735 | 143,250 | 100,700 | 220,00 | 34,900 | 29,160 |
| \| Triglicerydy mg/dl \| \| --- \| | 26 | 136,162 | 104,750 | 70,600 | 676,10 | 56,500 | 116,438 |
| \| HDL mg/dl \| \| --- \| | 26 | 44,104 | 35,150 | 7,800 | 235,80 | 20,300 | 40,981 |
| \| Pt % \| \| --- \| | 26 | 74,992 | 78,000 | 40,000 | 101,00 | 19,000 | 14,567 |
| \| Białko całkowite g/dl \| \| --- \| | 26 | 7,208 | 7,200 | 5,400 | 8,50 | 0,900 | 0,749 |
| \| Albumina g/dl \| \| --- \| | 26 | 3,518 | 3,550 | 2,300 | 4,60 | 0,900 | 0,606 |
| \| APF ng/ml \| \| --- \| | 26 | 10,878 | 4,820 | 0,740 | 41,03 | 9,070 | 13,061 |
| \| CEA ng/ml \| \| --- \| | 26 | 2,565 | 2,295 | 0,500 | 6,97 | 2,530 | 1,606 |
| \| CA 19,9 U/ml \| \| --- \| | 26 | 49,393 | 15,930 | 3,310 | 728,70 | 21,720 | 139,679 |
| \| BMI \| \| --- \| | 26 | 29,115 | 28,500 | 22,000 | 38,00 | 7,000 | 4,092 |
| \| Obwód pasa cm \| \| --- \| | 26 | 101,962 | 99,500 | 80,000 | 130,00 | 15,000 | 11,400 |
| \| HOMA-IR \| \| --- \| | 26 | 9,259 | 5,490 | 1,622 | 48,21 | 9,017 | 10,069 |

| ZMIENNA | HCV – TAK  HBS AG Nie | Brak wirusa | TEST | P |
| --- | --- | --- | --- | --- |
| \| Waspina [ng/mL] \| \| --- \| | N | N | U Manna Whitneya | 0,00300196283 |
| \| Wisfatyna [ng/mL] \| \| --- \| | T | T | T Studenta | 0,662033704 |
| \| Iryzyna [µg/mL] \| \| --- \| | N | T | U Manna Whitneya | 0,127504961 |
| \| ESM-1 [ng/mL] \| \| --- \| | N | N | U Manna Whitneya | 0,810973768 |
| \| Betatrofina [ng/mL] \| \| --- \| | N | N | U Manna Whitneya | 0,0228730825 |
| \| Insulina [ng/mL] \| \| --- \| | N | N | U Manna Whitneya | 0,89909895 |
| \| Insulina [mIU/mL] \| \| --- \| | N | N | U Manna Whitneya | 0,959290732 |
| \| Wiek( lata) \| \| --- \| | T | N | U Manna Whitneya | 0,861074273 |
| \| WBC 106/ul \| \| --- \| | N | T | U Manna Whitneya | 0,0676392776 |
| \| HGB mg/dl \| \| --- \| | N | T | U Manna Whitneya | 0,217822749 |
| \| PLT 106/ul \| \| --- \| | N | N | U Manna Whitneya | 0,0992721852 |
| \| ALT IU/L \| \| --- \| | N | N | U Manna Whitneya | 0,000183486536 |
| \| AST IU/L \| \| --- \| | N | N | U Manna Whitneya | 0,00010476939 |
| \| FA IU/L \| \| --- \| | N | N | U Manna Whitneya | 0,645952087 |
| \| GGTP Iu/L \| \| --- \| | N | N | U Manna Whitneya | 0,781707833 |
| \| Glukoza mg/dl \| \| --- \| | N | N | U Manna Whitneya | 0,798554517 |
| \| Mocznik mg/dl \| \| --- \| | N | N | U Manna Whitneya | 0,688377967 |
| \| Kreatynina mg/dL \| \| --- \| | N | N | U Manna Whitneya | 0,152940972 |
| \| Bilirubina mg/dl \| \| --- \| | N | N | U Manna Whitneya | 0,832523212 |
| \| Cholesterol mg/dl \| \| --- \| | N | T | U Manna Whitneya | 0,569510851 |
| \| Triglicerydy mg/dl \| \| --- \| | T | N | U Manna Whitneya | 0,307314934 |
| \| HDL mg/dl \| \| --- \| | N | N | U Manna Whitneya | 0,521073516 |
| \| Pt % \| \| --- \| | T | T | T Studenta | 0,563843192 |
| \| Białko całkowite g/dl \| \| --- \| | T | T | T Studenta | 0,423394942 |
| \| Albumina g/dl \| \| --- \| | T | T | T Studenta | 0,000395158962 |
| \| APF ng/ml \| \| --- \| | N | N | U Manna Whitneya | 0,0000728524314 |
| \| CEA ng/ml \| \| --- \| | N | T | U Manna Whitneya | 0,0137140334 |
| \| CA 19,9 U/ml \| \| --- \| | N | N | U Manna Whitneya | 0,105488187 |
| \| BMI \| \| --- \| | T | T | T Studenta | 0,893345962 |
| \| Obwód pasa cm \| \| --- \| | T | T | T Studenta | 0,677703464 |
| \| HOMA-IR \| \| --- \| | N | N | U Manna Whitneya | 0,912901679 |

1. Cukzyca i bez

| Zmienna | Cukrzyca t,II=Tak | | | | | | |
| --- | --- | --- | --- | --- | --- | --- | --- |
|  | \| Nważnych \| \| --- \| | \| Średnia \| \| --- \| | \| Mediana \| \| --- \| | \| Minimum \| \| --- \| | \| Maksimum \| \| --- \| | \| Kwartyl. Rozstęp \| \| --- \| | \| Odch.std \| \| --- \| |
| \| Waspina [ng/mL] \| \| --- \| | 26 | 0,56 | 0,15 | 0,029 | 4,9 | 0,45 | 1,0 |
| \| Wisfatyna [ng/mL] \| \| --- \| | 26 | 4,59 | 4,81 | 1,142 | 7,5 | 2,17 | 1,5 |
| \| Iryzyna [µg/mL] \| \| --- \| | 27 | 2,73 | 2,44 | 0,893 | 4,7 | 2,32 | 1,2 |
| \| ESM-1 [ng/mL] \| \| --- \| | 26 | 22,30 | 20,41 | 17,350 | 55,2 | 4,12 | 7,3 |
| \| Betatrofina [ng/mL] \| \| --- \| | 27 | 31,94 | 30,23 | 10,895 | 49,9 | 24,59 | 13,9 |
| \| Insulina [ng/mL] \| \| --- \| | 27 | 1,35 | 1,10 | 0,231 | 3,6 | 1,31 | 1,0 |
| \| Insulina [mIU/mL] \| \| --- \| | 27 | 31,09 | 25,49 | 5,341 | 84,1 | 30,27 | 22,2 |
| \| Wiek( lata) \| \| --- \| | 27 | 59,41 | 59,00 | 41,000 | 73,0 | 14,00 | 9,2 |
| \| WBC 106/ul \| \| --- \| | 26 | 5,04 | 4,42 | 1,950 | 10,4 | 4,29 | 2,5 |
| \| HGB mg/dl \| \| --- \| | 27 | 11,37 | 12,10 | 3,160 | 16,1 | 4,00 | 2,9 |
| \| PLT 106/ul \| \| --- \| | 26 | 112,65 | 93,50 | 18,000 | 306,0 | 67,00 | 76,3 |
| \| ALT IU/L \| \| --- \| | 27 | 58,26 | 42,00 | 20,000 | 222,0 | 55,00 | 46,3 |
| \| AST IU/L \| \| --- \| | 27 | 77,70 | 58,00 | 19,000 | 258,0 | 80,00 | 60,5 |
| \| FA IU/L \| \| --- \| | 27 | 146,37 | 122,00 | 55,000 | 476,0 | 73,00 | 90,0 |
| \| GGTP Iu/L \| \| --- \| | 27 | 213,70 | 124,00 | 21,000 | 1243,0 | 136,00 | 284,2 |
| \| Glukoza mg/dl \| \| --- \| | 27 | 148,26 | 136,10 | 56,700 | 281,0 | 53,60 | 56,7 |
| \| Mocznik mg/dl \| \| --- \| | 27 | 42,47 | 32,50 | 16,600 | 229,3 | 21,10 | 39,6 |
| \| Kreatynina mg/dL \| \| --- \| | 27 | 2,17 | 0,76 | 0,570 | 37,4 | 0,21 | 7,0 |
| \| Bilirubina mg/dl \| \| --- \| | 27 | 2,15 | 1,50 | 0,600 | 7,4 | 1,23 | 1,8 |
| \| Cholesterol mg/dl \| \| --- \| | 27 | 147,87 | 142,30 | 2,020 | 230,0 | 48,40 | 45,6 |
| \| Triglicerydy mg/dl \| \| --- \| | 27 | 135,34 | 123,20 | 40,000 | 676,1 | 45,00 | 114,6 |
| \| HDL mg/dl \| \| --- \| | 27 | 39,25 | 34,00 | 7,800 | 177,6 | 16,40 | 29,6 |
| \| Pt % \| \| --- \| | 27 | 75,96 | 78,00 | 40,000 | 96,0 | 19,00 | 12,4 |
| \| Białko całkowite g/dl \| \| --- \| | 27 | 6,84 | 7,00 | 4,300 | 8,1 | 1,20 | 0,9 |
| \| Albumina g/dl \| \| --- \| | 27 | 3,24 | 3,20 | 1,900 | 4,6 | 0,90 | 0,7 |
| \| APF ng/ml \| \| --- \| | 27 | 554,89 | 8,66 | 0,740 | 5845,0 | 33,47 | 1613,8 |
| \| CEA ng/ml \| \| --- \| | 27 | 2,95 | 2,83 | 0,165 | 7,0 | 2,51 | 1,7 |
| \| CA 19,9 U/ml \| \| --- \| | 27 | 55,17 | 15,00 | 2,000 | 728,7 | 36,25 | 140,2 |
| \| BMI \| \| --- \| | 27 | 30,18 | 30,00 | 22,900 | 38,0 | 6,00 | 4,2 |
| \| Obwód pasa cm \| \| --- \| | 27 | 105,44 | 106,00 | 86,000 | 130,0 | 19,00 | 10,8 |
| \| HOMA-IR \| \| --- \| | 27 | 11,97 | 8,43 | 1,622 | 48,2 | 13,88 | 10,6 |

| Zmienna | Cukrzyca t,II=Nie | | | | | | |
| --- | --- | --- | --- | --- | --- | --- | --- |
|  | \| Nważnych \| \| --- \| | \| Średnia \| \| --- \| | \| Mediana \| \| --- \| | \| Minimum \| \| --- \| | \| Maksimum \| \| --- \| | \| Kwartyl. Rozstęp \| \| --- \| | \| Odch.std \| \| --- \| |
| \| Waspina [ng/mL] \| \| --- \| | 40 | 0,49 | 0,19 | 0,032 | 5,2 | 0,531 | 0,87 |
| \| Wisfatyna [ng/mL] \| \| --- \| | 40 | 4,04 | 4,13 | 0,517 | 8,8 | 2,792 | 1,93 |
| \| Iryzyna [µg/mL] \| \| --- \| | 42 | 2,73 | 2,68 | 0,411 | 4,8 | 1,676 | 1,12 |
| \| ESM-1 [ng/mL] \| \| --- \| | 41 | 23,60 | 20,88 | 16,120 | 60,0 | 4,783 | 9,11 |
| \| Betatrofina [ng/mL] \| \| --- \| | 38 | 33,80 | 34,09 | 11,265 | 50,0 | 25,257 | 13,12 |
| \| Insulina [ng/mL] \| \| --- \| | 41 | 1,06 | 0,85 | 0,323 | 3,9 | 0,800 | 0,86 |
| \| Insulina [mIU/mL] \| \| --- \| | 42 | 23,96 | 18,91 | 5,223 | 90,9 | 18,507 | 19,85 |
| \| Wiek( lata) \| \| --- \| | 42 | 60,71 | 59,00 | 20,000 | 88,0 | 10,000 | 13,74 |
| \| WBC 106/ul \| \| --- \| | 42 | 4,98 | 4,20 | 1,780 | 10,0 | 3,400 | 2,48 |
| \| HGB mg/dl \| \| --- \| | 42 | 12,07 | 12,25 | 8,300 | 16,5 | 3,700 | 2,09 |
| \| PLT 106/ul \| \| --- \| | 42 | 115,95 | 84,50 | 34,000 | 392,0 | 83,000 | 90,89 |
| \| ALT IU/L \| \| --- \| | 42 | 62,24 | 53,00 | 14,000 | 303,0 | 52,000 | 50,36 |
| \| AST IU/L \| \| --- \| | 42 | 86,31 | 67,50 | 16,000 | 409,0 | 64,000 | 68,38 |
| \| FA IU/L \| \| --- \| | 42 | 140,90 | 106,50 | 38,000 | 427,0 | 74,000 | 90,06 |
| \| GGTP Iu/L \| \| --- \| | 42 | 108,83 | 82,50 | 23,000 | 354,0 | 86,000 | 84,75 |
| \| Glukoza mg/dl \| \| --- \| | 42 | 102,85 | 98,30 | 76,400 | 148,1 | 22,700 | 18,27 |
| \| Mocznik mg/dl \| \| --- \| | 42 | 41,24 | 36,45 | 14,700 | 139,1 | 16,200 | 23,31 |
| \| Kreatynina mg/dL \| \| --- \| | 42 | 0,88 | 0,80 | 0,490 | 2,2 | 0,290 | 0,35 |
| \| Bilirubina mg/dl \| \| --- \| | 42 | 5,79 | 1,82 | 0,430 | 152,1 | 1,770 | 23,19 |
| \| Cholesterol mg/dl \| \| --- \| | 42 | 156,52 | 152,50 | 69,500 | 374,0 | 45,100 | 53,08 |
| \| Triglicerydy mg/dl \| \| --- \| | 42 | 104,70 | 101,00 | 46,000 | 228,0 | 59,700 | 39,35 |
| \| HDL mg/dl \| \| --- \| | 42 | 44,03 | 38,90 | 12,200 | 235,8 | 15,700 | 32,14 |
| \| Pt % \| \| --- \| | 42 | 73,32 | 70,50 | 40,000 | 102,8 | 19,000 | 15,17 |
| \| Białko całkowite g/dl \| \| --- \| | 42 | 7,33 | 7,20 | 5,800 | 9,8 | 0,900 | 0,91 |
| \| Albumina g/dl \| \| --- \| | 42 | 3,21 | 3,10 | 2,000 | 4,5 | 0,600 | 0,53 |
| \| APF ng/ml \| \| --- \| | 42 | 238,43 | 28,93 | 1,010 | 3340,0 | 68,110 | 694,81 |
| \| CEA ng/ml \| \| --- \| | 42 | 3,89 | 2,98 | 0,500 | 18,9 | 2,690 | 3,43 |
| \| CA 19,9 U/ml \| \| --- \| | 42 | 19,82 | 15,48 | 2,000 | 65,6 | 16,590 | 16,24 |
| \| BMI \| \| --- \| | 42 | 29,21 | 28,00 | 22,000 | 45,0 | 6,000 | 4,68 |
| \| Obwód pasa cm \| \| --- \| | 42 | 101,00 | 98,50 | 70,000 | 139,0 | 18,000 | 13,40 |
| \| HOMA-IR \| \| --- \| | 42 | 6,05 | 4,52 | 1,377 | 20,1 | 4,947 | 4,79 |

| ZMIENNA | Cukrzyca TAK | Cukrzyca NIE | TEST | P |
| --- | --- | --- | --- | --- |
| \| Waspina [ng/mL] \| \| --- \| | N | N | U Manna Whitneya | 0,650731189 |
| \| Wisfatyna [ng/mL] \| \| --- \| | T | T | T Studenta | 0,228774842 |
| \| Iryzyna [µg/mL] \| \| --- \| | N | T | U Manna Whitneya | 0,810519486 |
| \| ESM-1 [ng/mL] \| \| --- \| | N | N | U Manna Whitneya | 0,647845493 |
| \| Betatrofina [ng/mL] \| \| --- \| | N | N | U Manna Whitneya | 0,744314675 |
| \| Insulina [ng/mL] \| \| --- \| | N | N | U Manna Whitneya | 0,177848211 |
| \| Insulina [mIU/mL] \| \| --- \| | N | N | U Manna Whitneya | 0,136826419 |
| \| Wiek( lata) \| \| --- \| | T | N | U Manna Whitneya | 0,680421276 |
| \| WBC 106/ul \| \| --- \| | N | N | U Manna Whitneya | 0,904571366 |
| \| HGB mg/dl \| \| --- \| | T | T | T Studenta | 0,248533624 |
| \| PLT 106/ul \| \| --- \| | N | N | U Manna Whitneya | 0,728556743 |
| \| ALT IU/L \| \| --- \| | N | N | U Manna Whitneya | 0,622855836 |
| \| AST IU/L \| \| --- \| | N | N | U Manna Whitneya | 0,36615724 |
| \| FA IU/L \| \| --- \| | N | N | U Manna Whitneya | 0,550964243 |
| \| GGTP Iu/L \| \| --- \| | N | N | U Manna Whitneya | 0,0944966729 |
| \| Glukoza mg/dl \| \| --- \| | T | N | U Manna Whitneya | 0,00013814775 |
| \| Mocznik mg/dl \| \| --- \| | N | N | U Manna Whitneya | 0,410066659 |
| \| Kreatynina mg/dL \| \| --- \| | N | N | U Manna Whitneya | 0,931413769 |
| \| Bilirubina mg/dl \| \| --- \| | N | N | U Manna Whitneya | 0,689456197 |
| \| Cholesterol mg/dl \| \| --- \| | N | N | U Manna Whitneya | 0,622855836 |
| \| Triglicerydy mg/dl \| \| --- \| | N | T | U Manna Whitneya | 0,172327706 |
| \| HDL mg/dl \| \| --- \| | N | N | U Manna Whitneya | 0,087446224 |
| \| Pt % \| \| --- \| | T | T | T Studenta | 0,452501346 |
| \| Białko całkowite g/dl \| \| --- \| | T | N | U Manna Whitneya | 0,125832041 |
| \| Albumina g/dl \| \| --- \| | T | T | T Studenta | 0,806519906 |
| \| APF ng/ml \| \| --- \| | N | N | U Manna Whitneya | 0,16472551 |
| \| CEA ng/ml \| \| --- \| | T | N | U Manna Whitneya | 0,4349535 |
| \| CA 19,9 U/ml \| \| --- \| | N | N | U Manna Whitneya | 0,58428586 |
| \| BMI \| \| --- \| | T | N | U Manna Whitneya | 0,176225845 |
| \| Obwód pasa cm \| \| --- \| | T | T | T Studenta | 0,152997977 |
| \| HOMA-IR \| \| --- \| | N | N | U Manna Whitneya | 0,0119251677 |

1. HomaIR do >4 i pow <=4 (policzyć Homa IR = Insulina z kolumny J x glukoza/405)
   1. Jeżeli za mało będzie z 3 to przesunąć na 4

| Zmienna | HOMA-IR <4 | | | | | | |
| --- | --- | --- | --- | --- | --- | --- | --- |
|  | \| Nważnych \| \| --- \| | \| Średnia \| \| --- \| | \| Mediana \| \| --- \| | \| Minimum \| \| --- \| | \| Maksimum \| \| --- \| | \| Kwartyl. Rozstęp \| \| --- \| | \| Odch.std \| \| --- \| |
| \| Waspina [ng/mL] \| \| --- \| | 23 | 0,52 | 0,14 | 0,029 | 4,9 | 0,60 | 1,0 |
| \| Wisfatyna [ng/mL] \| \| --- \| | 23 | 4,88 | 4,81 | 1,975 | 8,8 | 2,57 | 1,6 |
| \| Iryzyna [µg/mL] \| \| --- \| | 25 | 2,61 | 2,42 | 0,411 | 4,8 | 1,61 | 1,2 |
| \| ESM-1 [ng/mL] \| \| --- \| | 25 | 24,75 | 23,47 | 17,783 | 55,8 | 5,38 | 8,3 |
| \| Betatrofina [ng/mL] \| \| --- \| | 22 | 28,37 | 24,14 | 10,960 | 50,0 | 20,93 | 13,3 |
| \| Insulina [ng/mL] \| \| --- \| | 24 | 0,49 | 0,48 | 0,231 | 0,9 | 0,18 | 0,1 |
| \| Insulina [mIU/mL] \| \| --- \| | 25 | 11,03 | 11,10 | 5,223 | 19,7 | 4,22 | 3,6 |
| \| Wiek( lata) \| \| --- \| | 25 | 60,72 | 59,00 | 24,000 | 85,0 | 12,00 | 13,2 |
| \| WBC 106/ul \| \| --- \| | 25 | 5,84 | 5,35 | 2,250 | 10,4 | 4,45 | 2,5 |
| \| HGB mg/dl \| \| --- \| | 25 | 11,78 | 12,20 | 8,300 | 16,5 | 3,40 | 2,4 |
| \| PLT 106/ul \| \| --- \| | 25 | 151,80 | 130,00 | 35,000 | 392,0 | 100,00 | 97,9 |
| \| ALT IU/L \| \| --- \| | 25 | 73,20 | 63,00 | 14,000 | 303,0 | 64,00 | 59,9 |
| \| AST IU/L \| \| --- \| | 25 | 107,44 | 94,00 | 19,000 | 409,0 | 96,00 | 85,5 |
| \| FA IU/L \| \| --- \| | 25 | 146,80 | 95,00 | 38,000 | 427,0 | 130,00 | 105,1 |
| \| GGTP Iu/L \| \| --- \| | 25 | 147,80 | 122,00 | 23,000 | 482,0 | 137,00 | 130,5 |
| \| Glukoza mg/dl \| \| --- \| | 25 | 96,62 | 97,60 | 56,700 | 136,1 | 18,30 | 17,6 |
| \| Mocznik mg/dl \| \| --- \| | 25 | 39,07 | 35,30 | 14,700 | 109,0 | 12,00 | 19,3 |
| \| Kreatynina mg/dL \| \| --- \| | 25 | 0,88 | 0,80 | 0,540 | 2,0 | 0,15 | 0,3 |
| \| Bilirubina mg/dl \| \| --- \| | 25 | 2,51 | 1,90 | 0,430 | 9,7 | 2,70 | 2,2 |
| \| Cholesterol mg/dl \| \| --- \| | 25 | 178,34 | 170,90 | 85,000 | 374,0 | 69,00 | 59,3 |
| \| Triglicerydy mg/dl \| \| --- \| | 25 | 114,59 | 115,00 | 40,000 | 228,0 | 63,00 | 47,0 |
| \| HDL mg/dl \| \| --- \| | 25 | 38,46 | 35,70 | 12,200 | 55,0 | 18,10 | 12,4 |
| \| Pt % \| \| --- \| | 25 | 78,98 | 80,00 | 44,000 | 102,8 | 19,00 | 16,0 |
| \| Białko całkowite g/dl \| \| --- \| | 25 | 7,19 | 7,06 | 5,800 | 9,8 | 0,50 | 0,8 |
| \| Albumina g/dl \| \| --- \| | 25 | 3,24 | 3,10 | 2,000 | 4,6 | 0,70 | 0,6 |
| \| APF ng/ml \| \| --- \| | 25 | 583,98 | 37,08 | 1,010 | 5845,0 | 170,33 | 1357,6 |
| \| CEA ng/ml \| \| --- \| | 25 | 3,40 | 2,34 | 0,165 | 12,2 | 2,56 | 3,0 |
| \| CA 19,9 U/ml \| \| --- \| | 25 | 32,31 | 20,20 | 2,830 | 200,0 | 28,84 | 40,2 |
| \| BMI \| \| --- \| | 25 | 30,40 | 29,00 | 23,000 | 38,0 | 5,00 | 4,0 |
| \| Obwód pasa cm \| \| --- \| | 25 | 104,64 | 107,00 | 82,000 | 127,0 | 15,00 | 10,4 |
| \| HOMA-IR \| \| --- \| | 25 | 2,54 | 2,54 | 1,377 | 3,8 | 0,76 | 0,7 |

| Zmienna | HOMA-IR >4 | | | | | | |
| --- | --- | --- | --- | --- | --- | --- | --- |
|  | \| Nważnych \| \| --- \| | \| Średnia \| \| --- \| | \| Mediana \| \| --- \| | \| Minimum \| \| --- \| | \| Maksimum \| \| --- \| | \| Kwartyl. Rozstęp \| \| --- \| | \| Odch.std \| \| --- \| |
| \| Waspina [ng/mL] \| \| --- \| | 43,0 | 0,51 | 0,18 | 0,030 | 5,2 | 0,471 | 0,88 |
| \| Wisfatyna [ng/mL] \| \| --- \| | 43,0 | 3,92 | 4,01 | 0,517 | 7,5 | 2,926 | 1,83 |
| \| Iryzyna [µg/mL] \| \| --- \| | 44,0 | 2,79 | 2,60 | 0,889 | 4,7 | 1,844 | 1,11 |
| \| ESM-1 [ng/mL] \| \| --- \| | 42,0 | 22,11 | 20,16 | 16,120 | 60,0 | 2,867 | 8,43 |
| \| Betatrofina [ng/mL] \| \| --- \| | 43,0 | 35,41 | 37,69 | 10,895 | 50,0 | 24,807 | 12,94 |
| \| Insulina [ng/mL] \| \| --- \| | 44,0 | 1,55 | 1,25 | 0,472 | 3,9 | 0,971 | 0,93 |
| \| Insulina [mIU/mL] \| \| --- \| | 44,0 | 35,68 | 28,87 | 10,911 | 90,9 | 22,416 | 21,44 |
| \| Wiek( lata) \| \| --- \| | 44,0 | 59,91 | 59,50 | 20,000 | 88,0 | 9,500 | 11,60 |
| \| WBC 106/ul \| \| --- \| | 43,0 | 4,52 | 3,91 | 1,780 | 10,0 | 2,570 | 2,32 |
| \| HGB mg/dl \| \| --- \| | 44,0 | 11,81 | 12,05 | 3,160 | 16,1 | 3,950 | 2,52 |
| \| PLT 106/ul \| \| --- \| | 43,0 | 93,12 | 74,00 | 18,000 | 319,0 | 53,000 | 68,98 |
| \| ALT IU/L \| \| --- \| | 44,0 | 53,57 | 42,00 | 15,000 | 222,0 | 42,000 | 39,74 |
| \| AST IU/L \| \| --- \| | 44,0 | 69,02 | 55,50 | 16,000 | 202,0 | 52,000 | 45,47 |
| \| FA IU/L \| \| --- \| | 44,0 | 140,91 | 111,00 | 55,000 | 476,0 | 58,500 | 80,39 |
| \| GGTP Iu/L \| \| --- \| | 44,0 | 151,05 | 88,50 | 21,000 | 1243,0 | 77,500 | 224,43 |
| \| Glukoza mg/dl \| \| --- \| | 44,0 | 134,25 | 123,00 | 79,100 | 281,0 | 52,200 | 48,50 |
| \| Mocznik mg/dl \| \| --- \| | 44,0 | 43,23 | 35,40 | 16,600 | 229,3 | 20,950 | 35,39 |
| \| Kreatynina mg/dL \| \| --- \| | 44,0 | 1,67 | 0,74 | 0,490 | 37,4 | 0,260 | 5,52 |
| \| Bilirubina mg/dl \| \| --- \| | 44,0 | 5,42 | 1,55 | 0,480 | 152,1 | 1,160 | 22,67 |
| \| Cholesterol mg/dl \| \| --- \| | 44,0 | 138,81 | 141,15 | 2,020 | 220,0 | 41,800 | 37,84 |
| \| Triglicerydy mg/dl \| \| --- \| | 44,0 | 117,88 | 101,00 | 46,000 | 676,1 | 52,250 | 92,42 |
| \| HDL mg/dl \| \| --- \| | 44,0 | 44,26 | 38,20 | 7,800 | 235,8 | 15,300 | 37,76 |
| \| Pt % \| \| --- \| | 44,0 | 71,73 | 70,00 | 40,000 | 96,0 | 14,000 | 12,35 |
| \| Białko całkowite g/dl \| \| --- \| | 44,0 | 7,11 | 7,20 | 4,300 | 9,8 | 1,300 | 1,00 |
| \| Albumina g/dl \| \| --- \| | 44,0 | 3,21 | 3,15 | 1,900 | 4,5 | 0,700 | 0,57 |
| \| APF ng/ml \| \| --- \| | 44,0 | 236,29 | 8,29 | 0,740 | 5845,0 | 34,885 | 999,84 |
| \| CEA ng/ml \| \| --- \| | 44,0 | 3,59 | 3,16 | 0,700 | 18,9 | 2,255 | 2,88 |
| \| CA 19,9 U/ml \| \| --- \| | 44,0 | 34,42 | 14,07 | 2,000 | 728,7 | 16,885 | 108,19 |
| \| BMI \| \| --- \| | 44,0 | 29,13 | 28,00 | 22,000 | 45,0 | 5,500 | 4,74 |
| \| Obwód pasa cm \| \| --- \| | 44,0 | 101,66 | 99,00 | 70,000 | 139,0 | 18,000 | 13,63 |
| \| HOMA-IR \| \| --- \| | 44,0 | 11,68 | 7,90 | 4,426 | 48,2 | 10,960 | 8,52 |

| ZMIENNA | Homa<4 | Homa>4 | TEST | P |
| --- | --- | --- | --- | --- |
| \| Waspina [ng/mL] \| \| --- \| | N | N | U Manna Whitneya | 0,756929349 |
| \| Wisfatyna [ng/mL] \| \| --- \| | T | T | T Studenta | 0,0372144981 |
| \| Iryzyna [µg/mL] \| \| --- \| | T | N | U Manna Whitneya | 0,675796925 |
| \| ESM-1 [ng/mL] \| \| --- \| | N | N | U Manna Whitneya | 0,0112620181 |
| \| Betatrofina [ng/mL] \| \| --- \| | N | N | U Manna Whitneya | 0,0843472004 |
| \| Insulina [ng/mL] \| \| --- \| | T | N | U Manna Whitneya | 0,00000000104754314 |
| \| Insulina [mIU/mL] \| \| --- \| | T | N | U Manna Whitneya | 0,000000000528241916 |
| \| Wiek( lata) \| \| --- \| | T | N | U Manna Whitneya | 0,925404592 |
| \| WBC 106/ul \| \| --- \| | T | N | U Manna Whitneya | 0,0247720593 |
| \| HGB mg/dl \| \| --- \| | T | N | U Manna Whitneya | 0,565797174 |
| \| PLT 106/ul \| \| --- \| | N | N | U Manna Whitneya | 0,00160856317 |
| \| ALT IU/L \| \| --- \| | N | N | U Manna Whitneya | 0,127755142 |
| \| AST IU/L \| \| --- \| | N | N | U Manna Whitneya | 0,0394165831 |
| \| FA IU/L \| \| --- \| | N | N | U Manna Whitneya | 0,23318424 |
| \| GGTP Iu/L \| \| --- \| | N | N | U Manna Whitneya | 0,694143743 |
| \| Glukoza mg/dl \| \| --- \| | T | N | U Manna Whitneya | 0,000260756101 |
| \| Mocznik mg/dl \| \| --- \| | N | N | U Manna Whitneya | 0,940292307 |
| \| Kreatynina mg/dL \| \| --- \| | N | N | U Manna Whitneya | 0,413537117 |
| \| Bilirubina mg/dl \| \| --- \| | N | N | U Manna Whitneya | 0,970125219 |
| \| Cholesterol mg/dl \| \| --- \| | N | N | U Manna Whitneya | 0,00321762484 |
| \| Triglicerydy mg/dl \| \| --- \| | T | N | U Manna Whitneya | 0,508203051 |
| \| HDL mg/dl \| \| --- \| | T | N | U Manna Whitneya | 0,812508492 |
| \| Pt % \| \| --- \| | T | T | T Studenta | 0,0392579079 |
| \| Białko całkowite g/dl \| \| --- \| | N | T | U Manna Whitneya | 0,955200918 |
| \| Albumina g/dl \| \| --- \| | T | T | T Studenta | 0,842302904 |
| \| APF ng/ml \| \| --- \| | N | N | U Manna Whitneya | 0,0838100307 |
| \| CEA ng/ml \| \| --- \| | N | N | U Manna Whitneya | 0,271956278 |
| \| CA 19,9 U/ml \| \| --- \| | N | N | U Manna Whitneya | 0,124661567 |
| \| BMI \| \| --- \| | T | N | U Manna Whitneya | 0,187827426 |
| \| Obwód pasa cm \| \| --- \| | T | T | T Studenta | 0,347278748 |
| \| HOMA-IR \| \| --- \| | T | N | U Manna Whitneya | 0,0000000000000000 |

1. Nadciśnienie tak nie

| Zmienna | Nadciśnienie tętnicze=Tak | | | | | | |
| --- | --- | --- | --- | --- | --- | --- | --- |
|  | \| Nważnych \| \| --- \| | \| Średnia \| \| --- \| | \| Mediana \| \| --- \| | \| Minimum \| \| --- \| | \| Maksimum \| \| --- \| | \| Kwartyl. Rozstęp \| \| --- \| | \| Odch.std \| \| --- \| |
| \| Waspina [ng/mL] \| \| --- \| | 19 | 0,43 | 0,14 | 0,029 | 1,9 | 0,59 | 0,6 |
| \| Wisfatyna [ng/mL] \| \| --- \| | 20 | 4,47 | 4,81 | 0,517 | 8,8 | 3,19 | 2,1 |
| \| Iryzyna [µg/mL] \| \| --- \| | 20 | 2,72 | 2,55 | 0,819 | 4,5 | 1,85 | 1,2 |
| \| ESM-1 [ng/mL] \| \| --- \| | 20 | 24,39 | 20,73 | 18,517 | 55,8 | 3,33 | 10,8 |
| \| Betatrofina [ng/mL] \| \| --- \| | 19 | 26,68 | 24,44 | 10,960 | 49,9 | 17,47 | 12,0 |
| \| Insulina [ng/mL] \| \| --- \| | 20 | 1,20 | 1,06 | 0,308 | 3,8 | 0,85 | 0,9 |
| \| Insulina [mIU/mL] \| \| --- \| | 20 | 27,73 | 24,38 | 7,117 | 87,0 | 19,68 | 21,8 |
| \| Wiek( lata) \| \| --- \| | 20 | 63,50 | 64,00 | 43,000 | 85,0 | 17,00 | 11,5 |
| \| WBC 106/ul \| \| --- \| | 20 | 5,32 | 4,51 | 1,780 | 10,4 | 4,95 | 2,8 |
| \| HGB mg/dl \| \| --- \| | 20 | 12,37 | 12,55 | 8,900 | 16,5 | 2,20 | 2,1 |
| \| PLT 106/ul \| \| --- \| | 20 | 127,30 | 90,50 | 18,000 | 392,0 | 120,00 | 101,2 |
| \| ALT IU/L \| \| --- \| | 20 | 42,25 | 37,00 | 14,000 | 150,0 | 19,50 | 28,6 |
| \| AST IU/L \| \| --- \| | 20 | 52,30 | 49,00 | 16,000 | 105,0 | 24,50 | 24,7 |
| \| FA IU/L \| \| --- \| | 20 | 137,60 | 94,00 | 55,000 | 476,0 | 92,00 | 99,7 |
| \| GGTP Iu/L \| \| --- \| | 20 | 215,45 | 105,00 | 21,000 | 1243,0 | 143,00 | 324,4 |
| \| Glukoza mg/dl \| \| --- \| | 20 | 120,24 | 104,30 | 81,200 | 242,9 | 56,80 | 46,7 |
| \| Mocznik mg/dl \| \| --- \| | 20 | 38,26 | 34,95 | 21,400 | 74,1 | 9,70 | 13,2 |
| \| Kreatynina mg/dL \| \| --- \| | 20 | 0,84 | 0,80 | 0,620 | 1,3 | 0,20 | 0,2 |
| \| Bilirubina mg/dl \| \| --- \| | 20 | 9,55 | 1,25 | 0,430 | 152,1 | 1,07 | 33,6 |
| \| Cholesterol mg/dl \| \| --- \| | 20 | 156,39 | 152,80 | 69,500 | 246,8 | 68,55 | 48,1 |
| \| Triglicerydy mg/dl \| \| --- \| | 20 | 121,82 | 128,85 | 40,000 | 228,0 | 63,10 | 49,0 |
| \| HDL mg/dl \| \| --- \| | 20 | 37,07 | 34,50 | 15,000 | 55,0 | 18,05 | 11,0 |
| \| Pt % \| \| --- \| | 20 | 77,65 | 78,00 | 55,000 | 101,0 | 17,45 | 12,6 |
| \| Białko całkowite g/dl \| \| --- \| | 20 | 7,45 | 7,20 | 5,500 | 9,8 | 0,85 | 1,1 |
| \| Albumina g/dl \| \| --- \| | 20 | 3,32 | 3,35 | 1,900 | 4,4 | 0,90 | 0,6 |
| \| APF ng/ml \| \| --- \| | 20 | 712,95 | 34,34 | 1,010 | 5845,0 | 193,32 | 1543,1 |
| \| CEA ng/ml \| \| --- \| | 20 | 3,33 | 2,18 | 0,165 | 18,9 | 1,59 | 4,0 |
| \| CA 19,9 U/ml \| \| --- \| | 20 | 22,54 | 18,77 | 2,830 | 56,4 | 20,25 | 15,8 |
| \| BMI \| \| --- \| | 20 | 30,40 | 29,50 | 22,000 | 38,0 | 6,00 | 4,4 |
| \| Obwód pasa cm \| \| --- \| | 20 | 105,50 | 108,50 | 70,000 | 130,0 | 20,50 | 14,8 |
| \| HOMA-IR \| \| --- \| | 20 | 8,64 | 6,34 | 1,636 | 24,8 | 13,73 | 7,3 |

| Zmienna | Nadciśnienie tętnicze=Nie | | | | | | |
| --- | --- | --- | --- | --- | --- | --- | --- |
|  | \| Nważnych \| \| --- \| | \| Średnia \| \| --- \| | \| Mediana \| \| --- \| | \| Minimum \| \| --- \| | \| Maksimum \| \| --- \| | \| Kwartyl. Rozstęp \| \| --- \| | \| Odch.std \| \| --- \| |
| \| Waspina [ng/mL] \| \| --- \| | 47 | 0,55 | 0,20 | 0,030 | 5,2 | 0,451 | 1,05 |
| \| Wisfatyna [ng/mL] \| \| --- \| | 46 | 4,16 | 4,46 | 0,819 | 7,5 | 2,739 | 1,66 |
| \| Iryzyna [µg/mL] \| \| --- \| | 49 | 2,73 | 2,52 | 0,411 | 4,8 | 1,640 | 1,14 |
| \| ESM-1 [ng/mL] \| \| --- \| | 47 | 22,54 | 20,22 | 16,120 | 60,0 | 4,997 | 7,23 |
| \| Betatrofina [ng/mL] \| \| --- \| | 46 | 35,65 | 41,21 | 10,895 | 50,0 | 25,278 | 13,15 |
| \| Insulina [ng/mL] \| \| --- \| | 48 | 1,16 | 0,86 | 0,231 | 3,9 | 0,850 | 0,90 |
| \| Insulina [mIU/mL] \| \| --- \| | 49 | 26,35 | 19,73 | 5,223 | 90,9 | 19,600 | 20,75 |
| \| Wiek( lata) \| \| --- \| | 49 | 58,86 | 59,00 | 20,000 | 88,0 | 9,000 | 12,21 |
| \| WBC 106/ul \| \| --- \| | 48 | 4,87 | 4,30 | 1,860 | 10,0 | 3,200 | 2,33 |
| \| HGB mg/dl \| \| --- \| | 49 | 11,56 | 12,00 | 3,160 | 16,0 | 4,000 | 2,55 |
| \| PLT 106/ul \| \| --- \| | 48 | 109,44 | 88,50 | 35,000 | 389,0 | 67,000 | 77,91 |
| \| ALT IU/L \| \| --- \| | 49 | 68,20 | 60,00 | 15,000 | 303,0 | 62,000 | 53,02 |
| \| AST IU/L \| \| --- \| | 49 | 95,45 | 76,00 | 20,000 | 409,0 | 96,000 | 72,16 |
| \| FA IU/L \| \| --- \| | 49 | 145,27 | 111,00 | 38,000 | 427,0 | 67,000 | 85,88 |
| \| GGTP Iu/L \| \| --- \| | 49 | 123,10 | 91,00 | 26,000 | 427,0 | 92,000 | 97,35 |
| \| Glukoza mg/dl \| \| --- \| | 49 | 120,77 | 106,80 | 56,700 | 281,0 | 39,600 | 43,25 |
| \| Mocznik mg/dl \| \| --- \| | 49 | 43,14 | 35,30 | 14,700 | 229,3 | 21,100 | 35,20 |
| \| Kreatynina mg/dL \| \| --- \| | 49 | 1,61 | 0,75 | 0,490 | 37,4 | 0,240 | 5,23 |
| \| Bilirubina mg/dl \| \| --- \| | 49 | 2,25 | 1,74 | 0,480 | 7,4 | 1,720 | 1,51 |
| \| Cholesterol mg/dl \| \| --- \| | 49 | 151,81 | 144,30 | 2,020 | 374,0 | 42,800 | 51,33 |
| \| Triglicerydy mg/dl \| \| --- \| | 49 | 114,60 | 101,00 | 48,900 | 676,1 | 47,800 | 88,30 |
| \| HDL mg/dl \| \| --- \| | 49 | 44,24 | 38,30 | 7,800 | 235,8 | 16,700 | 36,08 |
| \| Pt % \| \| --- \| | 49 | 73,01 | 71,00 | 40,000 | 102,8 | 18,000 | 14,60 |
| \| Białko całkowite g/dl \| \| --- \| | 49 | 7,01 | 7,07 | 4,300 | 8,7 | 1,000 | 0,85 |
| \| Albumina g/dl \| \| --- \| | 49 | 3,18 | 3,10 | 2,000 | 4,6 | 0,600 | 0,57 |
| \| APF ng/ml \| \| --- \| | 49 | 219,12 | 11,22 | 0,740 | 5845,0 | 36,250 | 917,94 |
| \| CEA ng/ml \| \| --- \| | 49 | 3,60 | 3,20 | 0,500 | 12,2 | 2,450 | 2,39 |
| \| CA 19,9 U/ml \| \| --- \| | 49 | 38,19 | 14,91 | 2,000 | 728,7 | 21,790 | 105,47 |
| \| BMI \| \| --- \| | 49 | 29,26 | 28,00 | 22,000 | 45,0 | 5,000 | 4,52 |
| \| Obwód pasa cm \| \| --- \| | 49 | 101,61 | 100,00 | 80,000 | 139,0 | 18,000 | 11,50 |
| \| HOMA-IR \| \| --- \| | 49 | 8,26 | 5,30 | 1,377 | 48,2 | 5,408 | 8,49 |

| ZMIENNA | HA- TAK | HA- NIE | TEST | P |
| --- | --- | --- | --- | --- |
| \| Waspina [ng/mL] \| \| --- \| | N | N | U Manna Whitneya | 0,561479553 |
| \| Wisfatyna [ng/mL] \| \| --- \| | T | T | T Studenta | 0,521430542 |
| \| Iryzyna [µg/mL] \| \| --- \| | T | T | T Studenta | 0,961249232 |
| \| ESM-1 [ng/mL] \| \| --- \| | N | N | U Manna Whitneya | 0,515158358 |
| \| Betatrofina [ng/mL] \| \| --- \| | T | N | U Manna Whitneya | 0,019840946 |
| \| Insulina [ng/mL] \| \| --- \| | N | N | U Manna Whitneya | 0,983892359 |
| \| Insulina [mIU/mL] \| \| --- \| | N | N | U Manna Whitneya | 0,879108936 |
| \| Wiek( lata) \| \| --- \| | T | N | U Manna Whitneya | 0,255356865 |
| \| WBC 106/ul \| \| --- \| | T | N | U Manna Whitneya | 0,7365026 |
| \| HGB mg/dl \| \| --- \| | T | N | U Manna Whitneya | 0,371989009 |
| \| PLT 106/ul \| \| --- \| | N | N | U Manna Whitneya | 0,741582349 |
| \| ALT IU/L \| \| --- \| | N | N | U Manna Whitneya | 0,0332230771 |
| \| AST IU/L \| \| --- \| | T | N | U Manna Whitneya | 0,0152260088 |
| \| FA IU/L \| \| --- \| | N | N | U Manna Whitneya | 0,223684212 |
| \| GGTP Iu/L \| \| --- \| | N | N | U Manna Whitneya | 0,900011425 |
| \| Glukoza mg/dl \| \| --- \| | N | N | U Manna Whitneya | 0,583088833 |
| \| Mocznik mg/dl \| \| --- \| | N | N | U Manna Whitneya | 0,755944484 |
| \| Kreatynina mg/dL \| \| --- \| | N | N | U Manna Whitneya | 0,504189564 |
| \| Bilirubina mg/dl \| \| --- \| | N | N | U Manna Whitneya | 0,179454602 |
| \| Cholesterol mg/dl \| \| --- \| | T | N | U Manna Whitneya | 0,578558528 |
| \| Triglicerydy mg/dl \| \| --- \| | T | N | U Manna Whitneya | 0,145708961 |
| \| HDL mg/dl \| \| --- \| | T | N | U Manna Whitneya | 0,643429158 |
| \| Pt % \| \| --- \| | T | T | T Studenta | 0,21814278 |
| \| Białko całkowite g/dl \| \| --- \| | T | T | T Studenta | 0,0799722847 |
| \| Albumina g/dl \| \| --- \| | T | T | T Studenta | 0,360612023 |
| \| APF ng/ml \| \| --- \| | N | N | U Manna Whitneya | 0,121757649 |
| \| CEA ng/ml \| \| --- \| | N | N | U Manna Whitneya | 0,162913354 |
| \| CA 19,9 U/ml \| \| --- \| | N | N | U Manna Whitneya | 0,454901701 |
| \| BMI \| \| --- \| | T | N | U Manna Whitneya | 0,166936698 |
| \| Obwód pasa cm \| \| --- \| | T | T | T Studenta | 0,246449748 |
| \| HOMA-IR \| \| --- \| | N | N | U Manna Whitneya | 0,973622771 |

1. BCLC – 3 osobno (skala Barcelońska zaawansowania HCC - stopnie A-C)

| Zmienna | BCLC=A | | | | | | |
| --- | --- | --- | --- | --- | --- | --- | --- |
|  | \| Nważnych \| \| --- \| | \| Średnia \| \| --- \| | \| Mediana \| \| --- \| | \| Minimum \| \| --- \| | \| Maksimum \| \| --- \| | \| Kwartyl. Rozstęp \| \| --- \| | \| Odch.std \| \| --- \| |
| \| Waspina [ng/mL] \| \| --- \| | 12 | 0,89 | 0,53 | 0,09 | 5,2 | 0,64 | 1,41 |
| \| Wisfatyna [ng/mL] \| \| --- \| | 12 | 4,58 | 4,67 | 2,48 | 7,5 | 3,78 | 1,89 |
| \| Iryzyna [µg/mL] \| \| --- \| | 12 | 3,30 | 3,40 | 1,32 | 4,7 | 2,65 | 1,30 |
| \| ESM-1 [ng/mL] \| \| --- \| | 12 | 24,29 | 20,52 | 16,12 | 60,0 | 3,66 | 12,05 |
| \| Betatrofina [ng/mL] \| \| --- \| | 12 | 29,28 | 27,91 | 10,90 | 50,0 | 33,02 | 15,46 |
| \| Insulina [ng/mL] \| \| --- \| | 12 | 1,37 | 1,11 | 0,52 | 3,6 | 0,86 | 0,91 |
| \| Insulina [mIU/mL] \| \| --- \| | 12 | 31,67 | 25,69 | 11,90 | 84,1 | 19,94 | 21,02 |
| \| Wiek( lata) \| \| --- \| | 12 | 58,58 | 60,50 | 40,00 | 69,0 | 10,00 | 9,65 |
| \| WBC 106/ul \| \| --- \| | 12 | 6,14 | 6,84 | 1,86 | 10,4 | 5,94 | 3,20 |
| \| HGB mg/dl \| \| --- \| | 12 | 11,85 | 12,20 | 5,70 | 16,1 | 4,15 | 2,90 |
| \| PLT 106/ul \| \| --- \| | 12 | 135,58 | 97,00 | 63,00 | 306,0 | 123,00 | 82,76 |
| \| ALT IU/L \| \| --- \| | 12 | 56,25 | 42,50 | 20,00 | 150,0 | 46,00 | 40,28 |
| \| AST IU/L \| \| --- \| | 12 | 62,00 | 57,50 | 22,00 | 153,0 | 41,50 | 37,48 |
| \| FA IU/L \| \| --- \| | 12 | 118,08 | 103,50 | 77,00 | 257,0 | 44,50 | 49,44 |
| \| GGTP Iu/L \| \| --- \| | 12 | 112,25 | 107,50 | 21,00 | 241,0 | 107,00 | 66,72 |
| \| Glukoza mg/dl \| \| --- \| | 12 | 119,08 | 119,40 | 82,70 | 168,3 | 43,00 | 28,31 |
| \| Mocznik mg/dl \| \| --- \| | 12 | 43,00 | 44,65 | 16,60 | 74,1 | 20,80 | 16,71 |
| \| Kreatynina mg/dL \| \| --- \| | 12 | 0,89 | 0,85 | 0,61 | 1,3 | 0,40 | 0,23 |
| \| Bilirubina mg/dl \| \| --- \| | 12 | 2,11 | 1,64 | 0,95 | 7,4 | 1,08 | 1,75 |
| \| Cholesterol mg/dl \| \| --- \| | 12 | 141,60 | 140,00 | 100,70 | 193,1 | 30,35 | 26,62 |
| \| Triglicerydy mg/dl \| \| --- \| | 12 | 99,83 | 94,40 | 59,00 | 179,1 | 51,15 | 34,38 |
| \| HDL mg/dl \| \| --- \| | 12 | 35,78 | 34,75 | 15,00 | 54,8 | 18,80 | 12,25 |
| \| Pt % \| \| --- \| | 12 | 77,66 | 78,50 | 63,00 | 94,0 | 16,45 | 9,34 |
| \| Białko całkowite g/dl \| \| --- \| | 12 | 7,05 | 7,15 | 6,10 | 7,7 | 0,60 | 0,49 |
| \| Albumina g/dl \| \| --- \| | 12 | 3,30 | 3,10 | 3,00 | 4,1 | 0,35 | 0,35 |
| \| APF ng/ml \| \| --- \| | 12 | 268,98 | 25,23 | 0,74 | 2763,4 | 57,69 | 788,02 |
| \| CEA ng/ml \| \| --- \| | 12 | 3,09 | 2,38 | 0,17 | 8,8 | 2,12 | 2,35 |
| \| CA 19,9 U/ml \| \| --- \| | 12 | 18,37 | 13,14 | 2,00 | 65,6 | 15,01 | 19,62 |
| \| BMI \| \| --- \| | 12 | 29,17 | 29,50 | 22,00 | 35,0 | 6,50 | 4,26 |
| \| Obwód pasa cm \| \| --- \| | 12 | 102,08 | 104,00 | 83,00 | 116,0 | 17,00 | 10,73 |
| \| HOMA-IR \| \| --- \| | 12 | 9,60 | 7,15 | 2,44 | 24,8 | 8,73 | 6,80 |

| Zmienna | BCLC=B | | | | | | |
| --- | --- | --- | --- | --- | --- | --- | --- |
|  | \| Nważnych \| \| --- \| | \| Średnia \| \| --- \| | \| Mediana \| \| --- \| | \| Minimum \| \| --- \| | \| Maksimum \| \| --- \| | \| Kwartyl. Rozstęp \| \| --- \| | \| Odch.std \| \| --- \| |
| \| Waspina [ng/mL] \| \| --- \| | 18 | 0,51 | 0,22 | 0,03 | 1,9 | 0,57 | 0,58 |
| \| Wisfatyna [ng/mL] \| \| --- \| | 19 | 4,26 | 4,43 | 1,14 | 6,2 | 2,59 | 1,59 |
| \| Iryzyna [µg/mL] \| \| --- \| | 20 | 2,54 | 2,52 | 0,82 | 4,5 | 1,38 | 1,00 |
| \| ESM-1 [ng/mL] \| \| --- \| | 20 | 23,15 | 19,82 | 16,93 | 55,2 | 4,92 | 9,19 |
| \| Betatrofina [ng/mL] \| \| --- \| | 19 | 30,12 | 31,60 | 11,39 | 49,4 | 23,46 | 13,08 |
| \| Insulina [ng/mL] \| \| --- \| | 20 | 1,01 | 0,81 | 0,31 | 3,9 | 0,71 | 0,90 |
| \| Insulina [mIU/mL] \| \| --- \| | 20 | 23,42 | 18,77 | 7,12 | 90,9 | 16,30 | 20,74 |
| \| Wiek( lata) \| \| --- \| | 20 | 66,00 | 64,00 | 49,00 | 88,0 | 15,50 | 11,57 |
| \| WBC 106/ul \| \| --- \| | 20 | 5,08 | 4,43 | 1,95 | 9,3 | 2,21 | 1,81 |
| \| HGB mg/dl \| \| --- \| | 20 | 11,99 | 12,25 | 9,20 | 15,1 | 2,65 | 1,72 |
| \| PLT 106/ul \| \| --- \| | 20 | 133,70 | 103,00 | 31,00 | 389,0 | 132,50 | 100,47 |
| \| ALT IU/L \| \| --- \| | 20 | 82,00 | 60,00 | 20,00 | 303,0 | 51,00 | 68,97 |
| \| AST IU/L \| \| --- \| | 20 | 107,50 | 89,00 | 19,00 | 409,0 | 94,00 | 86,15 |
| \| FA IU/L \| \| --- \| | 20 | 147,60 | 116,00 | 69,00 | 476,0 | 79,50 | 93,47 |
| \| GGTP Iu/L \| \| --- \| | 20 | 170,35 | 102,00 | 44,00 | 1243,0 | 73,00 | 261,95 |
| \| Glukoza mg/dl \| \| --- \| | 20 | 126,38 | 106,50 | 76,40 | 267,9 | 42,15 | 50,35 |
| \| Mocznik mg/dl \| \| --- \| | 20 | 37,68 | 36,05 | 19,10 | 72,4 | 10,30 | 11,65 |
| \| Kreatynina mg/dL \| \| --- \| | 20 | 0,80 | 0,78 | 0,54 | 1,3 | 0,14 | 0,17 |
| \| Bilirubina mg/dl \| \| --- \| | 20 | 2,42 | 1,68 | 0,60 | 9,7 | 2,07 | 2,18 |
| \| Cholesterol mg/dl \| \| --- \| | 20 | 173,05 | 152,90 | 117,70 | 374,0 | 53,60 | 59,23 |
| \| Triglicerydy mg/dl \| \| --- \| | 20 | 117,29 | 119,70 | 54,00 | 180,0 | 55,30 | 36,04 |
| \| HDL mg/dl \| \| --- \| | 20 | 38,05 | 37,00 | 12,20 | 55,0 | 13,85 | 11,38 |
| \| Pt % \| \| --- \| | 20 | 76,64 | 74,05 | 55,00 | 102,8 | 14,00 | 12,54 |
| \| Białko całkowite g/dl \| \| --- \| | 20 | 7,10 | 7,25 | 4,30 | 8,7 | 1,20 | 1,05 |
| \| Albumina g/dl \| \| --- \| | 20 | 3,19 | 3,25 | 2,00 | 4,5 | 0,95 | 0,69 |
| \| APF ng/ml \| \| --- \| | 20 | 359,25 | 38,35 | 4,22 | 3340,0 | 89,82 | 940,89 |
| \| CEA ng/ml \| \| --- \| | 20 | 3,57 | 2,74 | 0,82 | 18,9 | 2,06 | 3,79 |
| \| CA 19,9 U/ml \| \| --- \| | 20 | 57,19 | 19,54 | 2,83 | 728,7 | 24,77 | 158,79 |
| \| BMI \| \| --- \| | 20 | 29,80 | 28,50 | 23,00 | 38,0 | 4,00 | 3,72 |
| \| Obwód pasa cm \| \| --- \| | 20 | 104,45 | 103,00 | 88,00 | 130,0 | 20,00 | 12,60 |
| \| HOMA-IR \| \| --- \| | 20 | 7,59 | 4,98 | 1,86 | 25,7 | 6,55 | 7,22 |

| Zmienna | BCLC=C | | | | | | |
| --- | --- | --- | --- | --- | --- | --- | --- |
|  | \| Nważnych \| \| --- \| | \| Średnia \| \| --- \| | \| Mediana \| \| --- \| | \| Minimum \| \| --- \| | \| Maksimum \| \| --- \| | \| Kwartyl. Rozstęp \| \| --- \| | \| Odch.std \| \| --- \| |
| \| Waspina [ng/mL] \| \| --- \| | 16 | 0,70 | 0,19 | 0,032 | 4,9 | 0,49 | 1,2 |
| \| Wisfatyna [ng/mL] \| \| --- \| | 16 | 4,35 | 4,77 | 0,517 | 8,8 | 3,13 | 2,2 |
| \| Iryzyna [µg/mL] \| \| --- \| | 16 | 2,11 | 1,89 | 0,411 | 4,3 | 1,14 | 1,1 |
| \| ESM-1 [ng/mL] \| \| --- \| | 15 | 21,16 | 20,88 | 17,683 | 28,4 | 4,52 | 2,8 |
| \| Betatrofina [ng/mL] \| \| --- \| | 15 | 37,29 | 43,42 | 16,825 | 50,0 | 24,94 | 12,6 |
| \| Insulina [ng/mL] \| \| --- \| | 16 | 1,19 | 0,89 | 0,419 | 3,8 | 0,77 | 1,0 |
| \| Insulina [mIU/mL] \| \| --- \| | 16 | 27,57 | 20,58 | 9,678 | 87,0 | 17,71 | 22,0 |
| \| Wiek( lata) \| \| --- \| | 16 | 57,69 | 59,00 | 20,000 | 77,0 | 10,00 | 13,3 |
| \| WBC 106/ul \| \| --- \| | 16 | 4,80 | 3,61 | 1,780 | 10,0 | 3,89 | 2,8 |
| \| HGB mg/dl \| \| --- \| | 16 | 11,57 | 11,15 | 8,300 | 15,0 | 4,00 | 2,2 |
| \| PLT 106/ul \| \| --- \| | 16 | 99,25 | 76,00 | 18,000 | 392,0 | 64,50 | 89,5 |
| \| ALT IU/L \| \| --- \| | 16 | 63,63 | 61,00 | 14,000 | 149,0 | 48,00 | 34,3 |
| \| AST IU/L \| \| --- \| | 16 | 98,88 | 97,50 | 16,000 | 202,0 | 88,50 | 54,7 |
| \| FA IU/L \| \| --- \| | 16 | 170,88 | 127,50 | 55,000 | 427,0 | 130,00 | 113,2 |
| \| GGTP Iu/L \| \| --- \| | 16 | 157,94 | 108,50 | 28,000 | 482,0 | 174,00 | 133,4 |
| \| Glukoza mg/dl \| \| --- \| | 16 | 110,15 | 95,45 | 79,100 | 242,9 | 32,65 | 42,1 |
| \| Mocznik mg/dl \| \| --- \| | 16 | 41,35 | 34,90 | 18,600 | 139,1 | 13,85 | 29,5 |
| \| Kreatynina mg/dL \| \| --- \| | 16 | 0,87 | 0,70 | 0,490 | 2,2 | 0,21 | 0,5 |
| \| Bilirubina mg/dl \| \| --- \| | 16 | 2,18 | 1,43 | 0,430 | 6,4 | 1,86 | 1,7 |
| \| Cholesterol mg/dl \| \| --- \| | 16 | 157,09 | 157,05 | 87,000 | 233,0 | 59,95 | 44,5 |
| \| Triglicerydy mg/dl \| \| --- \| | 16 | 108,45 | 109,00 | 40,000 | 170,0 | 48,30 | 35,9 |
| \| HDL mg/dl \| \| --- \| | 16 | 37,08 | 35,95 | 23,000 | 55,0 | 14,65 | 9,7 |
| \| Pt % \| \| --- \| | 16 | 73,37 | 71,50 | 40,000 | 100,0 | 19,95 | 16,3 |
| \| Białko całkowite g/dl \| \| --- \| | 16 | 7,30 | 7,07 | 5,500 | 9,8 | 1,80 | 1,3 |
| \| Albumina g/dl \| \| --- \| | 16 | 2,96 | 2,90 | 1,900 | 3,8 | 0,80 | 0,5 |
| \| APF ng/ml \| \| --- \| | 16 | 902,36 | 37,78 | 2,090 | 5845,0 | 374,86 | 1967,6 |
| \| CEA ng/ml \| \| --- \| | 16 | 3,45 | 2,84 | 0,860 | 8,8 | 2,99 | 2,2 |
| \| CA 19,9 U/ml \| \| --- \| | 16 | 25,23 | 21,30 | 2,000 | 78,3 | 32,44 | 21,6 |
| \| BMI \| \| --- \| | 16 | 30,38 | 29,50 | 22,000 | 45,0 | 5,50 | 5,8 |
| \| Obwód pasa cm \| \| --- \| | 16 | 104,38 | 107,00 | 70,000 | 139,0 | 18,50 | 16,5 |
| \| HOMA-IR \| \| --- \| | 16 | 7,26 | 5,37 | 1,962 | 17,9 | 5,19 | 5,4 |

| ZMIENNA | BLC-A | BLC-B | BLC-C | Test | p |
| --- | --- | --- | --- | --- | --- |
| \| Waspina [ng/mL] \| \| --- \| | N | N | N | Kruskal- Wallis | 0, 5299 |
| \| Wisfatyna [ng/mL] \| \| --- \| | T | T | T | ANOVA | 0,898343473 |
| \| Iryzyna [µg/mL] \| \| --- \| | T | T | T | ANOVA | 0,0261508165 |
| \| ESM-1 [ng/mL] \| \| --- \| | N | N | T | Kruskal- Wallis | 0, 9645 |
| \| Betatrofina [ng/mL] \| \| --- \| | T | T | N | Kruskal- Wallis | 0, 2111 |
| \| Insulina [ng/mL] \| \| --- \| | N | N | N | Kruskal- Wallis | 0, 1606 |
| \| Insulina [mIU/mL] \| \| --- \| | N | N | N | Kruskal- Wallis | 0, 1606 |
| \| Wiek( lata) \| \| --- \| | T | T | N | Kruskal- Wallis | 0, 2829 |
| \| WBC 106/ul \| \| --- \| | T | T | N | Kruskal- Wallis | 0, 3922 |
| \| HGB mg/dl \| \| --- \| | T | T | T | ANOVA | 0,850561602 |
| \| PLT 106/ul \| \| --- \| | N | N | N | Kruskal- Wallis | 0, 2989 |
| \| ALT IU/L \| \| --- \| | N | N | T | Kruskal- Wallis | 0, 3648 |
| \| AST IU/L \| \| --- \| | T | N | T | Kruskal- Wallis | 0, 1164 |
| \| FA IU/L \| \| --- \| | N | N | N | Kruskal- Wallis | 0, 5393 |
| \| GGTP Iu/L \| \| --- \| | T | N | N | Kruskal- Wallis | 0, 9050 |
| \| Glukoza mg/dl \| \| --- \| | T | N | N | Kruskal- Wallis | 0, 2495 |
| \| Mocznik mg/dl \| \| --- \| | T | N | N | Kruskal- Wallis | 0, 3735 |
| \| Kreatynina mg/dL \| \| --- \| | T | T | N | Kruskal- Wallis | 0, 3429 |
| \| Bilirubina mg/dl \| \| --- \| | N | N | N | Kruskal- Wallis | 0, 9934 |
| \| Cholesterol mg/dl \| \| --- \| | T | N | T | Kruskal- Wallis | 0, 2825 |
| \| Triglicerydy mg/dl \| \| --- \| | T | T | T | ANOVA | 0,403972569 |
| \| HDL mg/dl \| \| --- \| | T | T | T | ANOVA | 0,853787559 |
| \| Pt % \| \| --- \| | T | T | T | ANOVA | 0,655397215 |
| \| Białko całkowite g/dl \| \| --- \| | T | T | T | ANOVA | 0,777953275 |
| \| Albumina g/dl \| \| --- \| | N | T | T | Kruskal- Wallis | 0, 1911 |
| \| APF ng/ml \| \| --- \| | N | N | N | Kruskal- Wallis | 0, 4894 |
| \| CEA ng/ml \| \| --- \| | T | N | T | Kruskal- Wallis | 0, 8237 |
| \| CA 19,9 U/ml \| \| --- \| | N | N | T | Kruskal- Wallis | 0, 4155 |
| \| BMI \| \| --- \| | T | T | T | ANOVA | 0,792370052 |
| \| Obwód pasa cm \| \| --- \| | T | T | T | ANOVA | 0,877101715 |
| \| HOMA-IR \| \| --- \| | T | N | N | Kruskal- Wallis | 0, 3615 |

TESTY POST HOC dla jedynej zmiennej znamiennej z poprzedniej tabeli

| BCLC | Test RIR Tukeya; zmienna: Iryzyna [µg/mL] | | |
| --- | --- | --- | --- |
|  | \| {1} M=3,2972 \| \| --- \| | \| {2} M=2,5409 \| \| --- \| | \| {3} M=2,1058 \| \| --- \| |
| \| A {1} \| \| --- \| |  | 0,162052 | 0,020086 |
| \| B {2} \| \| --- \| | 0,162052 |  | 0,479579 |
| \| C {3} \| \| --- \| | 0,020086 | 0,479579 |  |


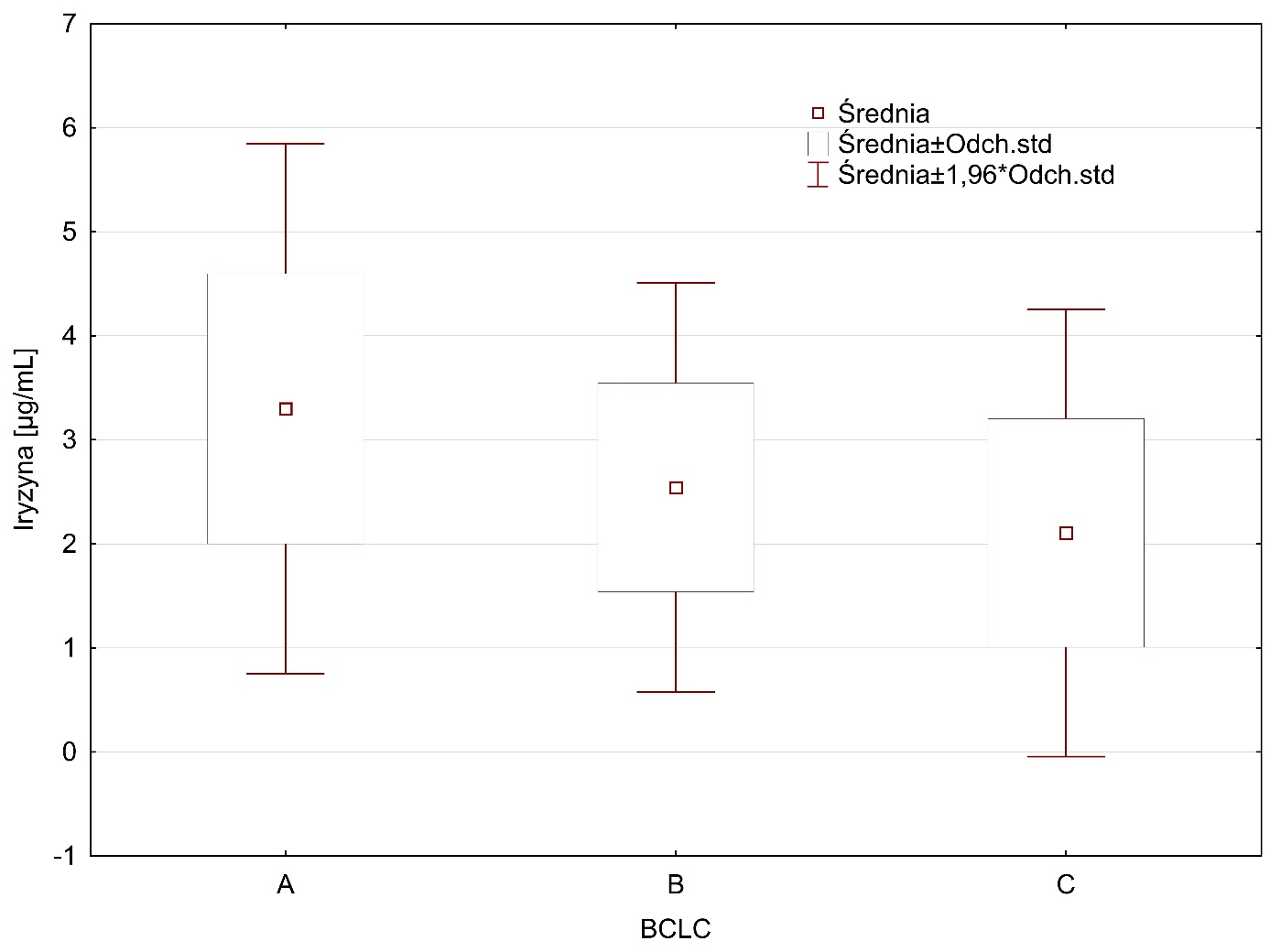


1. CHP osobno ABC i osobno punkty

| Zmienna | CHP=A | | | | | | |
| --- | --- | --- | --- | --- | --- | --- | --- |
|  | \| Nważnych \| \| --- \| | \| Średnia \| \| --- \| | \| Mediana \| \| --- \| | \| Minimum \| \| --- \| | \| Maksimum \| \| --- \| | \| Kwartyl. Rozstęp \| \| --- \| | \| Odch.std \| \| --- \| |
| \| Waspina [ng/mL] \| \| --- \| | 33 | 0,40 | 0,17 | 0,032 | 5,2 | 0,44 | 0,9 |
| \| Wisfatyna [ng/mL] \| \| --- \| | 34 | 4,60 | 4,63 | 0,517 | 7,5 | 2,84 | 1,6 |
| \| Iryzyna [µg/mL] \| \| --- \| | 36 | 3,13 | 3,40 | 0,893 | 4,8 | 2,50 | 1,2 |
| \| ESM-1 [ng/mL] \| \| --- \| | 36 | 23,52 | 20,52 | 16,120 | 60,0 | 4,43 | 9,2 |
| \| Betatrofina [ng/mL] \| \| --- \| | 36 | 29,02 | 24,12 | 10,895 | 50,0 | 24,27 | 13,8 |
| \| Insulina [ng/mL] \| \| --- \| | 35 | 1,08 | 0,79 | 0,231 | 3,8 | 0,80 | 0,9 |
| \| Insulina [mIU/mL] \| \| --- \| | 36 | 24,34 | 16,60 | 5,223 | 87,0 | 16,92 | 20,9 |
| \| Wiek( lata) \| \| --- \| | 36 | 58,44 | 59,00 | 24,000 | 84,0 | 9,50 | 11,2 |
| \| WBC 106/ul \| \| --- \| | 36 | 5,59 | 4,91 | 1,780 | 10,4 | 5,07 | 2,7 |
| \| HGB mg/dl \| \| --- \| | 36 | 12,33 | 12,35 | 5,700 | 16,5 | 3,80 | 2,4 |
| \| PLT 106/ul \| \| --- \| | 36 | 133,53 | 103,00 | 34,000 | 392,0 | 106,00 | 94,9 |
| \| ALT IU/L \| \| --- \| | 36 | 66,81 | 45,50 | 14,000 | 303,0 | 53,00 | 59,0 |
| \| AST IU/L \| \| --- \| | 36 | 79,08 | 61,00 | 16,000 | 409,0 | 69,00 | 70,1 |
| \| FA IU/L \| \| --- \| | 36 | 130,39 | 103,00 | 38,000 | 476,0 | 56,50 | 85,7 |
| \| GGTP Iu/L \| \| --- \| | 36 | 178,47 | 107,00 | 23,000 | 1243,0 | 107,00 | 248,4 |
| \| Glukoza mg/dl \| \| --- \| | 36 | 118,73 | 106,80 | 76,400 | 267,9 | 32,25 | 40,2 |
| \| Mocznik mg/dl \| \| --- \| | 36 | 38,01 | 35,35 | 14,700 | 109,0 | 20,05 | 17,1 |
| \| Kreatynina mg/dL \| \| --- \| | 36 | 0,81 | 0,76 | 0,540 | 2,0 | 0,24 | 0,2 |
| \| Bilirubina mg/dl \| \| --- \| | 36 | 1,61 | 1,29 | 0,430 | 7,4 | 0,78 | 1,3 |
| \| Cholesterol mg/dl \| \| --- \| | 36 | 154,30 | 150,50 | 85,000 | 220,0 | 35,45 | 34,1 |
| \| Triglicerydy mg/dl \| \| --- \| | 36 | 108,96 | 105,20 | 40,000 | 228,0 | 57,25 | 41,8 |
| \| HDL mg/dl \| \| --- \| | 36 | 46,81 | 42,95 | 22,700 | 235,8 | 18,65 | 33,9 |
| \| Pt % \| \| --- \| | 36 | 80,43 | 80,50 | 44,000 | 102,8 | 15,45 | 12,5 |
| \| Białko całkowite g/dl \| \| --- \| | 36 | 7,22 | 7,15 | 4,300 | 9,8 | 0,85 | 1,0 |
| \| Albumina g/dl \| \| --- \| | 36 | 3,46 | 3,40 | 2,000 | 4,6 | 0,75 | 0,6 |
| \| APF ng/ml \| \| --- \| | 36 | 344,17 | 10,11 | 0,740 | 5845,0 | 36,12 | 1142,6 |
| \| CEA ng/ml \| \| --- \| | 36 | 3,69 | 3,11 | 0,165 | 12,2 | 3,06 | 2,7 |
| \| CA 19,9 U/ml \| \| --- \| | 36 | 39,09 | 14,80 | 2,000 | 728,7 | 14,96 | 119,1 |
| \| BMI \| \| --- \| | 36 | 28,58 | 28,00 | 22,000 | 35,0 | 6,00 | 3,9 |
| \| Obwód pasa cm \| \| --- \| | 36 | 100,19 | 98,50 | 70,000 | 130,0 | 15,50 | 11,7 |
| \| HOMA-IR \| \| --- \| | 36 | 7,37 | 4,91 | 1,377 | 25,7 | 6,29 | 6,9 |

| Zmienna | CHP=B Statystyki opisowe (StatPodstawa) | | | | | | |
| --- | --- | --- | --- | --- | --- | --- | --- |
|  | \| Nważnych \| \| --- \| | \| Średnia \| \| --- \| | \| Mediana \| \| --- \| | \| Minimum \| \| --- \| | \| Maksimum \| \| --- \| | \| Kwartyl. Rozstęp \| \| --- \| | \| Odch.std \| \| --- \| |
| \| Waspina [ng/mL] \| \| --- \| | 27 | 0,56 | 0,26 | 0,029 | 2,1 | 1,05 | 0,6 |
| \| Wisfatyna [ng/mL] \| \| --- \| | 26 | 3,69 | 3,26 | 0,819 | 8,8 | 3,04 | 2,0 |
| \| Iryzyna [µg/mL] \| \| --- \| | 27 | 2,29 | 2,42 | 0,411 | 3,7 | 0,95 | 0,8 |
| \| ESM-1 [ng/mL] \| \| --- \| | 26 | 22,65 | 19,78 | 16,933 | 55,2 | 4,67 | 8,1 |
| \| Betatrofina [ng/mL] \| \| --- \| | 24 | 37,07 | 39,27 | 12,732 | 50,0 | 20,49 | 11,4 |
| \| Insulina [ng/mL] \| \| --- \| | 27 | 1,22 | 1,04 | 0,308 | 3,9 | 0,80 | 0,9 |
| \| Insulina [mIU/mL] \| \| --- \| | 27 | 28,15 | 23,94 | 7,117 | 90,9 | 18,58 | 20,0 |
| \| Wiek( lata) \| \| --- \| | 27 | 62,81 | 65,00 | 20,000 | 88,0 | 15,00 | 13,9 |
| \| WBC 106/ul \| \| --- \| | 26 | 4,21 | 3,70 | 1,970 | 9,3 | 2,37 | 1,8 |
| \| HGB mg/dl \| \| --- \| | 27 | 11,23 | 10,80 | 3,160 | 16,0 | 3,40 | 2,6 |
| \| PLT 106/ul \| \| --- \| | 26 | 100,08 | 76,50 | 31,000 | 293,0 | 70,00 | 72,6 |
| \| ALT IU/L \| \| --- \| | 27 | 52,52 | 38,00 | 15,000 | 150,0 | 58,00 | 34,8 |
| \| AST IU/L \| \| --- \| | 27 | 83,78 | 63,00 | 19,000 | 258,0 | 93,00 | 59,8 |
| \| FA IU/L \| \| --- \| | 27 | 153,59 | 119,00 | 64,000 | 427,0 | 79,00 | 91,9 |
| \| GGTP Iu/L \| \| --- \| | 27 | 121,59 | 86,00 | 21,000 | 427,0 | 110,00 | 104,7 |
| \| Glukoza mg/dl \| \| --- \| | 27 | 121,06 | 111,10 | 56,700 | 281,0 | 45,30 | 46,7 |
| \| Mocznik mg/dl \| \| --- \| | 27 | 44,54 | 34,50 | 17,200 | 229,3 | 19,30 | 40,2 |
| \| Kreatynina mg/dL \| \| --- \| | 27 | 2,22 | 0,79 | 0,490 | 37,4 | 0,41 | 7,0 |
| \| Bilirubina mg/dl \| \| --- \| | 27 | 8,03 | 1,97 | 0,600 | 152,1 | 2,73 | 28,9 |
| \| Cholesterol mg/dl \| \| --- \| | 27 | 157,76 | 150,00 | 2,020 | 374,0 | 54,30 | 67,8 |
| \| Triglicerydy mg/dl \| \| --- \| | 27 | 131,75 | 101,00 | 46,000 | 676,1 | 57,00 | 115,1 |
| \| HDL mg/dl \| \| --- \| | 27 | 39,66 | 35,50 | 12,200 | 177,6 | 14,60 | 29,1 |
| \| Pt % \| \| --- \| | 27 | 69,48 | 68,00 | 48,000 | 97,0 | 14,00 | 11,3 |
| \| Białko całkowite g/dl \| \| --- \| | 27 | 7,12 | 7,20 | 5,800 | 8,7 | 1,50 | 0,8 |
| \| Albumina g/dl \| \| --- \| | 27 | 3,01 | 3,00 | 2,000 | 3,9 | 0,80 | 0,5 |
| \| APF ng/ml \| \| --- \| | 27 | 464,25 | 26,70 | 2,030 | 5845,0 | 146,00 | 1277,4 |
| \| CEA ng/ml \| \| --- \| | 27 | 3,26 | 2,68 | 0,500 | 18,9 | 1,89 | 3,3 |
| \| CA 19,9 U/ml \| \| --- \| | 27 | 29,37 | 18,10 | 2,000 | 200,0 | 38,34 | 38,6 |
| \| BMI \| \| --- \| | 27 | 30,55 | 30,00 | 22,900 | 38,0 | 6,00 | 4,5 |
| \| Obwód pasa cm \| \| --- \| | 27 | 104,41 | 107,00 | 82,000 | 127,0 | 21,00 | 12,0 |
| \| HOMA-IR \| \| --- \| | 27 | 9,14 | 6,17 | 1,858 | 48,2 | 9,01 | 9,6 |

| Zmienna | CHP=C Statystyki opisowe (StatPodstawa) | | | | | | |
| --- | --- | --- | --- | --- | --- | --- | --- |
|  | \| Nważnych \| \| --- \| | \| Średnia \| \| --- \| | \| Mediana \| \| --- \| | \| Minimum \| \| --- \| | \| Maksimum \| \| --- \| | \| Kwartyl. Rozstęp \| \| --- \| | \| Odch.std \| \| --- \| |
| \| Waspina [ng/mL] \| \| --- \| | 6 | 0,96 | 0,17 | 0,066 | 4,88 | 0,25 | 1,92 |
| \| Wisfatyna [ng/mL] \| \| --- \| | 6 | 4,74 | 5,34 | 2,213 | 5,91 | 1,29 | 1,37 |
| \| Iryzyna [µg/mL] \| \| --- \| | 6 | 2,27 | 1,91 | 0,889 | 4,30 | 1,24 | 1,19 |
| \| ESM-1 [ng/mL] \| \| --- \| | 5 | 22,32 | 23,38 | 18,517 | 25,53 | 2,77 | 2,73 |
| \| Betatrofina [ng/mL] \| \| --- \| | 5 | 42,44 | 46,17 | 26,093 | 49,90 | 3,17 | 9,42 |
| \| Insulina [ng/mL] \| \| --- \| | 6 | 1,51 | 1,08 | 0,418 | 3,11 | 2,04 | 1,13 |
| \| Insulina [mIU/mL] \| \| --- \| | 6 | 34,92 | 25,02 | 9,655 | 71,83 | 47,20 | 26,04 |
| \| Wiek( lata) \| \| --- \| | 6 | 59,00 | 58,50 | 48,000 | 69,00 | 6,00 | 6,96 |
| \| WBC 106/ul \| \| --- \| | 6 | 4,93 | 4,28 | 2,140 | 9,95 | 1,56 | 2,69 |
| \| HGB mg/dl \| \| --- \| | 6 | 11,17 | 10,65 | 9,020 | 14,50 | 2,20 | 1,96 |
| \| PLT 106/ul \| \| --- \| | 6 | 65,00 | 72,00 | 18,000 | 93,00 | 47,00 | 29,45 |
| \| ALT IU/L \| \| --- \| | 6 | 60,67 | 65,00 | 29,000 | 101,00 | 36,00 | 26,36 |
| \| AST IU/L \| \| --- \| | 6 | 102,33 | 90,50 | 31,000 | 202,00 | 92,00 | 63,19 |
| \| FA IU/L \| \| --- \| | 6 | 171,50 | 156,50 | 55,000 | 352,00 | 111,00 | 103,37 |
| \| GGTP Iu/L \| \| --- \| | 6 | 105,50 | 49,00 | 26,000 | 320,00 | 127,00 | 115,80 |
| \| Glukoza mg/dl \| \| --- \| | 6 | 129,98 | 110,95 | 90,900 | 242,90 | 42,20 | 58,41 |
| \| Mocznik mg/dl \| \| --- \| | 6 | 51,30 | 35,75 | 23,500 | 139,10 | 16,70 | 43,64 |
| \| Kreatynina mg/dL \| \| --- \| | 6 | 1,04 | 0,77 | 0,590 | 2,24 | 0,57 | 0,63 |
| \| Bilirubina mg/dl \| \| --- \| | 6 | 4,37 | 4,43 | 2,740 | 6,40 | 1,52 | 1,29 |
| \| Cholesterol mg/dl \| \| --- \| | 6 | 125,37 | 125,10 | 85,100 | 175,00 | 45,30 | 32,41 |
| \| Triglicerydy mg/dl \| \| --- \| | 6 | 95,33 | 104,70 | 48,900 | 127,70 | 40,00 | 28,95 |
| \| HDL mg/dl \| \| --- \| | 6 | 25,53 | 23,45 | 7,800 | 38,10 | 14,40 | 11,21 |
| \| Pt % \| \| --- \| | 6 | 59,83 | 63,00 | 40,000 | 83,00 | 30,00 | 17,12 |
| \| Białko całkowite g/dl \| \| --- \| | 6 | 6,76 | 6,99 | 5,400 | 8,50 | 1,70 | 1,16 |
| \| Albumina g/dl \| \| --- \| | 6 | 2,73 | 2,80 | 1,900 | 3,30 | 0,40 | 0,48 |
| \| APF ng/ml \| \| --- \| | 6 | 11,90 | 5,18 | 2,090 | 35,48 | 15,21 | 13,11 |
| \| CEA ng/ml \| \| --- \| | 6 | 3,70 | 3,96 | 0,700 | 6,20 | 4,05 | 2,40 |
| \| CA 19,9 U/ml \| \| --- \| | 6 | 20,29 | 10,90 | 2,830 | 78,34 | 13,13 | 28,96 |
| \| BMI \| \| --- \| | 6 | 31,33 | 29,00 | 27,000 | 45,00 | 2,00 | 6,77 |
| \| Obwód pasa cm \| \| --- \| | 6 | 110,50 | 110,00 | 86,000 | 139,00 | 14,00 | 17,41 |
| \| HOMA-IR \| \| --- \| | 6 | 10,92 | 10,67 | 3,176 | 19,66 | 14,45 | 7,95 |

| ZMIENNA | CHP-A | CHP-B | CHP-C | Test | p |
| --- | --- | --- | --- | --- | --- |
| \| Waspina [ng/mL] \| \| --- \| | N | N | N | Kruskal- Wallis | 0, 5858 |
| \| Wisfatyna [ng/mL] \| \| --- \| | T | T | T | ANOVA | 0,114560013 |
| \| Iryzyna [µg/mL] \| \| --- \| | N | T | T | Kruskal- Wallis | 0, 0268 |
| \| ESM-1 [ng/mL] \| \| --- \| | N | N | T | Kruskal- Wallis | 0, 5672 |
| \| Betatrofina [ng/mL] \| \| --- \| | N | N | N | Kruskal- Wallis | 0, 0292 |
| \| Insulina [ng/mL] \| \| --- \| | N | N | T | Kruskal- Wallis | 0, 3678 |
| \| Insulina [mIU/mL] \| \| --- \| | N | N | T | Kruskal- Wallis | 0, 2890 |
| \| Wiek( lata) \| \| --- \| | T | T | T | ANOVA | 0,359870188 |
| \| WBC 106/ul \| \| --- \| | N | N | T | Kruskal- Wallis | 0, 1507 |
| \| HGB mg/dl \| \| --- \| | T | T | T | ANOVA | 0,171599036 |
| \| PLT 106/ul \| \| --- \| | N | N | T | Kruskal- Wallis | 0, 0596 |
| \| ALT IU/L \| \| --- \| | N | N | T | Kruskal- Wallis | 0, 5276 |
| \| AST IU/L \| \| --- \| | N | N | T | Kruskal- Wallis | 0, 5379 |
| \| FA IU/L \| \| --- \| | N | N | T | Kruskal- Wallis | 0, 2470 |
| \| GGTP Iu/L \| \| --- \| | N | N | N | Kruskal- Wallis | 0, 3439 |
| \| Glukoza mg/dl \| \| --- \| | N | N | N | Kruskal- Wallis | 0, 9845 |
| \| Mocznik mg/dl \| \| --- \| | N | N | N | Kruskal- Wallis | 0, 9465 |
| \| Kreatynina mg/dL \| \| --- \| | N | N | N | Kruskal- Wallis | 0, 5687 |
| \| Bilirubina mg/dl \| \| --- \| | N | N | T | Kruskal- Wallis | 0, 0004 |
| \| Cholesterol mg/dl \| \| --- \| | T | N | T | Kruskal- Wallis | 0, 2381 |
| \| Triglicerydy mg/dl \| \| --- \| | T | N | T | Kruskal- Wallis | 0, 6306 |
| \| HDL mg/dl \| \| --- \| | N | N | T | Kruskal- Wallis | 0, 0044 |
| \| Pt % \| \| --- \| | T | T | T | ANOVA | 0,000121806996 |
| \| Białko całkowite g/dl \| \| --- \| | N | T | T | Kruskal- Wallis | 0, 6089 |
| \| Albumina g/dl \| \| --- \| | T | T | T | ANOVA | 0,000545021748 |
| \| APF ng/ml \| \| --- \| | N | N | N | Kruskal- Wallis | 0, 1516 |
| \| CEA ng/ml \| \| --- \| | N | N | T | Kruskal- Wallis | 0, 6670 |
| \| CA 19,9 U/ml \| \| --- \| | N | N | N | Kruskal- Wallis | 0, 5559 |
| \| BMI \| \| --- \| | T | T | N | Kruskal- Wallis | 0, 2289 |
| \| Obwód pasa cm \| \| --- \| | T | T | T | ANOVA | 0,119375954 |
| \| HOMA-IR \| \| --- \| | N | N | N | Kruskal- Wallis | 0, 3518 |

Test post HOC

| CHP | Test RIR Tukeya; zmienna: Pt % | | |
| --- | --- | --- | --- |
|  | \| {1} M=80,428 \| \| --- \| | \| {2} M=69,481 \| \| --- \| | \| {3} M=59,833 \| \| --- \| |
| \| A {1} \| \| --- \| |  | 0,002944 | 0,001214 |
| \| B {2} \| \| --- \| | 0,002944 |  | 0,208653 |
| \| C {3} \| \| --- \| | 0,001214 | 0,208653 |  |

| CHP | Test RIR Tukeya; zmienna: Albumina g/dl | | |
| --- | --- | --- | --- |
|  | \| {1} M=3,4628 \| \| --- \| | \| {2} M=3,0074 \| \| --- \| | \| {3} M=2,7333 \| \| --- \| |
| \| A {1} \| \| --- \| |  | 0,003688 | 0,007702 |
| \| B {2} \| \| --- \| | 0,003688 |  | 0,491366 |
| \| C {3} \| \| --- \| | 0,007702 | 0,491366 |  |

| Zależna: Iryzyna [µg/mL] | Wartość p dla porównań wielokrotnych (dwustronych); Iryzyna [µg/mL] | | |
| --- | --- | --- | --- |
|  | \| A R:41,208 \| \| --- \| | \| B R:28,500 \| \| --- \| | \| C R:27,000 \| \| --- \| |
| \| A \| \| --- \| |  | 0,038528 | 0,324781 |
| \| B \| \| --- \| | 0,038528 |  | 1,000000 |
| \| C \| \| --- \| | 0,324781 | 1,000000 |  |

| Zależna: Betatrofina [ng/mL] | Wartość p dla porównań wielokrotnych (dwustronych); Betatrofina [ng/mL] | | |
| --- | --- | --- | --- |
|  | \| A R:27,667 \| \| --- \| | \| B R:38,333 \| \| --- \| | \| C R:45,800 \| \| --- \| |
| \| A \| \| --- \| |  | 0,096875 | 0,133458 |
| \| B \| \| --- \| | 0,096875 |  | 1,000000 |
| \| C \| \| --- \| | 0,133458 | 1,000000 |  |

| Zależna: Bilirubina mg/dl | Wartość p dla porównań wielokrotnych (dwustronych); Bilirubina mg/dl | | |
| --- | --- | --- | --- |
|  | \| A R:27,431 \| \| --- \| | \| B R:39,593 \| \| --- \| | \| C R:59,750 \| \| --- \| |
| \| A \| \| --- \| |  | 0,051776 | 0,000777 |
| \| B \| \| --- \| | 0,051776 |  | 0,078015 |
| \| C \| \| --- \| | 0,000777 | 0,078015 |  |

| Zależna: HDL mg/dl | Wartość p dla porównań wielokrotnych (dwustronych); HDL mg/dl | | |
| --- | --- | --- | --- |
|  | \| A R:41,583 \| \| --- \| | \| B R:30,556 \| \| --- \| | \| C R:15,500 \| \| --- \| |
| \| A \| \| --- \| |  | 0,092533 | 0,009584 |
| \| B \| \| --- \| | 0,092533 |  | 0,289116 |
| \| C \| \| --- \| | 0,009584 | 0,289116 |  |

1. Płytki krwi >100 tys i < 100tys

| Zmienna | PLT >100 | | | | | | |
| --- | --- | --- | --- | --- | --- | --- | --- |
|  | \| Nważnych \| \| --- \| | \| Średnia \| \| --- \| | \| Mediana \| \| --- \| | \| Minimum \| \| --- \| | \| Maksimum \| \| --- \| | \| Kwartyl. Rozstęp \| \| --- \| | \| Odch.std \| \| --- \| |
| \| Waspina [ng/mL] \| \| --- \| | 27 | 0,47 | 0,12 | 0,03 | 5,2 | 0,45 | 1,0 |
| \| Wisfatyna [ng/mL] \| \| --- \| | 26 | 5,28 | 5,27 | 2,48 | 8,8 | 1,72 | 1,4 |
| \| Iryzyna [µg/mL] \| \| --- \| | 28 | 2,94 | 2,98 | 0,82 | 4,7 | 1,83 | 1,2 |
| \| ESM-1 [ng/mL] \| \| --- \| | 28 | 23,10 | 20,52 | 16,12 | 55,8 | 4,82 | 8,2 |
| \| Betatrofina [ng/mL] \| \| --- \| | 26 | 25,27 | 21,13 | 10,90 | 50,0 | 19,06 | 12,8 |
| \| Insulina [ng/mL] \| \| --- \| | 28 | 0,85 | 0,54 | 0,23 | 2,7 | 0,48 | 0,7 |
| \| Insulina [mIU/mL] \| \| --- \| | 28 | 19,62 | 12,45 | 5,34 | 63,1 | 11,10 | 15,9 |
| \| Wiek( lata) \| \| --- \| | 28 | 60,96 | 59,50 | 40,00 | 85,0 | 14,00 | 11,1 |
| \| WBC 106/ul \| \| --- \| | 28 | 7,04 | 7,54 | 3,13 | 10,4 | 3,35 | 2,1 |
| \| HGB mg/dl \| \| --- \| | 28 | 12,52 | 12,30 | 9,20 | 16,5 | 4,05 | 2,3 |
| \| PLT 106/ul \| \| --- \| | 28 | 188,89 | 171,50 | 101,00 | 392,0 | 116,00 | 87,1 |
| \| ALT IU/L \| \| --- \| | 28 | 66,25 | 49,00 | 14,00 | 303,0 | 60,50 | 58,3 |
| \| AST IU/L \| \| --- \| | 28 | 89,54 | 64,50 | 19,00 | 409,0 | 74,50 | 84,5 |
| \| FA IU/L \| \| --- \| | 28 | 141,43 | 108,00 | 64,00 | 476,0 | 81,50 | 93,6 |
| \| GGTP Iu/L \| \| --- \| | 28 | 180,43 | 115,50 | 23,00 | 1243,0 | 133,00 | 239,2 |
| \| Glukoza mg/dl \| \| --- \| | 28 | 112,10 | 101,45 | 56,70 | 267,9 | 32,70 | 40,5 |
| \| Mocznik mg/dl \| \| --- \| | 28 | 37,48 | 36,05 | 18,00 | 59,5 | 15,40 | 11,2 |
| \| Kreatynina mg/dL \| \| --- \| | 28 | 0,80 | 0,80 | 0,54 | 1,1 | 0,13 | 0,1 |
| \| Bilirubina mg/dl \| \| --- \| | 28 | 2,09 | 1,41 | 0,43 | 9,7 | 1,27 | 2,0 |
| \| Cholesterol mg/dl \| \| --- \| | 28 | 175,50 | 166,75 | 104,20 | 374,0 | 72,95 | 55,0 |
| \| Triglicerydy mg/dl \| \| --- \| | 28 | 124,03 | 123,60 | 40,00 | 228,0 | 46,85 | 42,0 |
| \| HDL mg/dl \| \| --- \| | 28 | 44,95 | 35,60 | 12,20 | 235,8 | 18,05 | 39,0 |
| \| Pt % \| \| --- \| | 28 | 82,87 | 84,45 | 48,00 | 102,8 | 13,00 | 12,5 |
| \| Białko całkowite g/dl \| \| --- \| | 28 | 7,11 | 7,15 | 4,30 | 9,8 | 0,55 | 1,0 |
| \| Albumina g/dl \| \| --- \| | 28 | 3,42 | 3,40 | 2,00 | 4,6 | 0,80 | 0,7 |
| \| APF ng/ml \| \| --- \| | 28 | 506,23 | 37,79 | 0,74 | 5845,0 | 106,81 | 1294,2 |
| \| CEA ng/ml \| \| --- \| | 28 | 3,02 | 2,51 | 0,17 | 8,8 | 2,15 | 2,2 |
| \| CA 19,9 U/ml \| \| --- \| | 28 | 53,73 | 16,88 | 2,83 | 728,7 | 31,09 | 137,6 |
| \| BMI \| \| --- \| | 28 | 29,50 | 29,00 | 22,00 | 38,0 | 6,50 | 4,3 |
| \| Obwód pasa cm \| \| --- \| | 28 | 103,11 | 102,50 | 80,00 | 130,0 | 14,50 | 12,3 |
| \| HOMA-IR \| \| --- \| | 28 | 5,97 | 2,82 | 1,62 | 25,7 | 3,09 | 6,4 |

| Zmienna | PLT <100 | | | | | | |
| --- | --- | --- | --- | --- | --- | --- | --- |
|  | \| Nważnych \| \| --- \| | \| Średnia \| \| --- \| | \| Mediana \| \| --- \| | \| Minimum \| \| --- \| | \| Maksimum \| \| --- \| | \| Kwartyl. Rozstęp \| \| --- \| | \| Odch.std \| \| --- \| |
| \| Waspina [ng/mL] \| \| --- \| | 39 | 0,55 | 0,20 | 0,030 | 4,9 | 0,496 | 0,9 |
| \| Wisfatyna [ng/mL] \| \| --- \| | 40 | 3,59 | 3,31 | 0,517 | 7,5 | 2,944 | 1,7 |
| \| Iryzyna [µg/mL] \| \| --- \| | 41 | 2,58 | 2,34 | 0,411 | 4,8 | 1,615 | 1,1 |
| \| ESM-1 [ng/mL] \| \| --- \| | 39 | 23,09 | 20,57 | 16,933 | 60,0 | 4,583 | 8,7 |
| \| Betatrofina [ng/mL] \| \| --- \| | 39 | 38,20 | 41,92 | 14,203 | 50,0 | 22,642 | 11,2 |
| \| Insulina [ng/mL] \| \| --- \| | 40 | 1,40 | 1,11 | 0,344 | 3,9 | 0,741 | 1,0 |
| \| Insulina [mIU/mL] \| \| --- \| | 41 | 31,62 | 25,49 | 5,223 | 90,9 | 16,273 | 22,7 |
| \| Wiek( lata) \| \| --- \| | 41 | 59,68 | 59,00 | 20,000 | 88,0 | 10,000 | 12,9 |
| \| WBC 106/ul \| \| --- \| | 40 | 3,58 | 3,36 | 1,780 | 10,0 | 1,770 | 1,5 |
| \| HGB mg/dl \| \| --- \| | 41 | 11,30 | 11,30 | 3,160 | 14,8 | 3,000 | 2,5 |
| \| PLT 106/ul \| \| --- \| | 40 | 62,75 | 62,50 | 18,000 | 99,0 | 35,500 | 21,2 |
| \| ALT IU/L \| \| --- \| | 41 | 56,88 | 45,00 | 15,000 | 222,0 | 43,000 | 40,9 |
| \| AST IU/L \| \| --- \| | 41 | 78,44 | 61,00 | 16,000 | 202,0 | 65,000 | 48,3 |
| \| FA IU/L \| \| --- \| | 41 | 144,15 | 111,00 | 38,000 | 427,0 | 71,000 | 87,6 |
| \| GGTP Iu/L \| \| --- \| | 41 | 129,00 | 86,00 | 21,000 | 965,0 | 83,000 | 157,0 |
| \| Glukoza mg/dl \| \| --- \| | 41 | 126,44 | 119,40 | 76,400 | 281,0 | 49,600 | 45,7 |
| \| Mocznik mg/dl \| \| --- \| | 41 | 44,62 | 34,60 | 14,700 | 229,3 | 23,000 | 38,4 |
| \| Kreatynina mg/dL \| \| --- \| | 41 | 1,78 | 0,75 | 0,490 | 37,4 | 0,480 | 5,7 |
| \| Bilirubina mg/dl \| \| --- \| | 41 | 5,91 | 1,59 | 0,580 | 152,1 | 1,640 | 23,5 |
| \| Cholesterol mg/dl \| \| --- \| | 41 | 137,86 | 143,60 | 2,020 | 233,0 | 37,800 | 40,5 |
| \| Triglicerydy mg/dl \| \| --- \| | 41 | 111,68 | 94,60 | 46,000 | 676,1 | 54,600 | 96,2 |
| \| HDL mg/dl \| \| --- \| | 41 | 40,25 | 38,10 | 7,800 | 177,6 | 16,700 | 24,5 |
| \| Pt % \| \| --- \| | 41 | 68,54 | 68,00 | 40,000 | 96,0 | 11,000 | 12,1 |
| \| Białko całkowite g/dl \| \| --- \| | 41 | 7,16 | 7,10 | 5,400 | 9,8 | 1,100 | 0,9 |
| \| Albumina g/dl \| \| --- \| | 41 | 3,09 | 3,00 | 1,900 | 4,2 | 0,600 | 0,5 |
| \| APF ng/ml \| \| --- \| | 41 | 263,94 | 7,91 | 1,410 | 5845,0 | 33,340 | 1036,2 |
| \| CEA ng/ml \| \| --- \| | 41 | 3,86 | 3,20 | 0,700 | 18,9 | 2,390 | 3,3 |
| \| CA 19,9 U/ml \| \| --- \| | 41 | 19,94 | 14,27 | 2,000 | 78,3 | 16,590 | 17,4 |
| \| BMI \| \| --- \| | 41 | 29,66 | 29,00 | 22,000 | 45,0 | 6,000 | 4,7 |
| \| Obwód pasa cm \| \| --- \| | 41 | 102,49 | 102,00 | 70,000 | 139,0 | 18,000 | 12,9 |
| \| HOMA-IR \| \| --- \| | 41 | 10,00 | 7,06 | 1,377 | 48,2 | 10,011 | 8,8 |

| ZMIENNA | PLT>100 | PLT<100 | TEST | P |
| --- | --- | --- | --- | --- |
| \| Waspina [ng/mL] \| \| --- \| | N | N | U Manna Whitneya | 0,230198589 |
| \| Wisfatyna [ng/mL] \| \| --- \| | T | T | T Studenta | 0,0000928627084 |
| \| Iryzyna [µg/mL] \| \| --- \| | T | N | U Manna Whitneya | 0,169210463 |
| \| ESM-1 [ng/mL] \| \| --- \| | N | N | U Manna Whitneya | 0,969578505 |
| \| Betatrofina [ng/mL] \| \| --- \| | N | N | U Manna Whitneya | 0,000182107368 |
| \| Insulina [ng/mL] \| \| --- \| | N | N | U Manna Whitneya | 0,00117000851 |
| \| Insulina [mIU/mL] \| \| --- \| | N | N | U Manna Whitneya | 0,00259359507 |
| \| Wiek( lata) \| \| --- \| | T | N | U Manna Whitneya | 0,975628468 |
| \| WBC 106/ul \| \| --- \| | T | N | U Manna Whitneya | 0,0000000102987872 |
| \| HGB mg/dl \| \| --- \| | T | N | U Manna Whitneya | 0,0941044795 |
| \| PLT 106/ul \| \| --- \| | N | T | U Manna Whitneya | 0,00000000000313075296 |
| \| ALT IU/L \| \| --- \| | N | N | U Manna Whitneya | 0,695768294 |
| \| AST IU/L \| \| --- \| | N | N | U Manna Whitneya | 0,888241479 |
| \| FA IU/L \| \| --- \| | N | N | U Manna Whitneya | 0,607784255 |
| \| GGTP Iu/L \| \| --- \| | N | N | U Manna Whitneya | 0,322263465 |
| \| Glukoza mg/dl \| \| --- \| | N | N | U Manna Whitneya | 0,1808686 |
| \| Mocznik mg/dl \| \| --- \| | T | N | U Manna Whitneya | 0,638020007 |
| \| Kreatynina mg/dL \| \| --- \| | N | N | U Manna Whitneya | 0,746067263 |
| \| Bilirubina mg/dl \| \| --- \| | T | N | U Manna Whitneya | 0,191030877 |
| \| Cholesterol mg/dl \| \| --- \| | N | T | U Manna Whitneya | 0,0116222042 |
| \| Triglicerydy mg/dl \| \| --- \| | T | N | U Manna Whitneya | 0,0140413644 |
| \| HDL mg/dl \| \| --- \| | N | N | U Manna Whitneya | 0,788053244 |
| \| Pt % \| \| --- \| | T | T | T Studenta | 0,0000107683741 |
| \| Białko całkowite g/dl \| \| --- \| | N | T | U Manna Whitneya | 0,907581403 |
| \| Albumina g/dl \| \| --- \| | T | T | T Studenta | 0,0211309623 |
| \| APF ng/ml \| \| --- \| | N | N | U Manna Whitneya | 0,0505606214 |
| \| CEA ng/ml \| \| --- \| | N | N | U Manna Whitneya | 0,173032183 |
| \| CA 19,9 U/ml \| \| --- \| | N | N | U Manna Whitneya | 0,284958447 |
| \| BMI \| \| --- \| | T | N | U Manna Whitneya | 0,990250113 |
| \| Obwód pasa cm \| \| --- \| | T | T | T Studenta | 0,842371867 |
| \| HOMA-IR \| \| --- \| | N | N | U Manna Whitneya | 0,000990344771 |

1. Glukoza do 100 i pow 100

| Zmienna | Glukoza <100 | | | | | | |
| --- | --- | --- | --- | --- | --- | --- | --- |
|  | \| Nważnych \| \| --- \| | \| Średnia \| \| --- \| | \| Mediana \| \| --- \| | \| Minimum \| \| --- \| | \| Maksimum \| \| --- \| | \| Kwartyl. Rozstęp \| \| --- \| | \| Odch.std \| \| --- \| |
| \| Waspina [ng/mL] \| \| --- \| | 29 | 0,51 | 0,17 | 0,032 | 4,9 | 0,31 | 0,9 |
| \| Wisfatyna [ng/mL] \| \| --- \| | 27 | 4,26 | 4,48 | 0,517 | 8,8 | 2,47 | 1,9 |
| \| Iryzyna [µg/mL] \| \| --- \| | 29 | 2,41 | 2,19 | 0,411 | 4,3 | 1,47 | 1,1 |
| \| ESM-1 [ng/mL] \| \| --- \| | 28 | 22,92 | 20,63 | 16,933 | 55,8 | 4,46 | 7,8 |
| \| Betatrofina [ng/mL] \| \| --- \| | 27 | 31,21 | 27,76 | 10,960 | 50,0 | 23,06 | 13,2 |
| \| Insulina [ng/mL] \| \| --- \| | 29 | 1,05 | 0,79 | 0,334 | 3,9 | 0,58 | 1,0 |
| \| Insulina [mIU/mL] \| \| --- \| | 29 | 24,18 | 18,18 | 7,724 | 90,9 | 13,42 | 22,1 |
| \| Wiek( lata) \| \| --- \| | 29 | 62,10 | 63,00 | 20,000 | 85,0 | 12,00 | 12,7 |
| \| WBC 106/ul \| \| --- \| | 29 | 5,15 | 4,83 | 1,970 | 10,4 | 4,39 | 2,4 |
| \| HGB mg/dl \| \| --- \| | 29 | 11,80 | 12,10 | 8,300 | 16,5 | 3,30 | 2,2 |
| \| PLT 106/ul \| \| --- \| | 29 | 135,00 | 92,00 | 34,000 | 392,0 | 119,00 | 104,1 |
| \| ALT IU/L \| \| --- \| | 29 | 67,10 | 55,00 | 14,000 | 303,0 | 64,00 | 55,5 |
| \| AST IU/L \| \| --- \| | 29 | 101,03 | 73,00 | 22,000 | 409,0 | 66,00 | 82,0 |
| \| FA IU/L \| \| --- \| | 29 | 161,69 | 107,00 | 38,000 | 476,0 | 130,00 | 119,3 |
| \| GGTP Iu/L \| \| --- \| | 29 | 170,97 | 86,00 | 21,000 | 1243,0 | 108,00 | 237,8 |
| \| Glukoza mg/dl \| \| --- \| | 29 | 88,25 | 89,60 | 56,700 | 99,6 | 13,00 | 9,1 |
| \| Mocznik mg/dl \| \| --- \| | 29 | 42,68 | 35,40 | 18,600 | 139,1 | 7,20 | 26,0 |
| \| Kreatynina mg/dL \| \| --- \| | 29 | 0,93 | 0,80 | 0,490 | 2,2 | 0,35 | 0,4 |
| \| Bilirubina mg/dl \| \| --- \| | 29 | 2,38 | 1,78 | 0,430 | 9,7 | 1,82 | 2,0 |
| \| Cholesterol mg/dl \| \| --- \| | 29 | 164,24 | 155,60 | 85,000 | 374,0 | 70,00 | 61,5 |
| \| Triglicerydy mg/dl \| \| --- \| | 29 | 115,34 | 105,00 | 40,000 | 228,0 | 46,60 | 42,0 |
| \| HDL mg/dl \| \| --- \| | 29 | 36,70 | 35,00 | 15,000 | 55,0 | 15,60 | 11,5 |
| \| Pt % \| \| --- \| | 29 | 76,40 | 75,00 | 40,000 | 102,8 | 15,90 | 15,1 |
| \| Białko całkowite g/dl \| \| --- \| | 29 | 7,37 | 7,10 | 5,800 | 9,8 | 0,90 | 1,0 |
| \| Albumina g/dl \| \| --- \| | 29 | 3,28 | 3,10 | 2,000 | 4,5 | 0,70 | 0,6 |
| \| APF ng/ml \| \| --- \| | 29 | 510,25 | 26,70 | 1,010 | 5845,0 | 168,09 | 1271,0 |
| \| CEA ng/ml \| \| --- \| | 29 | 2,77 | 2,40 | 0,165 | 7,3 | 1,55 | 1,8 |
| \| CA 19,9 U/ml \| \| --- \| | 29 | 29,02 | 15,96 | 3,200 | 200,0 | 14,50 | 37,2 |
| \| BMI \| \| --- \| | 29 | 30,62 | 30,00 | 22,000 | 45,0 | 6,00 | 5,0 |
| \| Obwód pasa cm \| \| --- \| | 29 | 105,55 | 108,00 | 70,000 | 139,0 | 15,00 | 14,5 |
| \| HOMA-IR \| \| --- \| | 29 | 5,24 | 3,43 | 1,636 | 20,1 | 3,17 | 4,8 |

| Zmienna | Glukoza >100 | | | | | | |
| --- | --- | --- | --- | --- | --- | --- | --- |
|  | \| Nważnych \| \| --- \| | \| Średnia \| \| --- \| | \| Mediana \| \| --- \| | \| Minimum \| \| --- \| | \| Maksimum \| \| --- \| | \| Kwartyl. Rozstęp \| \| --- \| | \| Odch.std \| \| --- \| |
| \| Waspina [ng/mL] \| \| --- \| | 37 | 0,52 | 0,20 | 0,03 | 5,2 | 0,48 | 0,9 |
| \| Wisfatyna [ng/mL] \| \| --- \| | 39 | 4,25 | 4,51 | 1,14 | 7,5 | 2,80 | 1,7 |
| \| Iryzyna [µg/mL] \| \| --- \| | 40 | 2,96 | 2,81 | 1,22 | 4,8 | 1,96 | 1,1 |
| \| ESM-1 [ng/mL] \| \| --- \| | 39 | 23,22 | 20,48 | 16,12 | 60,0 | 4,78 | 8,9 |
| \| Betatrofina [ng/mL] \| \| --- \| | 38 | 34,32 | 34,85 | 10,90 | 50,0 | 26,55 | 13,5 |
| \| Insulina [ng/mL] \| \| --- \| | 39 | 1,26 | 1,10 | 0,23 | 3,6 | 1,21 | 0,9 |
| \| Insulina [mIU/mL] \| \| --- \| | 40 | 28,61 | 25,20 | 5,22 | 84,1 | 27,68 | 20,1 |
| \| Wiek( lata) \| \| --- \| | 40 | 58,83 | 59,00 | 24,00 | 88,0 | 12,00 | 11,6 |
| \| WBC 106/ul \| \| --- \| | 39 | 4,90 | 4,19 | 1,78 | 9,9 | 3,30 | 2,5 |
| \| HGB mg/dl \| \| --- \| | 40 | 11,80 | 12,20 | 3,16 | 16,1 | 4,05 | 2,7 |
| \| PLT 106/ul \| \| --- \| | 39 | 99,59 | 82,00 | 18,00 | 306,0 | 69,00 | 64,9 |
| \| ALT IU/L \| \| --- \| | 40 | 56,02 | 42,00 | 15,00 | 222,0 | 48,00 | 42,9 |
| \| AST IU/L \| \| --- \| | 40 | 69,83 | 57,50 | 16,00 | 188,0 | 64,00 | 46,2 |
| \| FA IU/L \| \| --- \| | 40 | 129,53 | 110,50 | 55,00 | 363,0 | 61,00 | 57,2 |
| \| GGTP Iu/L \| \| --- \| | 40 | 134,58 | 93,00 | 26,00 | 965,0 | 100,00 | 157,6 |
| \| Glukoza mg/dl \| \| --- \| | 40 | 144,09 | 133,60 | 101,00 | 281,0 | 39,30 | 44,3 |
| \| Mocznik mg/dl \| \| --- \| | 40 | 41,03 | 34,50 | 14,70 | 229,3 | 22,15 | 33,6 |
| \| Kreatynina mg/dL \| \| --- \| | 40 | 1,71 | 0,76 | 0,57 | 37,4 | 0,20 | 5,8 |
| \| Bilirubina mg/dl \| \| --- \| | 40 | 5,80 | 1,51 | 0,48 | 152,1 | 1,29 | 23,8 |
| \| Cholesterol mg/dl \| \| --- \| | 40 | 145,09 | 145,65 | 2,02 | 213,1 | 36,85 | 38,8 |
| \| Triglicerydy mg/dl \| \| --- \| | 40 | 117,67 | 104,95 | 46,00 | 676,1 | 61,80 | 97,5 |
| \| HDL mg/dl \| \| --- \| | 40 | 46,11 | 39,85 | 7,80 | 235,8 | 16,90 | 39,3 |
| \| Pt % \| \| --- \| | 40 | 72,87 | 74,00 | 40,00 | 96,0 | 16,50 | 13,3 |
| \| Białko całkowite g/dl \| \| --- \| | 40 | 6,97 | 7,20 | 4,30 | 8,7 | 1,10 | 0,9 |
| \| Albumina g/dl \| \| --- \| | 40 | 3,18 | 3,10 | 1,90 | 4,6 | 0,80 | 0,6 |
| \| APF ng/ml \| \| --- \| | 40 | 254,97 | 6,68 | 0,74 | 5845,0 | 36,14 | 1047,8 |
| \| CEA ng/ml \| \| --- \| | 40 | 4,06 | 3,29 | 0,70 | 18,9 | 2,95 | 3,4 |
| \| CA 19,9 U/ml \| \| --- \| | 40 | 37,01 | 13,87 | 2,00 | 728,7 | 25,52 | 113,5 |
| \| BMI \| \| --- \| | 40 | 28,85 | 29,00 | 22,00 | 38,0 | 5,00 | 4,0 |
| \| Obwód pasa cm \| \| --- \| | 40 | 100,70 | 99,00 | 80,00 | 120,0 | 17,00 | 10,7 |
| \| HOMA-IR \| \| --- \| | 40 | 10,64 | 7,73 | 1,38 | 48,2 | 11,15 | 9,2 |

| ZMIENNA | GLU<100 | GLU>100 | TEST | P |
| --- | --- | --- | --- | --- |
| \| Waspina [ng/mL] \| \| --- \| | N | N | U Manna Whitneya | 0,979385408 |
| \| Wisfatyna [ng/mL] \| \| --- \| | T | T | T Studenta | 0,97980799 |
| \| Iryzyna [µg/mL] \| \| --- \| | T | N | U Manna Whitneya | 0,0749225486 |
| \| ESM-1 [ng/mL] \| \| --- \| | N | N | U Manna Whitneya | 0,918996003 |
| \| Betatrofina [ng/mL] \| \| --- \| | N | N | U Manna Whitneya | 0,283891505 |
| \| Insulina [ng/mL] \| \| --- \| | N | N | U Manna Whitneya | 0,131902558 |
| \| Insulina [mIU/mL] \| \| --- \| | N | N | U Manna Whitneya | 0,193342361 |
| \| Wiek( lata) \| \| --- \| | N | T | U Manna Whitneya | 0,197537757 |
| \| WBC 106/ul \| \| --- \| | T | N | U Manna Whitneya | 0,581074376 |
| \| HGB mg/dl \| \| --- \| | T | N | U Manna Whitneya | 0,576022745 |
| \| PLT 106/ul \| \| --- \| | N | N | U Manna Whitneya | 0,267071636 |
| \| ALT IU/L \| \| --- \| | N | N | U Manna Whitneya | 0,28746376 |
| \| AST IU/L \| \| --- \| | N | N | U Manna Whitneya | 0,0595286667 |
| \| FA IU/L \| \| --- \| | N | N | U Manna Whitneya | 0,845779966 |
| \| GGTP Iu/L \| \| --- \| | N | N | U Manna Whitneya | 0,817333862 |
| \| Glukoza mg/dl \| \| --- \| | N | N | U Manna Whitneya | 0,0000000000018615687 |
| \| Mocznik mg/dl \| \| --- \| | N | N | U Manna Whitneya | 0,613910789 |
| \| Kreatynina mg/dL \| \| --- \| | N | N | U Manna Whitneya | 0,388072383 |
| \| Bilirubina mg/dl \| \| --- \| | N | N | U Manna Whitneya | 0,990300672 |
| \| Cholesterol mg/dl \| \| --- \| | N | N | U Manna Whitneya | 0,408436246 |
| \| Triglicerydy mg/dl \| \| --- \| | T | N | U Manna Whitneya | 0,422358936 |
| \| HDL mg/dl \| \| --- \| | N | N | U Manna Whitneya | 0,333819399 |
| \| Pt % \| \| --- \| | T | T | T Studenta | 0,309210915 |
| \| Białko całkowite g/dl \| \| --- \| | N | T | U Manna Whitneya | 0,330788141 |
| \| Albumina g/dl \| \| --- \| | T | T | T Studenta | 0,46163795 |
| \| APF ng/ml \| \| --- \| | N | N | U Manna Whitneya | 0,0359929534 |
| \| CEA ng/ml \| \| --- \| | N | N | U Manna Whitneya | 0,0843053692 |
| \| CA 19,9 U/ml \| \| --- \| | N | N | U Manna Whitneya | 0,163943884 |
| \| BMI \| \| --- \| | T | T | T Studenta | 0,105737557 |
| \| Obwód pasa cm \| \| --- \| | T | T | T Studenta | 0,114003967 |
| \| HOMA-IR \| \| --- \| | N | N | U Manna Whitneya | 0,00130264784 |

1. BMI 30 i pow 30

| Zmienna | BMI >30 | | | | | | |
| --- | --- | --- | --- | --- | --- | --- | --- |
|  | \| Nważnych \| \| --- \| | \| Średnia \| \| --- \| | \| Mediana \| \| --- \| | \| Minimum \| \| --- \| | \| Maksimum \| \| --- \| | \| Kwartyl. Rozstęp \| \| --- \| | \| Odch.std \| \| --- \| |
| \| Waspina [ng/mL] \| \| --- \| | 30 | 0,51 | 0,23 | 0,033 | 2,1 | 0,53 | 0,6 |
| \| Wisfatyna [ng/mL] \| \| --- \| | 30 | 4,50 | 4,67 | 1,142 | 7,2 | 2,86 | 1,6 |
| \| Iryzyna [µg/mL] \| \| --- \| | 30 | 2,51 | 2,47 | 0,411 | 4,7 | 1,70 | 1,2 |
| \| ESM-1 [ng/mL] \| \| --- \| | 29 | 24,68 | 20,88 | 17,350 | 55,8 | 3,83 | 9,8 |
| \| Betatrofina [ng/mL] \| \| --- \| | 28 | 31,62 | 30,92 | 10,895 | 50,0 | 19,01 | 12,2 |
| \| Insulina [ng/mL] \| \| --- \| | 30 | 1,00 | 0,84 | 0,334 | 3,1 | 0,69 | 0,7 |
| \| Insulina [mIU/mL] \| \| --- \| | 30 | 23,16 | 19,49 | 7,724 | 71,8 | 15,91 | 16,2 |
| \| Wiek( lata) \| \| --- \| | 30 | 61,20 | 59,00 | 43,000 | 85,0 | 9,00 | 8,7 |
| \| WBC 106/ul \| \| --- \| | 30 | 5,13 | 4,42 | 1,780 | 10,4 | 4,15 | 2,5 |
| \| HGB mg/dl \| \| --- \| | 30 | 12,05 | 12,35 | 5,700 | 16,5 | 3,40 | 2,5 |
| \| PLT 106/ul \| \| --- \| | 30 | 116,40 | 92,50 | 31,000 | 306,0 | 73,00 | 78,4 |
| \| ALT IU/L \| \| --- \| | 30 | 60,03 | 56,00 | 15,000 | 123,0 | 62,00 | 32,8 |
| \| AST IU/L \| \| --- \| | 30 | 89,80 | 66,50 | 16,000 | 258,0 | 98,00 | 64,4 |
| \| FA IU/L \| \| --- \| | 30 | 156,60 | 114,50 | 38,000 | 476,0 | 88,00 | 112,0 |
| \| GGTP Iu/L \| \| --- \| | 30 | 170,37 | 84,50 | 21,000 | 1243,0 | 132,00 | 234,2 |
| \| Glukoza mg/dl \| \| --- \| | 30 | 116,42 | 100,30 | 56,700 | 267,9 | 43,00 | 43,4 |
| \| Mocznik mg/dl \| \| --- \| | 30 | 44,07 | 36,10 | 17,200 | 139,1 | 21,00 | 26,5 |
| \| Kreatynina mg/dL \| \| --- \| | 30 | 0,91 | 0,79 | 0,550 | 2,2 | 0,21 | 0,4 |
| \| Bilirubina mg/dl \| \| --- \| | 30 | 2,45 | 1,72 | 0,580 | 9,7 | 1,00 | 2,1 |
| \| Cholesterol mg/dl \| \| --- \| | 30 | 161,53 | 145,15 | 85,000 | 374,0 | 43,30 | 55,9 |
| \| Triglicerydy mg/dl \| \| --- \| | 30 | 118,93 | 118,65 | 40,000 | 228,0 | 44,80 | 40,1 |
| \| HDL mg/dl \| \| --- \| | 30 | 37,08 | 38,20 | 15,000 | 55,0 | 13,00 | 9,9 |
| \| Pt % \| \| --- \| | 30 | 74,76 | 75,00 | 40,000 | 101,0 | 18,00 | 14,5 |
| \| Białko całkowite g/dl \| \| --- \| | 30 | 7,02 | 7,07 | 4,300 | 8,5 | 0,50 | 0,8 |
| \| Albumina g/dl \| \| --- \| | 30 | 3,26 | 3,20 | 2,000 | 4,4 | 0,80 | 0,6 |
| \| APF ng/ml \| \| --- \| | 30 | 641,83 | 29,95 | 0,740 | 5845,0 | 31,96 | 1611,1 |
| \| CEA ng/ml \| \| --- \| | 30 | 3,51 | 2,63 | 0,165 | 18,9 | 2,99 | 3,5 |
| \| CA 19,9 U/ml \| \| --- \| | 30 | 27,13 | 15,48 | 2,000 | 200,0 | 29,55 | 36,6 |
| \| BMI \| \| --- \| | 30 | 33,63 | 33,00 | 30,000 | 45,0 | 4,00 | 3,2 |
| \| Obwód pasa cm \| \| --- \| | 30 | 112,97 | 112,00 | 99,000 | 139,0 | 8,00 | 8,8 |
| \| HOMA-IR \| \| --- \| | 30 | 7,11 | 4,88 | 1,636 | 25,7 | 5,75 | 6,2 |

| Zmienna | BMI <30 | | | | | | |
| --- | --- | --- | --- | --- | --- | --- | --- |
|  | \| Nważnych \| \| --- \| | \| Średnia \| \| --- \| | \| Mediana \| \| --- \| | \| Minimum \| \| --- \| | \| Maksimum \| \| --- \| | \| Kwartyl. Rozstęp \| \| --- \| | \| Odch.std \| \| --- \| |
| \| Waspina [ng/mL] \| \| --- \| | 36 | 0,52 | 0,12 | 0,029 | 5,2 | 0,34 | 1,16 |
| \| Wisfatyna [ng/mL] \| \| --- \| | 36 | 4,06 | 3,92 | 0,517 | 8,8 | 2,68 | 1,93 |
| \| Iryzyna [µg/mL] \| \| --- \| | 39 | 2,89 | 2,87 | 1,325 | 4,8 | 1,87 | 1,08 |
| \| ESM-1 [ng/mL] \| \| --- \| | 38 | 21,89 | 19,96 | 16,120 | 60,0 | 4,87 | 7,10 |
| \| Betatrofina [ng/mL] \| \| --- \| | 37 | 34,10 | 36,84 | 11,265 | 50,0 | 26,84 | 14,31 |
| \| Insulina [ng/mL] \| \| --- \| | 38 | 1,31 | 1,05 | 0,231 | 3,9 | 1,30 | 1,03 |
| \| Insulina [mIU/mL] \| \| --- \| | 39 | 29,51 | 23,94 | 5,223 | 90,9 | 30,09 | 23,79 |
| \| Wiek( lata) \| \| --- \| | 39 | 59,44 | 59,00 | 20,000 | 88,0 | 14,00 | 14,25 |
| \| WBC 106/ul \| \| --- \| | 38 | 4,90 | 4,23 | 1,860 | 9,9 | 3,70 | 2,48 |
| \| HGB mg/dl \| \| --- \| | 39 | 11,60 | 11,90 | 3,160 | 15,0 | 4,30 | 2,43 |
| \| PLT 106/ul \| \| --- \| | 38 | 113,34 | 84,50 | 18,000 | 392,0 | 75,00 | 90,95 |
| \| ALT IU/L \| \| --- \| | 39 | 61,18 | 44,00 | 14,000 | 303,0 | 45,00 | 58,26 |
| \| AST IU/L \| \| --- \| | 39 | 77,67 | 61,00 | 19,000 | 409,0 | 67,00 | 65,95 |
| \| FA IU/L \| \| --- \| | 39 | 132,62 | 108,00 | 55,000 | 363,0 | 59,00 | 66,93 |
| \| GGTP Iu/L \| \| --- \| | 39 | 134,10 | 96,00 | 26,000 | 965,0 | 107,00 | 159,08 |
| \| Glukoza mg/dl \| \| --- \| | 39 | 123,85 | 109,10 | 79,100 | 281,0 | 43,10 | 44,59 |
| \| Mocznik mg/dl \| \| --- \| | 39 | 39,92 | 33,90 | 14,700 | 229,3 | 15,60 | 33,44 |
| \| Kreatynina mg/dL \| \| --- \| | 39 | 1,74 | 0,76 | 0,490 | 37,4 | 0,26 | 5,86 |
| \| Bilirubina mg/dl \| \| --- \| | 39 | 5,83 | 1,37 | 0,430 | 152,1 | 2,24 | 24,08 |
| \| Cholesterol mg/dl \| \| --- \| | 39 | 146,68 | 150,00 | 2,020 | 220,0 | 52,80 | 44,86 |
| \| Triglicerydy mg/dl \| \| --- \| | 39 | 114,97 | 100,00 | 46,000 | 676,1 | 60,50 | 99,16 |
| \| HDL mg/dl \| \| --- \| | 39 | 46,06 | 36,90 | 7,800 | 235,8 | 19,20 | 40,17 |
| \| Pt % \| \| --- \| | 39 | 74,04 | 70,00 | 40,000 | 102,8 | 18,00 | 13,97 |
| \| Białko całkowite g/dl \| \| --- \| | 39 | 7,23 | 7,20 | 5,400 | 9,8 | 1,20 | 1,05 |
| \| Albumina g/dl \| \| --- \| | 39 | 3,19 | 3,10 | 1,900 | 4,6 | 0,60 | 0,56 |
| \| APF ng/ml \| \| --- \| | 39 | 147,21 | 7,36 | 1,410 | 2840,9 | 59,33 | 506,03 |
| \| CEA ng/ml \| \| --- \| | 39 | 3,53 | 3,11 | 0,700 | 12,2 | 2,15 | 2,40 |
| \| CA 19,9 U/ml \| \| --- \| | 39 | 38,67 | 15,06 | 2,000 | 728,7 | 17,47 | 114,82 |
| \| BMI \| \| --- \| | 39 | 26,48 | 27,00 | 22,000 | 29,0 | 3,00 | 2,31 |
| \| Obwód pasa cm \| \| --- \| | 39 | 94,87 | 96,00 | 70,000 | 116,0 | 9,00 | 8,83 |
| \| HOMA-IR \| \| --- \| | 39 | 9,34 | 5,61 | 1,377 | 48,2 | 11,67 | 9,25 |

| ZMIENNA | BMI>30 | BMI<30 | TEST | P |
| --- | --- | --- | --- | --- |
| \| Waspina [ng/mL] \| \| --- \| | N | N | U Manna Whitneya | 0,0466338128 |
| \| Wisfatyna [ng/mL] \| \| --- \| | T | T | T Studenta | 0,327199412 |
| \| Iryzyna [µg/mL] \| \| --- \| | T | N | U Manna Whitneya | 0,181043195 |
| \| ESM-1 [ng/mL] \| \| --- \| | N | N | U Manna Whitneya | 0,0841074482 |
| \| Betatrofina [ng/mL] \| \| --- \| | T | N | U Manna Whitneya | 0,446215904 |
| \| Insulina [ng/mL] \| \| --- \| | N | N | U Manna Whitneya | 0,338452954 |
| \| Insulina [mIU/mL] \| \| --- \| | N | N | U Manna Whitneya | 0,449329403 |
| \| Wiek( lata) \| \| --- \| | T | T | T Studenta | 0,552567761 |
| \| WBC 106/ul \| \| --- \| | N | N | U Manna Whitneya | 0,656574595 |
| \| HGB mg/dl \| \| --- \| | T | N | U Manna Whitneya | 0,486422031 |
| \| PLT 106/ul \| \| --- \| | N | N | U Manna Whitneya | 0,656574595 |
| \| ALT IU/L \| \| --- \| | N | N | U Manna Whitneya | 0,357601751 |
| \| AST IU/L \| \| --- \| | N | N | U Manna Whitneya | 0,478873608 |
| \| FA IU/L \| \| --- \| | N | N | U Manna Whitneya | 0,985513756 |
| \| GGTP Iu/L \| \| --- \| | N | N | U Manna Whitneya | 0,671814606 |
| \| Glukoza mg/dl \| \| --- \| | N | N | U Manna Whitneya | 0,403597855 |
| \| Mocznik mg/dl \| \| --- \| | N | N | U Manna Whitneya | 0,278650684 |
| \| Kreatynina mg/dL \| \| --- \| | N | N | U Manna Whitneya | 0,743802076 |
| \| Bilirubina mg/dl \| \| --- \| | N | N | U Manna Whitneya | 0,396818226 |
| \| Cholesterol mg/dl \| \| --- \| | N | T | U Manna Whitneya | 0,628257023 |
| \| Triglicerydy mg/dl \| \| --- \| | T | N | U Manna Whitneya | 0,0901452592 |
| \| HDL mg/dl \| \| --- \| | T | N | U Manna Whitneya | 0,636871811 |
| \| Pt % \| \| --- \| | T | T | T Studenta | 0,83555647 |
| \| Białko całkowite g/dl \| \| --- \| | N | T | U Manna Whitneya | 0,545028253 |
| \| Albumina g/dl \| \| --- \| | T | T | T Studenta | 0,674414398 |
| \| APF ng/ml \| \| --- \| | N | N | U Manna Whitneya | 0,219218258 |
| \| CEA ng/ml \| \| --- \| | N | N | U Manna Whitneya | 0,442108234 |
| \| CA 19,9 U/ml \| \| --- \| | N | N | U Manna Whitneya | 0,752975741 |
| \| BMI \| \| --- \| | N | N | U Manna Whitneya | 0,00000000000149634599 |
| \| Obwód pasa cm \| \| --- \| | T | T | T Studenta | 0,00000000000340309231 |
| \| HOMA-IR \| \| --- \| | N | N | U Manna Whitneya | 0,434953938 |
